# Human genome meeting 2016: Houston, TX, USA. 28 February - 2 March 2016

**DOI:** 10.1186/s40246-016-0063-5

**Published:** 2016-05-26

**Authors:** A. K. Srivastava, Y. Wang, R. Huang, C. Skinner, T. Thompson, L. Pollard, T. Wood, F. Luo, R. Stevenson, R. Polimanti, J. Gelernter, X. Lin, I. Y. Lim, Y. Wu, A. L. Teh, L. Chen, I. M. Aris, S. E. Soh, M. T. Tint, J. L. MacIsaac, F. Yap, K. Kwek, S. M. Saw, M. S. Kobor, M. J. Meaney, K. M. Godfrey, Y. S. Chong, J. D. Holbrook, Y. S. Lee, P. D. Gluckman, N. Karnani, GUSTO study group, A. Kapoor, D. Lee, A. Chakravarti, C. Maercker, F. Graf, M. Boutros, G. Stamoulis, F. Santoni, P. Makrythanasis, A. Letourneau, M. Guipponi, N. Panousis, M. Garieri, P. Ribaux, E. Falconnet, C. Borel, S. E. Antonarakis, S. Kumar, J. Curran, J. Blangero, S. Chatterjee, A. Kapoor, J. Akiyama, D. Auer, C. Berrios, L. Pennacchio, A. Chakravarti, T. R. Donti, G. Cappuccio, M. Miller, P. Atwal, A. Kennedy, A. Cardon, C. Bacino, L. Emrick, J. Hertecant, F. Baumer, B. Porter, M. Bainbridge, P. Bonnen, B. Graham, R. Sutton, Q. Sun, S. Elsea, Z. Hu, P. Wang, Y. Zhu, J. Zhao, M. Xiong, David A. Bennett, A. Hidalgo-Miranda, S. Romero-Cordoba, S. Rodriguez-Cuevas, R. Rebollar-Vega, E. Tagliabue, M. Iorio, E. D’Ippolito, S. Baroni, B. Kaczkowski, Y. Tanaka, H. Kawaji, A. Sandelin, R. Andersson, M. Itoh, T. Lassmann, The FANTOM5 Consortium, Y. Hayashizaki, P. Carninci, A. R. R. Forrest, C. A. Semple, E. A. Rosenthal, B. Shirts, L. Amendola, C. Gallego, M. Horike-Pyne, A. Burt, P. Robertson, P. Beyers, C. Nefcy, D. Veenstra, F. Hisama, R. Bennett, M. Dorschner, D. Nickerson, J. Smith, K. Patterson, D. Crosslin, R. Nassir, N. Zubair, T. Harrison, U. Peters, G. Jarvik, NHLBI GO Exome Sequencing Project, F. Menghi, K. Inaki, X. Woo, P. Kumar, K. Grzeda, A. Malhotra, H. Kim, D. Ucar, P. Shreckengast, K. Karuturi, J. Keck, J. Chuang, E. T. Liu, B. Ji, A. Tyler, G. Ananda, G. Carter, H. Nikbakht, M. Montagne, M. Zeinieh, A. Harutyunyan, M. Mcconechy, N. Jabado, P. Lavigne, J. Majewski, J. B. Goldstein, M. Overman, G. Varadhachary, R. Shroff, R. Wolff, M. Javle, A. Futreal, D. Fogelman, L. Bravo, W. Fajardo, H. Gomez, C. Castaneda, C. Rolfo, J. A. Pinto, K. C. Akdemir, L. Chin, A. Futreal, ICGC PCAWG Structural Alterations Group, S. Patterson, C. Statz, S. Mockus, S. N. Nikolaev, X. I. Bonilla, L. Parmentier, B. King, F. Bezrukov, G. Kaya, V. Zoete, V. Seplyarskiy, H. Sharpe, T. McKee, A. Letourneau, P. Ribaux, K. Popadin, N. Basset-Seguin, R. Ben Chaabene, F. Santoni, M. Andrianova, M. Guipponi, M. Garieri, C. Verdan, K. Grosdemange, O. Sumara, M. Eilers, I. Aifantis, O. Michielin, F. de Sauvage, S. Antonarakis, S. Likhitrattanapisal, S. Lincoln, A. Kurian, A. Desmond, S. Yang, Y. Kobayashi, J. Ford, L. Ellisen, T. L. Peters, K. R. Alvarez, E. F. Hollingsworth, D. H. Lopez-Terrada, A. Hastie, Z. Dzakula, A. W. Pang, E. T. Lam, T. Anantharaman, M. Saghbini, H. Cao, BioNano Genomics, C. Gonzaga-Jauregui, L. Ma, A. King, E. Berman Rosenzweig, U. Krishnan, J. G. Reid, J. D. Overton, F. Dewey, W. K. Chung, K. Small, A. DeLuca, F. Cremers, R. A. Lewis, V. Puech, B. Bakall, R. Silva-Garcia, K. Rohrschneider, M. Leys, F. S. Shaya, E. Stone, N. L. Sobreira, F. Schiettecatte, H. Ling, E. Pugh, D. Witmer, K. Hetrick, P. Zhang, K. Doheny, D. Valle, A. Hamosh, S. N. Jhangiani, Z. Coban Akdemir, M. N. Bainbridge, W. Charng, W. Wiszniewski, T. Gambin, E. Karaca, Y. Bayram, M. K. Eldomery, J. Posey, H. Doddapaneni, J. Hu, V. R. Sutton, D. M. Muzny, E. A. Boerwinkle, D. Valle, J. R. Lupski, R. A. Gibbs, S. Shekar, W. Salerno, A. English, A. Mangubat, J. Bruestle, A. Thorogood, B. M. Knoppers, Global Alliance for Genomics and Health - Regulatory and Ethics Working Group, H. Takahashi, K. R. Nitta, A. Kozhuharova, A. M. Suzuki, H. Sharma, D. Cotella, C. Santoro, S. Zucchelli, S. Gustincich, P. Carninci, J. J. Mulvihill, G. Baynam, W. Gahl, S. C. Groft, K. Kosaki, P. Lasko, B. Melegh, D. Taruscio, R. Ghosh, S. Plon, S. Scherer, X. Qin, R. Sanghvi, K. Walker, T. Chiang, D. Muzny, L. Wang, J. Black, E. Boerwinkle, R. Weinshilboum, R. Gibbs, T. Karpinets, T. Calderone, K. Wani, X. Yu, C. Creasy, C. Haymaker, M. Forget, V. Nanda, J. Roszik, J. Wargo, L. Haydu, X. Song, A. Lazar, J. Gershenwald, M. Davies, C. Bernatchez, J. Zhang, A. Futreal, S. Woodman, E. J. Chesler, T. Reynolds, J. A. Bubier, C. Phillips, M. A. Langston, E. J. Baker, M. Xiong, L. Ma, N. Lin, C. Amos, N. Lin, P. Wang, Y. Zhu, J. Zhao, V. Calhoun, M. Xiong, O. Dobretsberger, M. Egger, F. Leimgruber, S. Sadedin, A. Oshlack, Melbourne Genomics Health Alliance, V. A. A. Antonio, N. Ono, Clark Kendrick C. Go, Z. Ahmed, M. Bolisetty, S. Zeeshan, E. Anguiano, D. Ucar, A. Sarkar, M. R. Nandineni, C. Zeng, J. Shao, H. Cao, A. Hastie, A. W. Pang, E. T. Lam, T. Liang, K. Pham, M. Saghbini, Z. Dzakula, Y. Chee-Wei, L. Dongsheng, W. Lai-Ping, D. Lian, R. O. Twee Hee, Y. Yunus, F. Aghakhanian, S. S. Mokhtar, C. V. Lok-Yung, J. Bhak, M. Phipps, X. Shuhua, T. Yik-Ying, V. Kumar, H. Boon-Peng, I. Campbell, M. -A. Young, P. James, Lifepool, M. Rain, G. Mohammad, R. Kukreti, Q. Pasha, A. R. Akilzhanova, C. Guelly, Z. Abilova, S. Rakhimova, A. Akhmetova, U. Kairov, S. Trajanoski, Z. Zhumadilov, M. Bekbossynova, C. Schumacher, S. Sandhu, T. Harkins, V. Makarov, H. Doddapaneni, R. Glenn, Z. Momin, B. Dilrukshi, H. Chao, Q. Meng, B. Gudenkauf, R. Kshitij, J. Jayaseelan, C. Nessner, S. Lee, K. Blankenberg, L. Lewis, J. Hu, Y. Han, H. Dinh, S. Jireh, K. Walker, E. Boerwinkle, D. Muzny, R. Gibbs, J. Hu, K. Walker, C. Buhay, X. Liu, Q. Wang, R. Sanghvi, H. Doddapaneni, Y. Ding, N. Veeraraghavan, Y. Yang, E. Boerwinkle, A. L. Beaudet, C. M. Eng, D. M. Muzny, R. A. Gibbs, K. C. C. Worley, Y. Liu, D. S. T. Hughes, S. C. Murali, R. A. Harris, A. C. English, X. Qin, O. A. Hampton, P. Larsen, C. Beck, Y. Han, M. Wang, H. Doddapaneni, C. L. Kovar, W. J. Salerno, A. Yoder, S. Richards, J. Rogers, J. R. Lupski, D. M. Muzny, R. A. Gibbs, Q. Meng, M. Bainbridge, M. Wang, H. Doddapaneni, Y. Han, D. Muzny, R. Gibbs, R. A. Harris, M. Raveenedran, C. Xue, M. Dahdouli, L. Cox, G. Fan, B. Ferguson, J. Hovarth, Z. Johnson, S. Kanthaswamy, M. Kubisch, M. Platt, D. Smith, E. Vallender, R. Wiseman, X. Liu, J. Below, D. Muzny, R. Gibbs, F. Yu, J. Rogers, J. Lin, Y. Zhang, Z. Ouyang, A. Moore, Z. Wang, J. Hofmann, M. Purdue, R. Stolzenberg-Solomon, S. Weinstein, D. Albanes, C. S. Liu, W. L. Cheng, T. T. Lin, Q. Lan, N. Rothman, S. Berndt, E. S. Chen, H. Bahrami, A. Khoshzaban, S. Heidari Keshal, H. Bahrami, A. Khoshzaban, S. Heidari Keshal, K. K. R. Alharbi, M. Zhalbinova, A. Akilzhanova, S. Rakhimova, M. Bekbosynova, S. Myrzakhmetova, M. Matar, N. Mili, R. Molinari, Y. Ma, S. Guerrier, N. Elhawary, M. Tayeb, N. Bogari, N. Qotb, S. A. McClymont, P. W. Hook, L. A. Goff, A. McCallion, Y. Kong, J. R. Charette, W. L. Hicks, J. K. Naggert, L. Zhao, P. M. Nishina, B. M. Edrees, M. Athar, F. A. Al-Allaf, M. M. Taher, W. Khan, A. Bouazzaoui, N. A. Harbi, R. Safar, H. Al-Edressi, A. Anazi, N. Altayeb, M. A. Ahmed, K. Alansary, Z. Abduljaleel, A. Kratz, P. Beguin, S. Poulain, M. Kaneko, C. Takahiko, A. Matsunaga, S. Kato, A. M. Suzuki, N. Bertin, T. Lassmann, R. Vigot, P. Carninci, C. Plessy, T. Launey, D. Graur, D. Lee, A. Kapoor, A. Chakravarti, J. Friis-Nielsen, J. M. Izarzugaza, S. Brunak, A. Chakraborty, J. Basak, A. Mukhopadhyay, B. S. Soibam, D. Das, N. Biswas, S. Das, S. Sarkar, A. Maitra, C. Panda, P. Majumder, H. Morsy, A. Gaballah, M. Samir, M. Shamseya, H. Mahrous, A. Ghazal, W. Arafat, M. Hashish, J. J. Gruber, N. Jaeger, M. Snyder, K. Patel, S. Bowman, T. Davis, D. Kraushaar, A. Emerman, S. Russello, N. Henig, C. Hendrickson, K. Zhang, M. Rodriguez-Dorantes, C. D. Cruz-Hernandez, C. D. P. Garcia-Tobilla, S. Solorzano-Rosales, N. Jäger, J. Chen, R. Haile, M. Hitchins, J. D. Brooks, M. Snyder, S. Jiménez-Morales, M. Ramírez, J. Nuñez, V. Bekker, Y. Leal, E. Jiménez, A. Medina, A. Hidalgo, J. Mejía, V. Halytskiy, J. Naggert, G. B. Collin, K. DeMauro, R. Hanusek, P. M. Nishina, K. Belhassa, K. Belhassan, L. Bouguenouch, I. Samri, H. Sayel, FZ. moufid, I. El Bouchikhi, S. Trhanint, H. Hamdaoui, I. Elotmani, I. Khtiri, O. Kettani, L. Quibibo, M. Ahagoud, M. Abbassi, K. Ouldim, A. V. Marusin, A. N. Kornetov, M. Swarovskaya, K. Vagaiceva, V. Stepanov, E. M. Cutiongco De La Paz, R. Sy, J. Nevado, P. Reganit, L. Santos, J. D. Magno, F. E. Punzalan, D. Ona, E. Llanes, R. L. Santos-Cortes, R. Tiongco, J. Aherrera, L. Abrahan, P. Pagauitan-Alan, The Philippine Cardiogenomics Study Group, K. H. Morelli, J. S. Domire, N. Pyne, S. Harper, R. Burgess, M. Zhalbinova, A. Akilzhanova, S. Rakhimova, M. Bekbosynova, S. Myrzakhmetova, M. A. Gari, A. Dallol, H. Alsehli, A. Gari, M. Gari, A. Abuzenadah, M. Thomas, M. Sukhai, S. Garg, M. Misyura, T. Zhang, A. Schuh, T. Stockley, S. Kamel-Reid, S. Sherry, C. Xiao, D. Slotta, K. Rodarmer, M. Feolo, M. Kimelman, G. Godynskiy, C. O’Sullivan, E. Yaschenko, C. Xiao, E. Yaschenko, S. Sherry, C. Rangel-Escareño, H. Rueda-Zarate, I. A. Tayubi, R. Mohammed, on behalf of 1, I. Ahmed, T. Ahmed, S. Seth, S. Amin, X. Song, X. Mao, H. Sun, R. G. Verhaak, A. Futreal, J. Zhang, S. J. Whiite, T. Chiang, A. English, J. Farek, Z. Kahn, W. Salerno, N. Veeraraghavan, E. Boerwinkle, R. Gibbs, T. Kasukawa, M. Lizio, J. Harshbarger, S. Hisashi, J. Severin, A. Imad, S. Sahin, T. C. Freeman, K. Baillie, A. Sandelin, P. Carninci, A. R. R. Forrest, H. Kawaji, The FANTOM Consortium, W. Salerno, A. English, S. N. Shekar, A. Mangubat, J. Bruestle, E. Boerwinkle, R. A. Gibbs, A. H. Salem, M. Ali, A. Ibrahim, M. Ibrahim, H. A. Barrera, L. Garza, J. A. Torres, V. Barajas, A. Ulloa-Aguirre, D. Kershenobich, Shahroj Mortaji, Pedro Guizar, Eliezer Loera, Karen Moreno, Adriana De León, Daniela Monsiváis, Jackeline Gómez, Raquel Cardiel, J. C. Fernandez-Lopez, V. Bonifaz-Peña, C. Rangel-Escareño, A. Hidalgo-Miranda, A. V. Contreras, L. Polfus, CHARGE and NHLBI Exome Sequence Project Working Groups, X. Wang, V. Philip, G. Carter, A. A. Abuzenadah, M. Gari, R. Turki, A. Dallol, A. Uyar, A. Kaygun, S. Zaman, E. Marquez, J. George, D. Ucar, C. L. Hendrickson, A. Emerman, D. Kraushaar, S. Bowman, N. Henig, T. Davis, S. Russello, K. Patel, D. B. Starr, M. Baird, B. Kirkpatrick, K. Sheets, R. Nitsche, L. Prieto-Lafuente, M. Landrum, J. Lee, W. Rubinstein, D. Maglott, P. K. R. Thavanati, A. Escoto de Dios, R. E. Navarro Hernandez, M. E. Aguilar Aldrate, M. R. Ruiz Mejia, K. R. R. Kanala, Z. Abduljaleel, W. Khan, F. A. Al-Allaf, M. Athar, M. M. Taher, N. Shahzad, A. Bouazzaoui, E. Huber, A. Dan, F. A. Al-Allaf, W. Herr, G. Sprotte, J. Köstler, A. Hiergeist, A. Gessner, R. Andreesen, E. Holler, F. Al-Allaf, A. Alashwal, Z. Abduljaleel, M. Taher, A. Bouazzaoui, H. Abalkhail, A. Al-Allaf, R. Bamardadh, M. Athar, O. Filiptsova, M. Kobets, Y. Kobets, I. Burlaka, I. Timoshyna, O. Filiptsova, M. N. Kobets, Y. Kobets, I. Burlaka, I. Timoshyna, O. Filiptsova, M. N. Kobets, Y. Kobets, I. Burlaka, I. Timoshyna, F. A. Al-allaf, M. T. Mohiuddin, A. Zainularifeen, A. Mohammed, H. Abalkhail, T. Owaidah, A. Bouazzaoui

**Affiliations:** JCSRI, Greenwood Genetic Center, Greenwood, SC USA; School of Computing, Clemson University, Clemson, SC USA; Biochemical Genetics Laboratory, Greenwood Genetic Center, Greenwood, SC USA; Department Psychiatry, Yale Sch Med and VA CT Healthcare Center, West Haven, CT USA; Department Genetics, Yale Sch Med and VA CT Healthcare Center, West Haven, CT USA; Department Neurobiology, Yale Sch Med and VA CT Healthcare Center, West Haven, CT USA; Singapore Institute for Clinical Sciences, Singapore, Singapore; National University of Singapore, Singapore, Singapore; University of British Columbia, Vancouver, British Columbia Canada; KK Women’s and Children’s Hospital, Singapore, Singapore; University of Southampton and University Hospital Southampton NHS Foundation Trust, Southampton, UK; University of Auckland, Auckland, New Zealand; McKusick-Nathans Institute of Genetic Medicine, Johns Hopkins University School of Medicine, Baltimore, MD USA; Esslingen University of Applied Sciences, Esslingen, Germany; German Cancer Research Center, Heidelberg, Germany; Department of Genetic Medicine and Development, University of Geneva Medical School, Geneva, Switzerland; Geneva University Hospitals-HUG, Service of Genetic Medicine, Geneva, Switzerland; iGE3 Institute of Genetics and Genomics of Geneva, University of Geneva Medical School, Geneva, Switzerland; South Texas Diabetes and Obesity Institute, School of Medicine, University of Texas Rio-Grande Valley, Edinburg, TX USA; South Texas Diabetes and Obesity Institute, School of Medicine, University of Texas Rio-Grande Valley, Brownsville, TX USA; Institute of Genetic Medicine, Johns Hopkins University, Baltimore, MD USA; Genomics Division, Lawrence Berkeley National Laboratory, Berkeley, CA USA; Molecular and Human Genetics, Baylor College of Medicine, Houston, TX USA; Department of Translational Medical Sciences, Federico II University, Naples, Italy; Metabolon Inc, Durham, NC USA; Section of Pediatric Neurology and Neuroscience, Baylor College of Medicine, Houston, TX USA; Tawam Hospital, Abu Dhabi, United Arab Emirates; Stanford Medical School, Stanford, CA USA; School of Public Health, Houston Health Science Center, Houston, TX USA; University of Texas, Houston Health Science Center, Houston, TX USA; Tulane University, New Orleans, LO USA; Rush Alzheimer’s Disease Center, Rush University, Chicago, IL USA; Cancer Genomics Laboratory, National Institute of Genomic Medicine (INMEGEN), Mexico City, Mexico; FUCAM, Mexico City, Mexico; National Tumor Institute, Milan, Italy; Division of Genomic Technologies, RIKEN Center for Life Science Technologies, Yokohama, Japan; Preventive Medicine and Applied Genomics unit, RIKEN Advanced Center for Computing and Communication, Yokohama, Japan; Department of Biology, University of Copenhagen, Copenhagen, Denmark; Telethon Kids Institute, The University of Western Australia, Perth, Australia; RIKEN Preventive Medicine & Diagnosis Innovation Program, Wako, Japan; Harry Perkins Institute of Medical Research, The University of Western Australia, Nedlands, Australia; MRC Human Genetics Unit, MRC Institute of Genetics and Molecular Medicine, Edinburgh, UK; Univ of Washington, Seattle, WA USA; University of Michigan, Ann Arbor, MI USA; University California, Davis, CA USA; Fred Hutch, Seattle, WA USA; The Jackson Laboratory, Farmington, CT USA; The Jackson Laboratory, Sacramento, CA USA; The Jackson Laboratory, Bar Harbor, ME USA; Human Genetics, McGill University, Montreal, Quebec Canada; Biochemistry, Sherbrooke University, Sherbrooke, Quebec Canada; Pediatrics, McGill University, Montreal, Quebec Canada; Genomic Medicine, MD Anderson Cancer Center, Houston, TX USA; Gastrointestinal Medical Oncology, MD Anderson Cancer Center, Houston, TX USA; Escuela de Medicina Humana, Universidad Privada San Juan Bautista, Lima, Peru; Unidad de Investigación Básica y Traslacional, Oncosalud-AUNA, Lima, Peru; Oncology Department, University Hospital Antwerp, Antwerp, Belgium; Genomic Medicine, MD Anderson Cancer Center, Houston, TX USA; University of Texas System, Houston, TX USA; The Jackson Laboratory for Genomic Medicine, Farmington, CT USA; Department of Genetic Medicine and Development, University of Geneva, Geneva, Switzerland; Department of Dermatology, Hospital of Valais, Sion, Switzerland; Department of Pathology, NYU School of Medicine, New York, NY USA; Department of Physics, University of Connecticut, Connecticut, USA; Department of Dermatology, University Hospitals of Geneva, Geneva, Switzerland; Swiss Institute of Bioinformatics, Swiss Institute of Bioinformatics, Lausanne, Switzerland; Institute of Information Transmission Problems, Russian Academy of Sciences, Moscow, Russian Federation; Department of Molecular Oncology, Genentech Inc, San Francisco, CA USA; Service of Clinical Pathology, University Hospitals of Geneva, Geneva, Switzerland; Department of Genetic Medicine and Development, University of Geneva Medical School, Geneva, Switzerland; University of Paris 7, Hospital of Saint-Louis, Paris, France; Service of Genetic Medicine, University Hospitals of Geneva, Geneva, Switzerland; Department of Biochemistry and Molecular Biology, University of Würzburg, Würzburg, Germany; Department of Biology, Faculty of Science, Mahidol University, Bangkok, Thailand; Invitae, San Francisco, CA USA; Stanford Medical Center, Palo Alto, CA USA; Massachusetts General Hospital, Boston, MA USA; Stanford Medical Center, San Francisco, CA USA; Pathology & Immunology, Baylor College of Medicine, Houston, TX USA; Pathology, Texas Children’s Hospital, Houston, TX USA; BioNano Genomics, Inc, San Diego, CA USA; Regeneron Genetics Center, Regeneron Pharmaceuticals, Tarrytown, New York, NY USA; Department of Pediatrics, New York, NY USA; Department of Medicine, Columbia University Medical Center, New York, NY USA; Molecular Insight Research Foundation, Glendale, ᅟ; Ophthalmology, University of Iowa, Iowa City, IA USA; Biology, Raboud University Medical Center, Nijmegen, Netherlands; Ophthalmology, Baylor College of Medicine, Houston, TX USA; Service d’Exploration de la vision et Neuro-ophtalmologie CHRU, Service d’Exploration de la vision et Neuro-ophtalmologie CHRU, Lille, France; Associated Retina Consultants, University of Arizona College of Medicine, Phoenix, TX USA; University of Heidelberg, Heidelberg, Germany; WVU Eye Institute, Morgantown, WV USA; University of Iowa, Iowa City, IA USA; Johns Hopkins University School of Medicine, Baltimore, MD USA; FS Consulting, Salem, MA USA; Center for Inherited Disease Research, JHUSOM, Baltimore, MD USA; Human Genome Sequencing Center, Baylor College of Medicine, Houston, TX USA; Department of Molecular and Human Genetics, Baylor College of Medicine, Houston, TX USA; McKusick-Nathans Institute of Genetic Medicine, Johns Hopkins School of Medicine, Baltimore, MD USA; Spiral Genetics, Seattle, WA USA; Human Genome Sequencing Center, Baylor College of Medicine, Houston, TX USA; Centre of Genomics and Policy, McGill University, Montreal, Quebec Canada; Center for Life Science Technologies, Division of Genomic Technologies, RIKEN, Yokohama, Japan; Dipartimento di Scienze della Salute, Universita’ del Piemonte Orientale, Novara, Italy; Area of Neuroscience, SISSA, International School for Advanced Studies, Trieste, Italy; Division of Genomic Medicine, National Human Genome Research Institute, Bethesda, MD USA; Office of Population Health, Department of Health, Perth, Australia; Undiagnosed Diseases Program, National Human Genome Research Institute, Bethesda, MD USA; National Center for Advancing Translational Sciences, National Institutes of Health, Bethesda, MD USA; Center for Medical Genetics, Keio University School of Medicine, Tokyo, Japan; Department of Biology, McGill University, Montreal, Quebec Canada; Department of Medical Genetics, University of Pecs, Pecs, Hungary; National Center for Rare Diseases, Istituto Superiore di Sanita, Rome, Italy; Pediatrics-Oncology, Baylor College of Medicine, Houston, TX USA; Human Genome Sequencing Center, Baylor College of Medicine, Houston, TX USA; Department of Molecular Pharmacology and Experimental Therapeutics, Mayo Clinic, Rochester, NY USA; Department of Psychiatry, Mayo Clinic, Rochester, NY USA; Department of Pharmacology, Mayo Clinic, Rochester, NY USA; MD Anderson Cancer Center, Houston, USA; The Jackson Laboratory, Bar Harbor, ME USA; Baylor University, Waco, TX USA; University of Tennessee, Knoxville, TN USA; University of Texas School of Public Health, Houston, TX USA; Geisel School of Medicine at Dartmouth, Hanover, NH USA; Biostatistics, University of Texas Health Science Center at Houston, Houston, TX USA; Tulane University, New Orleans, LO USA; University of New Mexico, Albuquerque, NM USA; University of Texas Health Science Center at Houston, Houston, TX USA; EPS Software Corp, Spring, TX USA; Bioinformatics, Murdoch Childrens Research Institute, Parkville, Australia; Computational Systems Biology Laboratory, Nara Institute of Science and Technology, Ikoma-cho, Japan; The Jackson Laboratory for Genomic Medicine, Farmington, CT USA; Laboratory of Genomics and Profiling Applications, Centre for DNA Fingerprinting and Diagnostics, Hyderabad, India; Beijing Institute of Genomics, Chinese Academy of Sciences, Beijing, China; BioNano Genomics, Inc, San Diego, CA USA; Biotechnology Research Institute, Universiti Malaysia Sabah, Kota Kinabalu, Malaysia; Shanghai Institutes for Biological Sciences, Chinese Academy of Sciences Shanghai, Shanghai, China; Saw Swee Hock School of Public Health, National University of Singapore, Singapore, Singapore; Institute of Medical Molecular Biotechnology, Universiti Teknologi MARA, Sungai Buloh, ᅟ; Jeffrey Cheah School of Medicine and Health Sciences, Monash University Sunway Campus, Petaling Jaya, Malaysia; Personal Genomics Institute, Genome Research Foundation, Suwon, Republic Of Korea; Jeffrey Cheah School of Medicine and Health Sciences, Monash University Sunway Campu, Petaling Jaya, Malaysia; Shanghai Institutes for Biological Sciences, Chinese Academy of Sciences Shanghai, Shanghai, China; UCSI University, Kuala Lumpur, Kuala Lumpur, Malaysia; Research Division, Peter Maccallum Cancer Centre, East Melbourne, Australia; Familial Cancer Centre, Peter Maccallum Cancer Centre, East Melbourne, Australia; Genomics and Molecular Medicine, CSIR-Institute of Genomics and Integrative Biology, New Delhi, India; Department of Medicine, Sonam Norbu Memorial Hospital, Leh, Ladakh India; Nazarbayev University, National Laboratory Astana, Center for Life Sciences, Astana, Kazakhstan; Center of Medical Research, Medical University of Graz, Graz, Austria; National Scientific Cardiac Surgery Center, Astana, Kazakhstan; Swift Biosciences Inc, Ann Arbor, MI USA; Human Genome Sequencing Center, Baylor College of Medicine, Houston, TX USA; Human Genome Sequencing Center, Baylor College of Medicine, Houston, TX USA; Department of Molecular and Human Genetics, Baylor College of Medicine, Houston, TX USA; Human Genetics Center, University of Texas Health Science Center at Houston, Houston, TX USA; Human Genome Sequencing Center, Molecular and Human Genetics, Baylor College of Medicine, Houston, TX USA; Department of Biology, Duke University, Durham, NC USA; Molecular and Human Genetics, Baylor College of Medicine, Houston, TX USA; HGSC, Baylor College of Medicine, Houston, TX USA; Molecular and Human Genetics, Baylor College of Medicine, Houston, TX USA; Human Genome Sequencing Center, Baylor College of Medicine, Houston, TX USA; Genetics, Southwest National Primate Research Center, San Antonio, TX USA; Human Genetics, Univeristy of California Los Angeles, Los Angeles, CA USA; Division of Neuroscience, Oregon National Primate Research Center, Beaverton, OR USA; Genomics & Microbiology Research Laboratory, NC Museum of Natural Sciences, Raleigh, NC USA; Yerkes Nonhuman Primate Genomics Core, Yerkes National Primate Research Center, Atlanta, GA USA; Environmental Toxicology, California National Primate Research Center, Davis, CA USA; Physiology, Tulane National Primate Research Center, New Orleans, LO USA; Neuroscience, University of Pennsylvania, Philadelphia, PA USA; Anthropology, University of California Davis, Davis, CA USA; Psychiatry and Human Behavior, University of Mississippi Medical Center, Jackson, MS USA; Genetics, Wisconsin National Primate Research Center, Madison, WI USA; Epidemiology, Human Genetics & Environmental Sciences, ᅟ, ᅟ; Epidemiology and Disease Control Human Genetics Center, University of Texas Health Science Center, Houston, TX USA; The Jackson Laboratory for Genomic Medicine, Farmington, CT USA; Department of Statistics, University of Connecticut, Storrs, CT USA; National Cancer Institute, Rockville, USA; St. Jude Children’s Research Hospital, Memphis, USA; National Cancer Institute, NIH, DHHS, Rockville, USA; Changhua Christian Hospital, Changhua, Taiwan Province of China; Biochemistry, National University of Singapore, Singapore, Singapore; Proteomics, Faraby Eye Hospital, Tehran, Iran Islamic Republic Of; R & D, MIB Co., Tehran, Iran Islamic Republic Of; Proteomics, Faraby Eye Hospital, Tehran, Iran Islamic Republic Of; R & D, MIB Co., Tehran, Iran Islamic Republic Of; Department of Clinical Laboratory Sciences, College of Applied Medical Sciences, King Saud University, Riyadh, Saudi Arabia; Laboratory of Genomic and Personalized Medicine, Center for Life Sciences, National Laboratory Astana, Nazarbayev University, ᅟ, Kazakhstan; Cardiology, JSC “National Research Cardiac Surgery Center”, National medical holding, Astana, Kazakhstan; UAE Genetic Diseases Association, Dubai, United Arab Emirates; Research Center for Statistics, University of Geneva, Switzerland, Geneva Switzerland; Department of Statistics, University of South Carolina, Columbia, USA; Department of Statistics, University of Illinois at Urbana Champaign, Champaign, USA; Department of Molecular Genetics, Medical Genetics Center, Ain Shams University, Cairo, Egypt; Department of Medical Genetics, Umm Al-Qura University, ᅟ, Saudi Arabia; Department of Psychology, Umm Al-Qura University, Faculty of Education, Mecca, Saudi Arabia; Institute of Genetic Medicine, Johns Hopkins School of Medicine, Baltimore, USA; The Jackson Laboratory, Bar Harbor, USA; Graduate School of Biomedical Science and Engineering, University of Maine, Orono, USA; Department of Medical Genetics, Faculty of Medicine, Umm Al-Qura University, Makkah, Saudi Arabia; Department of Basic Sciences, College of Science and Health Professions, King Saud Bin Abdulaziz University for Health Sciences, Riyadh, Saudi Arabia; Department of Pediatric, King Faisal Specialist Hospital and Research Centre, Jeddah, Saudi Arabia; Department of Pediatric, Madinah Maternity and Children’s Hospital, Madinah, Saudi Arabia; Pediatric, King Fahad Medical City, Riyadh, Saudi Arabia; Molecular Diagnostics Unit, Department of Laboratory and Blood Bank, King Abdullah Medical City, Makkah, Saudi Arabia; Medical Genetics, King Salman Armed Forces Hospital, Tabuk, Saudi Arabia; Medical Genetics, King Fahad Medical City, Riyadh, Saudi Arabia; Center for Life Science Technologies, RIKEN Yokohama, Yokohama City, Kanagawa Japan; Brain Science Institute (BSI), Launey Research Unit, RIKEN Wako, Wako, Japan; Biology and Biochemistry, University of Houston, Houston, USA; McKusick-Nathans Institute of Genetic Medicine, Johns Hopkins University School of Medicine, Baltimore, USA; Technical University of Denmark, Center for Biological Sequence Analysis, Lyngby, Denmark; Molecular Biology, Netaji Subhas Chandra Bose Cancer Research Institute, Kolkata, India; Medical Oncology, Netaji Subhas Chandra Bose Cancer Research Institute, Kolkata, India; University of Houston-Downtown, Houston, USA; National Institute of Biomedical Genomics, Kalyani, India; Chittaranjan National Cancer Institute, Kolkata, India; Human Genetics, Faculty of Medicine, Alexandria, Egypt; Microbiology, Faculty of Medicine, Alexandria, Egypt; Clinical and Experimental Surgery, Faculty of Medicine, Alexandria, Egypt; Clinical and Experimental Internal Medicine, Medical Research Institute, Faculty of Medicine, Alexandria, Egypt; Clinical Oncology and Nuclear Medicine, Faculty of Medicine, Alexandria, Egypt; Genetics, Stanford University, Palo Alto, USA; Directed Genomics, ᅟ, USA; New England Biolabs, Ipswich, USA; Pathology, University of North Dakota, Grand Forks, USA; Oncogenomics, Inmegen, Mexico City, Mexico; Genetics, Stanford University, Palo Alto, USA; Stanford Cancer Institute, Stanford University, Palo Alto, USA; Urology, Stanford University, Palo Alto, USA; Cancer Genomic Laboratory, National Institute of Genomic Medicine (INMEGEN), ᅟ, Mexico; Biología, FES -Iztacala, UNAM, ᅟ, Mexico; Hospital de Pediatría, CMN SXXI, IMSS, ᅟ, Mexico; Investigación Médica en Inmunología, CMN La Raza, IMSS, ᅟ, Mexico; Diagnóstico Molecular H1N1-Influenza , UMAE-IMSS, Mérida, Yucatán Mexico; Hematología Pediátrica, CMN La Raza, IMSS, ᅟ, Mexico; Hemato-Oncología, Hospital Infantil de México, ᅟ, Mexico; Cancer Genomics Laboratory, INMEGEN, ᅟ, Mexico; Coordinación de Investigación en Salud, IMSS, ᅟ, Mexico; Molecular Immunology Department, Palladin Institute of Biochemistry of the National Academy of Sciences of Ukraine, Kiev, Ukraine; The Jackson Laboratory, Bar Harbor, USA; Department of Medical Genetics and Oncogene, Morocco; Medical Genetics, CHU Hassan II Fes, Fes, Morocco; Evolutionary Genetics, Institute of Medical Genetics, ᅟ, Russian Federation; Siberian State Medical University, Tomsk, Russian Federation; National Institutes of Health, University of the Philippines, Manila, Philippines; Philippine Genome Center, University of the Philippines, Quezon City, Philippines; College of Medicine, University of the Philippines, Manila, Philippines; Department of Molecular and Human Genetics, Baylor College of Medicine, Houston, TX United States; Philippine General Hospital, University of the Philippines, Manila, Philippines; The Jackson Laboratory, Bar Harbor, USA; Graduate School of Biomedical Sciences & Engineering, The University of Maine, Orono, USA; Center For Gene Therapy, The Research Institute at Nationwide Children’s Hospital, Columbus, Ohio USA; Laboratory of Genomic and Personalized Medicine, National Laboratory Astana, Nazarbayev University, ᅟ, Kazakhstan; Cardiology, JSC “National Research Cardiac Surgery Center”, National Medical Holding, Astana, Kazakhstan; Medical Laboratory Technology, ᅟ, Saudi Arabia; Center of Innovation in Personalized Medicine, King Abdulaziz University, Jeddah, Saudi Arabia; Advanced Molecular Diagnostics Laboratory, ᅟ, Canada; Princess Margaret Cancer Centre, Toronto, Canada; NCBI, NIH, Bethesda, USA; National Institutes of Health, Bethesda, USA; Computational Genomics, National Institute of Genomic Medicine, Mexico City, Mexico; Computer Science, Faculty of Computing and Information Technology, King AbdulAziz University, Rabigh, Saudi Arabia; Institute of Applied Cancer Science, ᅟ, USA; Genomic Medicine, University of Texas, ᅟ, ᅟ; MD Anderson Cancer Center, Houston, USA; Human Genome Sequencing Center, Baylor College of Medicine, ᅟ, USA; Human Genetics Center, University of Texas Health Science Center at Houston, Houston, USA; Center for Life Science Technologies, RIKEN, Yokohama, Japan; Preventive Medicine and Diagnosis Innovation Program, RIKEN, Wako, Japan; The Roslin Institute and Royal (Dick) School of Veterinary Studies, University of Edinburgh, Edinburgh, UK; Department of Biology & Biotech Research and Innovation Centre, University of Copenhagen, Copenhagen, Denmark; Advanced Center for Computing and Communication, RIKEN, Yokohama, Japan; Human Genome Sequencing Center, Baylor College of Medicine, Houston, Texas USA; Spiral Genetics, Seattle, Washington USA; Human Genetics Center and Department of Epidemiology, UT School of Public Health, Houston, Texas USA; Anatomy, Arabian Gulf University, Manama, Bahrain; Biochemistry, Arabian Gulf University, Manama, Bahrain; Central Laboratory, Ministry of Science and Technology, ᅟ, Sudan; College of Animal Production Science and Technology, Sudan University of Science and Technology, Khartoum, Sudan; Bioquimica y Medicina Molecular, Universidad Autónoma de Nuevo León, Monterrey, Mexico; Universidad Nacional Autónoma de México, ᅟ, Mexico; Instituto Nacional de Ciencias Médicas y Nutrición Salvador Zubirán, Distrito Federal, Mexico; Computational Genomics, Nacional de Medicina Genomica, Mexico City, Mexico; Cancer Genomics Laboratory, Nacional de Medicina Genomica, Mexico City, Mexico; Nutrigenetics and Nutrigenomics Laboratory, Instituto Nacional de Medicina Genomica, Mexico City, Mexico; Human Genetics Center, University of Texas Health Science Center, Houston, USA; System Genetics, The Jackson Laboratory, Bar Harbor, USA; Center of Innovation in Personalized Medicine, Faculty of Applied Medical Sciences, King Abdulaziz University, ᅟ, Saudi Arabia; Ob/Gyn, King Abdulaziz University Hospital, Jeddah, Saudi Arabia; The Jackson Laboratory for Genomic Medicine, Farmington, USA; Department of Mathematical Engineering, Istanbul Technical University, Istanbul, Turkey; Department of Biomedical Engineering, University of Connecticut, Storrs, USA; Directed Genomics, Ipswich, USA; New England Biolabs, Ipswich, USA; Genetics, Stanford University, Stanford, USA; DNA Diagnostics Center, Fairfield, USA; Watershed DNA, Crozet, USA; Vibrant Gene Consulting, Cambridge, USA; Agilent Technologies, Agilent Technologies, Waldbronn, Germany; Agilent Technologies UK Ltd, Agilent Technologies UK Ltd., Edinburgh, UK; NIH/NLM/NCBI, Bethesda, USA; Institute of Human Genetics, Department of Molecular Biology & Genomics, Centre for Health Sciences, ᅟ, Mexico; Rheumatoloty, Centre for Health Sciences, ᅟ, Mexico; Public Health, Centre for Health Sciences, ᅟ, Mexico; Biochemistry, Centre for Health Sciences, University of Guadalajara, Guadalajara, Mexico; Human Genetics Unit, Department Anthropology, Sri Venkateswara University, Tirupati, India; Department of Medical Genetics, Faculty of Medicine, Umm Al-Qura University, Makkah, Saudi Arabia; Department of Basic Sciences, College of Science and Health Professions, King Saud Bin Abdul Aziz University for Health Sciences, Riyadh, Saudi Arabia; Science and Technology Unit, Umm Al Qura University, Mecca, Saudi Arabia; Department of Medical Genetics, Umm Al Qura University, Mecca, Saudi Arabia; Medical Clinic 3 – Hematology/Oncology, University Hospital Regensburg, Regensburg, Germany; Department of Pathology, University Hospital Regensburg, Regensburg, Germany; IgNova GmbH, Oberursel, Germany; Department of Medical Genetics Faculty of Medicine, Umm Al Qura University, Mecca, Saudi Arabia; Department of Ansethesiologie, University of Würzburg Medical School, Würzburg, Germany; Department of microbiology, University Hospital Regensburg, Regensburg, Germany; Department of Medical Genetics, Faculty of Medicine, Umm Al-Qura University, ᅟ, Saudi Arabia; Science and Technology Unit, Umm Al-Qura University, ᅟ, Saudi Arabia; Molecular Diagnostics Unit, Department of Laboratory and Blood Bank, King Abdullah Medical City, Makkah, Saudi Arabia; King Faisal Specialist Hospital and Research Centre, ᅟ, Saudi Arabia; Faculty of Medicine, Alfaisal University, Riyadh, Saudi Arabia; Biology, National University of Pharmacy, Kharkiv, Ukraine; Pharmaceutical Marketing and Management, National University of Pharmacy, Kharkiv, Ukraine; Human Physiology and Anatomy, National University of Pharmacy, Kharkiv, Ukraine; Biology, National University of Pharmacy, Kharkiv, Ukraine; Pharmaceutical Marketing and Management, National University of Pharmacy, Kharkiv, Ukraine; Human Physiology and Anatomy, National University of Pharmacy, Kharkiv, Ukraine; Biology, National University of Pharmacy, Kharkiv, Ukraine; Pharmaceutical Marketing and Management, National University of Pharmacy, Kharkiv, Ukraine; Human Physiology and Anatomy, National University of Pharmacy, Kharkiv, Ukraine; Department of Medical Genetics Faculty of Medicine, Umm Al Qura University, Mecca, Saudi Arabia; Science and Technology Unit, Umm Al Qura University, Mecca, Saudi Arabia; Molecular Diagnostics Unit Department of Laboratory Medicine and Blood Bank, King Abdullah Medical City, ᅟ, Saudi Arabia; Pathology and Laboratory Medicine, King Faisal Specialist Hospital and Research Centre, Riyadh, Saudi Arabia

## Abstract

O1 The metabolomics approach to autism: identification of biomarkers for early detection of autism spectrum disorder

A. K. Srivastava, Y. Wang, R. Huang, C. Skinner, T. Thompson, L. Pollard, T. Wood, F. Luo, R. Stevenson

O2 Phenome-wide association study for smoking- and drinking-associated genes in 26,394 American women with African, Asian, European, and Hispanic descents

R. Polimanti, J. Gelernter

O3 Effects of prenatal environment, genotype and DNA methylation on birth weight and subsequent postnatal outcomes: findings from GUSTO, an Asian birth cohort

X. Lin, I. Y. Lim, Y. Wu, A. L. Teh, L. Chen, I. M. Aris, S. E. Soh, M. T. Tint, J. L. MacIsaac, F. Yap, K. Kwek, S. M. Saw, M. S. Kobor, M. J. Meaney, K. M. Godfrey, Y. S. Chong, J. D. Holbrook, Y. S. Lee, P. D. Gluckman, N. Karnani, GUSTO study group

O4 High-throughput identification of specific qt interval modulating enhancers at the SCN5A locus

A. Kapoor, D. Lee, A. Chakravarti

O5 Identification of extracellular matrix components inducing cancer cell migration in the supernatant of cultivated mesenchymal stem cells

C. Maercker, F. Graf, M. Boutros

O6 Single cell allele specific expression (ASE) IN T21 and common trisomies: a novel approach to understand DOWN syndrome and other aneuploidies

G. Stamoulis, F. Santoni, P. Makrythanasis, A. Letourneau, M. Guipponi, N. Panousis, M. Garieri, P. Ribaux, E. Falconnet, C. Borel, S. E. Antonarakis

O7 Role of microRNA in LCL to IPSC reprogramming

S. Kumar, J. Curran, J. Blangero

O8 Multiple enhancer variants disrupt gene regulatory network in Hirschsprung disease

S. Chatterjee, A. Kapoor, J. Akiyama, D. Auer, C. Berrios, L. Pennacchio, A. Chakravarti

O9 Metabolomic profiling for the diagnosis of neurometabolic disorders

T. R. Donti, G. Cappuccio, M. Miller, P. Atwal, A. Kennedy, A. Cardon, C. Bacino, L. Emrick, J. Hertecant, F. Baumer, B. Porter, M. Bainbridge, P. Bonnen, B. Graham, R. Sutton, Q. Sun, S. Elsea

O10 A novel causal methylation network approach to Alzheimer’s disease

Z. Hu, P. Wang, Y. Zhu, J. Zhao, M. Xiong, David A Bennett

O11 A microRNA signature identifies subtypes of triple-negative breast cancer and reveals MIR-342-3P as regulator of a lactate metabolic pathway

A. Hidalgo-Miranda, S. Romero-Cordoba, S. Rodriguez-Cuevas, R. Rebollar-Vega, E. Tagliabue, M. Iorio, E. D’Ippolito, S. Baroni

O12 Transcriptome analysis identifies genes, enhancer RNAs and repetitive elements that are recurrently deregulated across multiple cancer types

B. Kaczkowski, Y. Tanaka, H. Kawaji, A. Sandelin, R. Andersson, M. Itoh, T. Lassmann, the FANTOM5 consortium, Y. Hayashizaki, P. Carninci, A. R. R. Forrest

O13 Elevated mutation and widespread loss of constraint at regulatory and architectural binding sites across 11 tumour types

C. A. Semple

O14 Exome sequencing provides evidence of pathogenicity for genes implicated in colorectal cancer

E. A. Rosenthal, B. Shirts, L. Amendola, C. Gallego, M. Horike-Pyne, A. Burt, P. Robertson, P. Beyers, C. Nefcy, D. Veenstra, F. Hisama, R. Bennett, M. Dorschner, D. Nickerson, J. Smith, K. Patterson, D. Crosslin, R. Nassir, N. Zubair, T. Harrison, U. Peters, G. Jarvik, NHLBI GO Exome Sequencing Project

O15 The tandem duplicator phenotype as a distinct genomic configuration in cancer

F. Menghi, K. Inaki, X. Woo, P. Kumar, K. Grzeda, A. Malhotra, H. Kim, D. Ucar, P. Shreckengast, K. Karuturi, J. Keck, J. Chuang, E. T. Liu

O16 Modeling genetic interactions associated with molecular subtypes of breast cancer

B. Ji, A. Tyler, G. Ananda, G. Carter

O17 Recurrent somatic mutation in the MYC associated factor X in brain tumors

H. Nikbakht, M. Montagne, M. Zeinieh, A. Harutyunyan, M. Mcconechy, N. Jabado, P. Lavigne, J. Majewski

O18 Predictive biomarkers to metastatic pancreatic cancer treatment

J. B. Goldstein, M. Overman, G. Varadhachary, R. Shroff, R. Wolff, M. Javle, A. Futreal, D. Fogelman

O19 DDIT4 gene expression as a prognostic marker in several malignant tumors

L. Bravo, W. Fajardo, H. Gomez, C. Castaneda, C. Rolfo, J. A. Pinto

O20 Spatial organization of the genome and genomic alterations in human cancers

K. C. Akdemir, L. Chin, A. Futreal, ICGC PCAWG Structural Alterations Group

O21 Landscape of targeted therapies in solid tumors

S. Patterson, C. Statz, S. Mockus

O22 Genomic analysis reveals novel drivers and progression pathways in skin basal cell carcinoma

S. N. Nikolaev, X. I. Bonilla, L. Parmentier, B. King, F. Bezrukov, G. Kaya, V. Zoete, V. Seplyarskiy, H. Sharpe, T. McKee, A. Letourneau, P. Ribaux, K. Popadin, N. Basset-Seguin, R. Ben Chaabene, F. Santoni, M. Andrianova, M. Guipponi, M. Garieri, C. Verdan, K. Grosdemange, O. Sumara, M. Eilers, I. Aifantis, O. Michielin, F. de Sauvage, S. Antonarakis

O23 Identification of differential biomarkers of hepatocellular carcinoma and cholangiocarcinoma via transcriptome microarray meta-analysis

S. Likhitrattanapisal

O24 Clinical validity and actionability of multigene tests for hereditary cancers in a large multi-center study

S. Lincoln, A. Kurian, A. Desmond, S. Yang, Y. Kobayashi, J. Ford, L. Ellisen

O25 Correlation with tumor ploidy status is essential for correct determination of genome-wide copy number changes by SNP array

T. L. Peters, K. R. Alvarez, E. F. Hollingsworth, D. H. Lopez-Terrada

O26 Nanochannel based next-generation mapping for interrogation of clinically relevant structural variation

A. Hastie, Z. Dzakula, A. W. Pang, E. T. Lam, T. Anantharaman, M. Saghbini, H. Cao, BioNano Genomics

O27 Mutation spectrum in a pulmonary arterial hypertension (PAH) cohort and identification of associated truncating mutations in TBX4

C. Gonzaga-Jauregui, L. Ma, A. King, E. Berman Rosenzweig, U. Krishnan, J. G. Reid, J. D. Overton, F. Dewey, W. K. Chung

O28 NORTH CAROLINA macular dystrophy (MCDR1): mutations found affecting PRDM13

K. Small, A. DeLuca, F. Cremers, R. A. Lewis, V. Puech, B. Bakall, R. Silva-Garcia, K. Rohrschneider, M. Leys, F. S. Shaya, E. Stone

O29 PhenoDB and genematcher, solving unsolved whole exome sequencing data

N. L. Sobreira, F. Schiettecatte, H. Ling, E. Pugh, D. Witmer, K. Hetrick, P. Zhang, K. Doheny, D. Valle, A. Hamosh

O30 Baylor-Johns Hopkins Center for Mendelian genomics: a four year review

S. N. Jhangiani, Z. Coban Akdemir, M. N. Bainbridge, W. Charng, W. Wiszniewski, T. Gambin, E. Karaca, Y. Bayram, M. K. Eldomery, J. Posey, H. Doddapaneni, J. Hu, V. R. Sutton, D. M. Muzny, E. A. Boerwinkle, D. Valle, J. R. Lupski, R. A. Gibbs

O31 Using read overlap assembly to accurately identify structural genetic differences in an ashkenazi jewish trio

S. Shekar, W. Salerno, A. English, A. Mangubat, J. Bruestle

O32 Legal interoperability: a sine qua non for international data sharing

A. Thorogood, B. M. Knoppers, Global Alliance for Genomics and Health - Regulatory and Ethics Working Group

O33 High throughput screening platform of competent sineups: that can enhance translation activities of therapeutic target

H. Takahashi, K. R. Nitta, A. Kozhuharova, A. M. Suzuki, H. Sharma, D. Cotella, C. Santoro, S. Zucchelli, S. Gustincich, P. Carninci

O34 The undiagnosed diseases network international (UDNI): clinical and laboratory research to meet patient needs

J. J. Mulvihill, G. Baynam, W. Gahl, S. C. Groft, K. Kosaki, P. Lasko, B. Melegh, D. Taruscio

O36 Performance of computational algorithms in pathogenicity predictions for activating variants in oncogenes versus loss of function mutations in tumor suppressor genes

R. Ghosh, S. Plon

O37 Identification and electronic health record incorporation of clinically actionable pharmacogenomic variants using prospective targeted sequencing

S. Scherer, X. Qin, R. Sanghvi, K. Walker, T. Chiang, D. Muzny, L. Wang, J. Black, E. Boerwinkle, R. Weinshilboum, R. Gibbs

O38 Melanoma reprogramming state correlates with response to CTLA-4 blockade in metastatic melanoma

T. Karpinets, T. Calderone, K. Wani, X. Yu, C. Creasy, C. Haymaker, M. Forget, V. Nanda, J. Roszik, J. Wargo, L. Haydu, X. Song, A. Lazar, J. Gershenwald, M. Davies, C. Bernatchez, J. Zhang, A. Futreal, S. Woodman

O39 Data-driven refinement of complex disease classification from integration of heterogeneous functional genomics data in GeneWeaver

E. J. Chesler, T. Reynolds, J. A. Bubier, C. Phillips, M. A. Langston, E. J. Baker

O40 A general statistic framework for genome-based disease risk prediction

M. Xiong, L. Ma, N. Lin, C. Amos

O41 Integrative large-scale causal network analysis of imaging and genomic data and its application in schizophrenia studies

N. Lin, P. Wang, Y. Zhu, J. Zhao, V. Calhoun, M. Xiong

O42 Big data and NGS data analysis: the cloud to the rescue

O. Dobretsberger, M. Egger, F. Leimgruber

O43 Cpipe: a convergent clinical exome pipeline specialised for targeted sequencing

S. Sadedin, A. Oshlack, Melbourne Genomics Health Alliance

O44 A Bayesian classification of biomedical images using feature extraction from deep neural networks implemented on lung cancer data

V. A. A. Antonio, N. Ono, Clark Kendrick C. Go

O45 MAV-SEQ: an interactive platform for the Management, Analysis, and Visualization of sequence data

Z. Ahmed, M. Bolisetty, S. Zeeshan, E. Anguiano, D. Ucar

O47 Allele specific enhancer in EPAS1 intronic regions may contribute to high altitude adaptation of Tibetans

C. Zeng, J. Shao

O48 Nanochannel based next-generation mapping for structural variation detection and comparison in trios and populations

H. Cao, A. Hastie, A. W. Pang, E. T. Lam, T. Liang, K. Pham, M. Saghbini, Z. Dzakula

O49 Archaic introgression in indigenous populations of Malaysia revealed by whole genome sequencing

Y. Chee-Wei, L. Dongsheng, W. Lai-Ping, D. Lian, R. O. Twee Hee, Y. Yunus, F. Aghakhanian, S. S. Mokhtar, C. V. Lok-Yung, J. Bhak, M. Phipps, X. Shuhua, T. Yik-Ying, V. Kumar, H. Boon-Peng

O50 Breast and ovarian cancer prevention: is it time for population-based mutation screening of high risk genes?

I. Campbell, M.-A. Young, P. James, Lifepool

O53 Comprehensive coverage from low DNA input using novel NGS library preparation methods for WGS and WGBS

C. Schumacher, S. Sandhu, T. Harkins, V. Makarov

O54 Methods for large scale construction of robust PCR-free libraries for sequencing on Illumina HiSeqX platform

H. DoddapaneniR. Glenn, Z. Momin, B. Dilrukshi, H. Chao, Q. Meng, B. Gudenkauf, R. Kshitij, J. Jayaseelan, C. Nessner, S. Lee, K. Blankenberg, L. Lewis, J. Hu, Y. Han, H. Dinh, S. Jireh, K. Walker, E. Boerwinkle, D. Muzny, R. Gibbs

O55 Rapid capture methods for clinical sequencing

J. Hu, K. Walker, C. Buhay, X. Liu, Q. Wang, R. Sanghvi, H. Doddapaneni, Y. Ding, N. Veeraraghavan, Y. Yang, E. Boerwinkle, A. L. Beaudet, C. M. Eng, D. M. Muzny, R. A. Gibbs

O56 A diploid personal human genome model for better genomes from diverse sequence data

K. C. C. Worley, Y. Liu, D. S. T. Hughes, S. C. Murali, R. A. Harris, A. C. English, X. Qin, O. A. Hampton, P. Larsen, C. Beck, Y. Han, M. Wang, H. Doddapaneni, C. L. Kovar, W. J. Salerno, A. Yoder, S. Richards, J. Rogers, J. R. Lupski, D. M. Muzny, R. A. Gibbs

O57 Development of PacBio long range capture for detection of pathogenic structural variants

Q. Meng, M. Bainbridge, M. Wang, H. Doddapaneni, Y. Han, D. Muzny, R. Gibbs

O58 Rhesus macaques exhibit more non-synonymous variation but greater impact of purifying selection than humans

R. A. Harris, M. Raveenedran, C. Xue, M. Dahdouli, L. Cox, G. Fan, B. Ferguson, J. Hovarth, Z. Johnson, S. Kanthaswamy, M. Kubisch, M. Platt, D. Smith, E. Vallender, R. Wiseman, X. Liu, J. Below, D. Muzny, R. Gibbs, F. Yu, J. Rogers

O59 Assessing RNA structure disruption induced by single-nucleotide variation

J. Lin, Y. Zhang, Z. Ouyang

P1 A meta-analysis of genome-wide association studies of mitochondrial dna copy number

A. Moore, Z. Wang, J. Hofmann, M. Purdue, R. Stolzenberg-Solomon, S. Weinstein, D. Albanes, C.-S. Liu, W.-L. Cheng, T.-T. Lin, Q. Lan, N. Rothman, S. Berndt

P2 Missense polymorphic genetic combinations underlying down syndrome susceptibility

E. S. Chen

P4 The evaluation of alteration of ELAM-1 expression in the endometriosis patients

H. Bahrami, A. Khoshzaban, S. Heidari Keshal

P5 Obesity and the incidence of apolipoprotein E polymorphisms in an assorted population from Saudi Arabia population

K. K. R. Alharbi

P6 Genome-associated personalized antithrombotical therapy for patients with high risk of thrombosis and bleeding

M. Zhalbinova, A. Akilzhanova, S. Rakhimova, M. Bekbosynova, S. Myrzakhmetova

P7 Frequency of Xmn1 polymorphism among sickle cell carrier cases in UAE population

M. Matar

P8 Differentiating inflammatory bowel diseases by using genomic data: dimension of the problem and network organization

N. Mili, R. Molinari, Y. Ma, S. Guerrier

P9 Vulnerability of genetic variants to the risk of autism among Saudi children

N. Elhawary, M. Tayeb, N. Bogari, N. Qotb

P10 Chromatin profiles from ex vivo purified dopaminergic neurons establish a promising model to support studies of neurological function and dysfunction

S. A. McClymont, P. W. Hook, L. A. Goff, A. McCallion

P11 Utilization of a sensitized chemical mutagenesis screen to identify genetic modifiers of retinal dysplasia in homozygous Nr2e3^rd7^ mice

Y. Kong, J. R. Charette, W. L. Hicks, J. K. Naggert, L. Zhao, P. M. Nishina

P12 Ion torrent next generation sequencing of recessive polycystic kidney disease in Saudi patients

B. M. Edrees, M. Athar, F. A. Al-Allaf, M. M. Taher, W. Khan, A. Bouazzaoui, N. A. Harbi, R. Safar, H. Al-Edressi, A. Anazi, N. Altayeb, M. A. Ahmed, K. Alansary, Z. Abduljaleel

P13 Digital expression profiling of Purkinje neurons and dendrites in different subcellular compartments

A. Kratz, P. Beguin, S. Poulain, M. Kaneko, C. Takahiko, A. Matsunaga, S. Kato, A. M. Suzuki, N. Bertin, T. Lassmann, R. Vigot, P. Carninci, C. Plessy, T. Launey

P14 The evolution of imperfection and imperfection of evolution: the functional and functionless fractions of the human genome

D. Graur

P16 Species-independent identification of known and novel recurrent genomic entities in multiple cancer patients

J. Friis-Nielsen, J. M. Izarzugaza, S. Brunak

P18 Discovery of active gene modules which are densely conserved across multiple cancer types reveal their prognostic power and mutually exclusive mutation patterns

B. S. Soibam

P19 Whole exome sequencing of dysplastic leukoplakia tissue indicates sequential accumulation of somatic mutations from oral precancer to cancer

D. Das, N. Biswas, S. Das, S. Sarkar, A. Maitra, C. Panda, P. Majumder

P21 Epigenetic mechanisms of carcinogensis by hereditary breast cancer genes

J. J. Gruber, N. Jaeger, M. Snyder

P22 RNA direct: a novel RNA enrichment strategy applied to transcripts associated with solid tumors

K. Patel, S. Bowman, T. Davis, D. Kraushaar, A. Emerman, S. Russello, N. Henig, C. Hendrickson

P23 RNA sequencing identifies gene mutations for neuroblastoma

K. Zhang

P24 Participation of SFRP1 in the modulation of TMPRSS2-ERG fusion gene in prostate cancer cell lines

M. Rodriguez-Dorantes, C. D. Cruz-Hernandez, C. D. P. Garcia-Tobilla, S. Solorzano-Rosales

P25 Targeted Methylation Sequencing of Prostate Cancer

N. Jäger, J. Chen, R. Haile, M. Hitchins, J. D. Brooks, M. Snyder

P26 Mutant TPMT alleles in children with acute lymphoblastic leukemia from México City and Yucatán, Mexico

S. Jiménez-Morales, M. Ramírez, J. Nuñez, V. Bekker, Y. Leal, E. Jiménez, A. Medina, A. Hidalgo, J. Mejía

P28 Genetic modifiers of Alström syndrome

J. Naggert, G. B. Collin, K. DeMauro, R. Hanusek, P. M. Nishina

P31 Association of genomic variants with the occurrence of angiotensin-converting-enzyme inhibitor (ACEI)-induced coughing among Filipinos

E. M. Cutiongco De La Paz, R. Sy, J. Nevado, P. Reganit, L. Santos, J. D. Magno, F. E. Punzalan , D. Ona , E. Llanes, R. L. Santos-Cortes , R. Tiongco, J. Aherrera, L. Abrahan, P. Pagauitan-Alan; Philippine Cardiogenomics Study Group

P32 The use of “humanized” mouse models to validate disease association of a de novo GARS variant and to test a novel gene therapy strategy for Charcot-Marie-Tooth disease type 2D

K. H. Morelli, J. S. Domire, N. Pyne, S. Harper, R. Burgess

P34 Molecular regulation of chondrogenic human induced pluripotent stem cells

M. A. Gari, A. Dallol, H. Alsehli, A. Gari, M. Gari, A. Abuzenadah

P35 Molecular profiling of hematologic malignancies: implementation of a variant assessment algorithm for next generation sequencing data analysis and clinical reporting

M. Thomas, M. Sukhai, S. Garg, M. Misyura, T. Zhang, A. Schuh, T. Stockley, S. Kamel-Reid

P36 Accessing genomic evidence for clinical variants at NCBI

S. Sherry, C. Xiao, D. Slotta, K. Rodarmer, M. Feolo, M. Kimelman, G. Godynskiy, C. O’Sullivan, E. Yaschenko

P37 NGS-SWIFT: a cloud-based variant analysis framework using control-accessed sequencing data from DBGAP/SRA

C. Xiao, E. Yaschenko, S. Sherry

P38 Computational assessment of drug induced hepatotoxicity through gene expression profiling

C. Rangel-Escareño, H. Rueda-Zarate

P40 Flowr: robust and efficient pipelines using a simple language-agnostic approach;ultraseq; fast modular pipeline for somatic variation calling using flowr

S. Seth, S. Amin, X. Song, X. Mao, H. Sun, R. G. Verhaak, A. Futreal, J. Zhang

P41 Applying “Big data” technologies to the rapid analysis of heterogenous large cohort data

S. J. Whiite, T. Chiang, A. English, J. Farek, Z. Kahn, W. Salerno, N. Veeraraghavan, E. Boerwinkle, R. Gibbs

P42 FANTOM5 web resource for the large-scale genome-wide transcription start site activity profiles of wide-range of mammalian cells

T. Kasukawa, M. Lizio, J. Harshbarger, S. Hisashi, J. Severin, A. Imad, S. Sahin, T. C. Freeman, K. Baillie, A. Sandelin, P. Carninci, A. R. R. Forrest, H. Kawaji, The FANTOM Consortium

P43 Rapid and scalable typing of structural variants for disease cohorts

W. Salerno, A. English, S. N. Shekar, A. Mangubat, J. Bruestle, E. Boerwinkle, R. A. Gibbs

P44 Polymorphism of glutathione S-transferases and sulphotransferases genes in an Arab population

A. H. Salem, M. Ali, A. Ibrahim, M. Ibrahim

P46 Genetic divergence of CYP3A5*3 pharmacogenomic marker for native and admixed Mexican populations

J. C. Fernandez-Lopez, V. Bonifaz-Peña, C. Rangel-Escareño, A. Hidalgo-Miranda, A. V. Contreras

P47 Whole exome sequence meta-analysis of 13 white blood cell, red blood cell, and platelet traits

L. Polfus, CHARGE and NHLBI Exome Sequence Project Working Groups

P48 Association of adipoq gene with type 2 diabetes and related phenotypes in african american men and women: The jackson heart study

S. Davis, R. Xu, S. Gebeab, P Riestra, A Gaye, R. Khan, J. Wilson, A. Bidulescu

P49 Common variants in casr gene are associated with serum calcium levels in koreans

S. H. Jung, N. Vinayagamoorthy, S. H. Yim, Y. J. Chung

P50 Inference of multiple-wave population admixture by modeling decay of linkage disequilibrium with multiple exponential functions

Y. Zhou, S. Xu

P51 A Bayesian framework for generalized linear mixed models in genome-wide association studies

X. Wang, V. Philip, G. Carter

P52 Targeted sequencing approach for the identification of the genetic causes of hereditary hearing impairment

A. A. Abuzenadah, M. Gari, R. Turki, A. Dallol

P53 Identification of enhancer sequences by ATAC-seq open chromatin profiling

A. Uyar, A. Kaygun, S. Zaman, E. Marquez, J. George, D. Ucar

P54 Direct enrichment for the rapid preparation of targeted NGS libraries

C. L. Hendrickson, A. Emerman, D. Kraushaar, S. Bowman, N. Henig, T. Davis, S. Russello, K. Patel

P56 Performance of the Agilent D5000 and High Sensitivity D5000 ScreenTape assays for the Agilent 4200 Tapestation System

R. Nitsche, L. Prieto-Lafuente

P57 ClinVar: a multi-source archive for variant interpretation

M. Landrum, J. Lee, W. Rubinstein, D. Maglott

P59 Association of functional variants and protein physical interactions of human MUTY homolog linked with familial adenomatous polyposis and colorectal cancer syndrome

Z. Abduljaleel, W. Khan, F. A. Al-Allaf, M. Athar , M. M. Taher, N. Shahzad

P60 Modification of the microbiom constitution in the gut using chicken IgY antibodies resulted in a reduction of acute graft-versus-host disease after experimental bone marrow transplantation

A. Bouazzaoui, E. Huber, A. Dan, F. A. Al-Allaf, W. Herr, G. Sprotte, J. Köstler, A. Hiergeist, A. Gessner, R. Andreesen, E. Holler

P61 Compound heterozygous mutation in the *LDLR* gene in Saudi patients suffering severe hypercholesterolemia

F. Al-Allaf, A. Alashwal, Z. Abduljaleel, M. Taher, A. Bouazzaoui, H. Abalkhail, A. Al-Allaf, R. Bamardadh, M. Athar

## O1 The metabolomics approach to autism: identification of biomarkers for early detection of autism spectrum disorder

### A. K. Srivastava^1^, Y. Wang^2^, R. Huang^3^, C. Skinner^1^, T. Thompson^3^, L. Pollard^3^, T. Wood^3^, F. Luo^2^, R. Stevenson^1^

#### ^1^JCSRI, Greenwood Genetic Center, Greenwood, SC, USA; ^2^School of Computing, Clemson University, Clemson, SC, USA; ^3^Biochemical Genetics Laboratory, Greenwood Genetic Center, Greenwood, SC, USA

##### **Correspondence:** A. K. Srivastava – JCSRI, Greenwood Genetic Center, Greenwood, SC, USA

**Objectives**

From the first description by Leo Kanner [1], autism has been an enigmatic neurobehavioral phenomenon. The new genetic/genomic technologies of the past decade have not been as productive as originally anticipated in unveiling the mysteries of autism. The specific etiology of the majority of cases of autism spectrum disorder (ASD) is unknown, although numerous genetic/genomic variants and alterations of diverse cellular functions have been reported. Prompted by this failure, we have investigated whether the metabolomics approach might yield results which could simultaneously lead to a blood-based screening/diagnostic test and to treatment options.

**Methods**

Plasma samples from a clinically well-defined cohort of 100 male individuals, ages 2-16+ years, with ASD and 32 age-matched typically developing (TD) controls were subjected to global metabolomic analysis.

**Results**

We have identified more than 25 plasma metabolites among the approximately 650 metabolites analyzed, representing over 70 biochemical pathways, that can discriminate children with ASD as young as 2 years from children that are developing typically. The discriminating power was greatest in the 2–10 year age group and weaker in older age groups. The initial findings were validated in a second cohort of 83 children, males and females, ages 2–10 years, with ASD and 76 age and gender-matched TD children. The discriminant metabolites were associated with several key biochemical pathways suggestive of potential contributions of increased oxidative stress, mitochondrial dysfunction, inflammation and immune dysregulation in ASD. Further, targeted quantitative analysis of a subset of discriminating metabolites using tandem mass spectrometry provided a reliable laboratory method to detect children with ASD.

**Conclusion**

Metabolic profiling appears to be a robust technique to identify children with ASD ages 2–10 years and provides insights into the altered metabolic pathways in ASD, which could lead to treatment strategies.

**References**

1. Kanner, L. Autistic disturbances of affective contact. Nervous Child. 1943; 2: 217–250.

**Disclosure of interest**

None declared.

## O2 Phenome-wide association study for smoking- and drinking-associated genes in 26,394 American women with African, Asian, European, and Hispanic descents

### R. Polimanti^1^, J. Gelernter^1,2,3^

#### ^1^Department Psychiatry, Yale Sch Med and VA CT Healthcare Center, West Haven, CT, USA; ^2^Department Genetics, Yale Sch Med and VA CT Healthcare Center, West Haven, CT, USA; ^3^Department Neurobiology, Yale Sch Med and VA CT Healthcare Center, West Haven, CT, USA

##### **Correspondence:** R. Polimanti – Dept Psychiatry, Yale Sch Med and VA CT Healthcare Center, West Haven, CT, USA

**Objectives**

To uncover novel traits associated with nicotine and alcohol use genetics, we performed a phenome-wide association study in a large multi-ethnic cohort.

**Methods**

We investigated 7,688 African-Americans (AFR), 1,133 Asian-Americans (ASN), 14,081 European-Americans (EUR), and 3,492 Hispanic-Americans (HISP) from the Women’s Health Initiative, analyzing risk alleles located in the *CHRNA5*–*CHRNA3* locus (rs8034191, rs1051730, rs12914385, rs2036527, and rs16969968) for nicotine-related traits and *ADH1B* (rs1229984 and rs2066702) and *ALDH2* (rs671) for alcohol-related traits with respect to anthropometric characteristics, dietary habits, social status, psychological circumstances, reproductive history, health conditions, and nicotine- and alcohol-related traits.

**Results**

The investigated loci resulted associated with novel traits: rs1229984 were associated with family income (p=4.1*10^−12^), having a pet (p=6.5*10^−11^), partner education (p=1.8*10^−10^), “usually expect the best” (p=2.4*10^−7^), “felt calm and peaceful” (p=2.6*10^−7^), education (p=3.7*10^−6^), and number of term pregnancies (p=1.12*10^−5^) in EUR; rs1051730 and rs16969968 showed a suggestive association with “High cholesterol requiring pills ever” (p=3.8*10^−4^ and p=1.8*10^−4^) in trans-ethnic meta-analysis. We also replicated the known associations: rs80341911 was associated with cigarettes per day (CIGSDAY, p=3.4*10^−8^), smoking status (p=6.7*10^−3^), and “smoked at least 100 cigarettes” (p=7.3*10^−3^) in EUR; rs1051730 and rs16969968 were associated with CIGSDAY (p=9.1*10^−8^ and p=1.1*10^−7^) and lung cancer (p=7.3*10^−3^ and p=9.9*10^−3^) in EUR; rs2036527 was associated with CIGSDAY (p=3.5*10^−3^) in AFR; rs1229984 showed associations for alcohol servings (ALC, p=2.9*10^−6^), beer servings (p=3*10^−6^), wine servings (WINE, p=3.9*10^−6^), liquor servings (p=5.5*10^−6^), dietary alcohol (DIETALC, p=6.1*10^−6^), “Drinks alcohol (age 50)” (p=9.3*10^−6^) in EUR and for ALC (p=5.2*10^−5^) and DIETALC (p=9.6*10^−5^) in HISP; rs671 resulted associated with alcohol intake (p=3.8*10^−8^), DIETALC (p=1.9*10^−7^), ALC (p=1.3*10^−6^), WINE (p=1.1*10^−5^) and “Drank 12 alcoholic beverages ever” (p=1.2*10^−5^) in ASN.

**Conclusion**

We provided novel genetic data regarding the consequences of smoking and drinking behaviors and confirmed ethnic differences in their genetic predisposition.

**Disclosure of interest**

None declared.

## O3 Effects of prenatal environment, genotype and DNA methylation on birth weight and subsequent postnatal outcomes: findings from GUSTO, an Asian birth cohort

### X. Lin^1^, I. Y. Lim^1^, Y. Wu^1^, A. L. Teh^1^, L. Chen^1^, I. M. Aris^1^, S. E. Soh^1^, M. T. Tint^2^, J. L. MacIsaac^3^, F. Yap^4^, K. Kwek^4^, S. M. Saw^2^, M. S. Kobor^3^, M. J. Meaney^1^, K. M. Godfrey^5^, Y. S. Chong^1^, J. D. Holbrook^1^, Y. S. Lee^1^, P. D. Gluckman^1,6^, N. Karnani^1^, GUSTO study group

#### ^1^Singapore Institute for Clinical Sciences, Singapore, Singapore; ^2^National University of Singapore, Singapore, Singapore; ^3^University of British Columbia, Vancouver, British Columbia, Canada; ^4^KK Women’s and Children’s Hospital, Singapore, Singapore; ^5^University of Southampton and University Hospital Southampton NHS Foundation Trust, Southampton, UK; ^6^University of Auckland, Auckland, New Zealand

##### **Correspondence:** X. Lin – Singapore Institute for Clinical Sciences, Singapore, Singapore

**Objectives**

Prenatal environment and genetic polymorphism can have a lasting impact on offspring’s metabolic function by perturbing its epigenome. Birth weight is often used as a surrogate for the overall quality of the intrauterine environment. We present the first neonate epigenome-wide association study in an Asian mother-offspring cohort, that interrogates the effects of prenatal environment variables, umbilical cord DNA methylation and SNPs, on birth weight.

**Methods**

In GUSTO, a prospective mother-offspring cohort study (N=987), we examined the associations between DNA methylation, SNPs, birth weight and 11 prenatal environment variables. First, we investigated the association between perinatal methylome and birth weight to identify sites of variability in methylation. Second, we interrogated the contribution of genetic and prenatal environmental factors on this variability in the epigenome. Finally, we examined whether these methylation marks at birth were associated with offspring size and adiposity in early childhood.

**Results**

Methylation levels at 50 CpGs were significantly associated with birth weight, and a subset of these CpGs was located in genes and miRNA known to be involved in metabolic pathways/disorders. We further examined the influence of environmental and genetic factors on methylation at these 50 CpG sites. Sixteen CpGs were associated with both, an additional 24 CpGs were associated with only environmental factors, while only 3 CpGs were associated with genetic factors alone. Environmental factors associated with methylation were predominantly maternal-adiposity-related (pre-pregnancy body mass index, pregnancy weight gain and maternal glucose levels). Methylation levels at half of these CpGs were also associated with offspring size and adiposity in early childhood.

**Conclusion**

Developmental pathways to obesity begin before birth and involve genetic, epigenetic and environmental factors.

**Disclosure of interest**

X. Lin: None declared., I. Y. Lim: None declared., Y. Wu: None declared., A. L. Teh: None declared., L. Chen: None declared., I. M. Aris: None declared., S. E. Soh: None declared., M. T. Tint: None declared., J. L. MacIsaac: None declared., F. Yap: None declared., K. Kwek: None declared., S. M. Saw: None declared., M. S. Kobor: None declared., M. J. Meaney: None declared., K. M. Godfrey Conflict with: KMG has received reimbursement for speaking at conferences sponsored by companies selling nutritional products. He is part of an academic consortium that has received research funding from Abbott Nutrition, Nestec and Danone, Y. S. Chong Conflict with: YSC has received reimbursement for speaking at conferences sponsored by companies selling nutritional products. He is part of an academic consortium that has received research funding from Abbott Nutrition, Nestec and Danone, J. D. Holbrook: None declared., Y. S. Lee: None declared., P. D. Gluckman: None declared., N. Karnani: None declared.

## O4 High-throughput identification of specific qt interval modulating enhancers at the SCN5A locus

### A. Kapoor, D. Lee, A. Chakravarti

#### McKusick-Nathans Institute of Genetic Medicine, Johns Hopkins University School of Medicine, Baltimore, MD, USA

##### **Correspondence:** A. Kapoor – McKusick-Nathans Institute of Genetic Medicine, Johns Hopkins University School of Medicine, Baltimore, MD, USA

**Objectives**

Genome-wide association studies (GWAS) have indicated that sequence variation in cis-regulatory elements (CRE) plays important roles in common disease risk/trait variation, but identification of these causal variants has remained a major challenge in complex trait genetics. Here, we performed reporter assays for all common variants at the QT interval associated *SCN5A* GWAS locus, with the goal of identifying the underlying causal variants.

**Methods**

A target region of ~500kb at *SCN5A* was defined based on recombination hotspots (rate>10cM/Mb; HapMap) flanking the 5 independent QT interval GWAS hits. Within the target region, all common variants (minor allele frequency >5%) from the 1000 Genomes European ancestry populations in moderate linkage disequilibrium (r^2^>0.3) with any of the 5 GWAS hits were selected. Both alleles of these variants were amplified with flanking sequences and cloned upstream of a minimal promoter driven firefly luciferase gene in pGL4.23. Human cardiomyocyte cells, AC16, were transfected with test constructs and Renilla luciferase vector (for transfection normalization) in triplicate and luciferase assays were performed 24h later. Reporter assays on a subset of variants were repeated for assessing allelic difference in regulatory activity. All cloning and reporter assays were performed in 96- and 24-well plates.

**Results**

Of a total 121 variants selected, 112 variants in 104 amplicons passed primer design (amplicon size 256-617bp; median 397bp), and we successfully cloned both alleles for 106 variants in 98 amplicons. In reporter assays, compared to empty vector, 24 and 40 amplicons showed enhancer (>2-fold) and suppressor (<0.5-fold) activities in AC16 cells, respectively. Of these only 4 were observed as open chromatin regions in heart tissue in NIH Epigenomics data. Overall, 12 variants showed nominally significant allelic difference (P<0.05) in reporter activity and were repeated with 18 replicates and 7 variants were identified to have repeated significant allelic difference in regulatory activity.

**Conclusion**

Independent of the available epigenomic data, which are of limited relevance, an unbiased *in vitro* reporter screen for CREs overlapping all common variants associated with QT interval at the *SCN5A* GWAS locus identified 7 common cis-regulatory variants. Our immediate next goals are to a) evaluate the effect of deleting these 7 CREs on *SCN5A* expression in AC16 cells and b) identify the trans-acting factors regulating their functions.

**Disclosure of interest**

None declared.

## O5 Identification of extracellular matrix components inducing cancer cell migration in the supernatant of cultivated mesenchymal stem cells

### C. Maercker^1^, F. Graf^2^, M. Boutros^2^

#### ^1^Esslingen University of Applied Sciences, Esslingen, Germany; ^2^German Cancer Research Center, Heidelberg, Germany

##### **Correspondence:** C. Maercker – Esslingen University of Applied Sciences, Esslingen, Germany

**Objectives**

Some cancers show a strong tendency to metastasize to bone, a tissue of mesenchymal origin and a prominent site of mesenchymal stem cells (MSC) residing in the stem cell niche. With bone metastasis formation being one of the most detrimental steps in cancer progression, a better understanding of how bone metastases are initially formed is key to successfully targeting bone metastasis of, for example, prostate cancer. Recent reports have suggested that bone-metastasizing cancers may mimic the process of homing of hematopoietic stem cells to their bone niche.

**Methods**

In order to understand the role of MSC in metastasis formation, we investigated the interaction of primary human bone marrow MSC with established cancer cell lines able to metastasize to bone. With a trans-well migration assay we could show that MSC induced a rapid migration response of prostate and breast cancer cell lines already within two hours after start of the experiment. In order to identify factors stimulating cancer cell migration, MSC cell culture supernatant was separated by size exclusion and ion exchange chromatography. Migratory fractions then were further analyzed by mass spectrometry and antibody array analysis.

**Results**

With this approach we identified the extracellular matrix proteins type I and type III collagen, fibronectin and laminin 421 as potential drivers of cancer cell migration, which was confirmed by using recombinant proteins. RNAi experiments showed that the cancer cell extracellular matrix receptor beta 1 integrin obviously plays a pivotal role for cell migration.

**Conclusion**

From our results we conclude that the extracellular matrix as it is produced by MSC obviously plays a crucial role for cancer metastasis and therefore might be a promising anti-cancer drug target.

**Disclosure of interest**

None declared.

## O6 Single cell allele specific expression (ASE) IN T21 and common trisomies: a novel approach to understand DOWN syndrome and other aneuploidies

### G. Stamoulis^1^, F. Santoni^2^, P. Makrythanasis^2^, A. Letourneau^1^, M. Guipponi^2^, N. Panousis^1^, M. Garieri^1^, P. Ribaux^1^, E. Falconnet^1^, C. Borel^1^, S. E. Antonarakis^1,2,3^

#### ^1^Department of Genetic Medicine and Development, University of Geneva Medical School, Geneva, Switzerland; ^2^Geneva University Hospitals-HUG, Service of Genetic Medicine, Geneva, Switzerland; ^3^iGE3 Institute of Genetics and Genomics of Geneva, University of Geneva Medical School, Geneva, Switzerland

##### **Correspondence:** G. Stamoulis – Department of Genetic Medicine and Development, University of Geneva Medical School, Geneva, Switzerland

**Objectives**

Trisomy 21 is a model disorder of altered gene expression. We have previously used a pair of monozygotic twins discordant for trisomy 21 to study the global dysregulation of gene expression, without the noise due to genetic variation among individuals (*Nature:*508; 345–350;2014). The majority of previous studies focused on aneuploidies were conducted οn cell populations or tissues. Our study focusing on gene and allelic expression behaviour of single cells (SC), aims to reveal biological insights regarding the cellular impact of aneuploidy and uncover the mechanisms of gene dosage.

**Methods**

We estimated the allele specific expression (ASE) from RNAseq of ~1000 single cells in different aneuploidies. We used 352 SC fibroblasts (173 Normal and 179 T21 cells) from the pair of monozygotic twins discordant for T21, 166 SC from a mosaic T21, 176 SC from a mosaic T18, 151 SC from a mosaic T8, and 146 SC from a mosaic T13.

**Results**

In the monozygotic twins, a considerable number of heterozygous sites at the non-chr21 genome showed monoallelic expression (MAE);(Normal: 73.5 % monoallelic in 564,668 observations, and T21: 78.7 % monoallelic in 549,799 observations). There was also considerable MAE for chr21 sites in Normal and, surprisingly, in T21 cells as well (Normal: 63,3 % monoallelic in 5,009 observations, and T21: 72.8 % monoallelic in 6,456 observations). We classified the genes on chr21 in 3 classes according to the level of the aggregate MAE of their corresponding sites (9 monoallelic, 29 intermediate, 2 biallelic). Similar results, i.e. extensive MAE on the supernumerary chromosome genes, were also observed in the other aneuploidies.

**Conclusion**

We hypothesize that each class of genes contributes in a specific way to the phenotypic variability of Down Syndrome. Our analysis showed that, for genes with monoallelic expression, the abnormal gene dosage induced by the aneuploid chromosome is maybe due to the number of cells expressing the gene. This difference in the fraction of expressing cells could contribute to the development and the variability of phenotypes in aneuploidies. This study provides a new fundamental understanding of the allele specific expression in T21 and other aneuploidies.

**Disclosure of interest**

None declared.

## O7 Role of microRNA in LCL to IPSC reprogramming

### S. Kumar^1^, J. Curran^2^, J. Blangero^2^

#### ^1^South Texas Diabetes and Obesity Institute, School of Medicine, University of Texas Rio-Grande Valley, Edinburg, TX, USA; ^2^South Texas Diabetes and Obesity Institute, School of Medicine, University of Texas Rio-Grande Valley, Brownsville, TX, USA

##### **Correspondence:** S. Kumar – South Texas Diabetes and Obesity Institute, School of Medicine, University of Texas Rio-Grande Valley, Edinburg, TX, USA

**Objectives**

A large number of EBV immortalized lymphoblastoid cell lines (LCLs) have been generated and maintained in genetic/epidemiological studies as a perpetual source of DNA and as a surrogate *in-vitro* cell model. Recent successes in reprograming LCLs into induced pluripotent stem cells (iPSCs) have paved the way to generate more relevant *in-vitro* disease models using this existing bio-resource. However the effects of EBV encoded oncoproteins on cellular transcription and function make LCLs a unique biomaterial to reprogramme. Accumulating evidence now provides support that miRNAs play a critical role in transcription factor-induced reprogramming of iPSCs.

**Methods**

To investigate the role of miRNAs in regulating gene expression and cellular functions during LCL to iPSC reprogramming, we performed a parallel genome-wide miRNA and mRNA expression analysis in six LCLs and their reprogrammed iPSCs.

**Results**

A total of 77 miRNAs and 5,228 mRNAs were significantly (*FC-abs ≥ 2.0* and *FDR ≤ 0.05)* differentially expressed (DE) during LCL to iPSC reprogramming out of which 29 miRNAs and 2,317 mRNAs were significantly down-regulated and 48 miRNAs and 2,911mRNAs were significantly up-regulated. The down-regulated miRNAs were highly enriched for LCL specific miRNAs (*miR-155, let-7a-i, miR-21, miR-142, miR103, miR-320, miR-146a-b*) and the up-regulated miRNAs were highly enriched for iPSC specific miRNAs (*miR-302a, miR-302c, miR-371a, miR-302b, miR-302d, miR-372, miR-373miR-92a-1, miR-92a-2, miR-92b, miR-17, miR-20a, miR-18a*). Further we performed target prediction analysis for all the significantly DE miRNAs using the miRNA target prediction data bases. The 3,456 genes were predicted to be the targets of the 29 miRNAs that were significantly down-regulated during LCL to iPSC reprogramming. Out of these 3,456 predicted target genes 1,023 were significantly DE during LCL to iPSC reprogramming. For the 48 miRNAs that were significantly up-regulated during LCL to iPSC reprogramming 5,063 target genes were predicted out of which 1,462 were significantly DE during LCL to iPSC reprogramming. The significantly DE genes that were also the predicted targets of the significantly down or up regulated miRNAs were further analyzed for functional annotations and pathway analysis using Ingenuity Pathway Analysis Platform.

**Conclusion**

In summary, our analysis identifies DE miRNAs and their DE target genes and a global role of miRNAs in broad resetting of cellular transcriptome and function during LCL to iPSC reprogramming.

**Disclosure of interest**

None declared.

## O8 Multiple enhancer variants disrupt gene regulatory network in Hirschsprung disease

### S. Chatterjee^1^, A. Kapoor^1^, J. Akiyama^2^, D. Auer^1^, C. Berrios^1^, L. Pennacchio^2^, A. Chakravarti^1^

#### ^1^Institute of Genetic Medicine, Johns Hopkins University, Baltimore, MD, USA; ^2^Genomics Division, Lawrence Berkeley National Laboratory, Berkeley, CA, USA

##### **Correspondence:** S. Chatterjee – Institute of Genetic Medicine, Johns Hopkins University, Baltimore, MD, USA

**Objectives**

Common sequence variation in cis-regulatory elements (CREs) are the suspected etiological causes of complex disorders. We examined all common (>10% minor allele frequency) non-coding variants within a ~153kb locus surrounding the gene for receptor tyrosine kinase *RET*, which is most commonly mutated in Hirschsprung disease (HSCR or congenital aganglionosis), a form of functional intestinal obstruction in neonates (1 in 5,000 live births). We hoped to find all causal non-coding polymorphisms disrupting enhancer function leading to the disease.

**Methods**

We used human and mouse fetal gut at relevant developmental time points for transcriptional profiling, ChIP assays, transgenic enhancer assays and siRNA mediate knockdowns of relevant transcription factors.

**Results**

We demonstrate that: (i) the three polymorphisms residing in 3 distinct enhancers that increase risk of the disease by 4-, 2- and 1.7-fold. Haplotypes for these three independent variants display wide variation in risk. (ii) the three CREs are *Ret* enhancers with distinct temporal activities during mouse gut development; (iii) the CREs are bound by the transcription factors Rarb, Gata2/3 and Sox10, respectively, each developmentally expressed concordant with its cognate enhancer activity; (iv) variants in these CREs lead to their loss of activity and reduce *Ret* expression; (v) Ret is a positive feedback regulator of *Sox10* and *Gata2/3* transcription; and, (vi) additional feedback interactions affect its ligand *Gdnf*, co-receptor *Gfra1* and signal terminator *Cbl*.

**Conclusion**

These results explain how individually common, small effect non-coding polymorphisms can lead to large genetic effects in HSCR, since transcription attenuation of Ret from enhancer mutations are amplified through its auto-regulation. These results implicate RET as a key rate limiting step in early enteric nervous system (ENS) development and explains why >95% of HSCR cases have at least one *RET* loss-of-function allele. More generally, the phenotypic impact of a complex disorder can only be understood by assessing gene effects in the context of their gene regulatory networks.

**Disclosure of interest**

None declared.

## O9 Metabolomic profiling for the diagnosis of neurometabolic disorders

### T. R. Donti^1^, G. Cappuccio^2^, M. Miller^1^, P. Atwal^1^, A. Kennedy^3^, A. Cardon^4^, C. Bacino^1^, L. Emrick^4^, J. Hertecant^5^, F. Baumer^6^, B. Porter^6^, M. Bainbridge^1^, P. Bonnen^1^, B. Graham^1^, R. Sutton^1^, Q. Sun^1^, S. Elsea^1^

#### ^1^Molecular and Human Genetics, Baylor College of Medicine, Houston, TX, USA; ^2^Department of Translational Medical Sciences, Federico II University, Naples, Italy; ^3^Metabolon Inc, Durham, NC, USA; ^4^Section of Pediatric Neurology and Neuroscience, Baylor College of Medicine, Houston, TX, USA; ^5^Tawam Hospital, Abu Dhabi, United Arab Emirates; ^6^Stanford Medical School, Stanford, CA, USA

##### **Correspondence:** T. R. Donti – Molecular and Human Genetics, Baylor College of Medicine, Houston, TX, USA

**Objectives**

In individuals presenting with undifferentiated phenotypes such as developmental delay, hypotonia, and seizures, the list of differential diagnoses is often very long and includes metabolic/neurometabolic, genomic, and other Mendelian disorders. Here we want to demonstrate the utility of untargeted metabolomic profiling to screen for a wide range of neurometabolic disorders.

**Methods**

Untargeted small molecule metabolomic profiling was performed as described previously [1] on plasma samples from 12 patients suspected to have a neurometabolic disorder with a presentation of seizures, developmental delay and hypotonia.

**Results**

We identified 5 different neurometabolic disorders in these 12 patients. We observed elevations of 3-methoxytyrosine and decreased levels of dopamine and vanillylmandelate in AADC deficiency, elevations of 2-pyrrolidinone in ABAT deficiency, elevations of succinyladenosine in ADSL deficiency, increased citrate in citrate transporter deficiency, and elevations of imidazole propionic acid, cis and trans-urocanate in urocanase deficiency. The perturbations in the metabolomic profiles of plasma from these patients are unique, specific and not previously seen in over 300 other samples analyzed as normal controls or for other indications.

**Conclusion**

The standard diagnostic test for AADC, ABAT, and ADSL deficiency is CSF neurotransmitter analysis, while testing for urocanase deficiency requires an enzyme activity assay from a liver biopsy. These cases demonstrate the ability of untargeted metabolomic profiling for the functional confirmation of pathogenicity of VUS found via WES; moreover, disorders for which there is no biochemical testing or where testing is only available on CSF are able to be diagnosed in a plasma sample. This also demonstrates the utility of metabolomic profiling alone to screen for a wide range of neurometabolic disorders.

**References**

1. Miller M, Kennedy A, Eckhart A, Burrage L, Wulff J, Miller LD, Milburn M, Ryals J, Beaudet A, Sun Q*et al*: Untargeted metabolomic analysis for the clinical screening of inborn errors of metabolism. *J Inherit Metab Dis* 2015:1–11.

**Disclosure of interest**

None declared.

## O10 A novel causal methylation network approach to Alzheimer’s disease

### Z. Hu^1^, P. Wang^2^, Y. Zhu^3^, J. Zhao^3^, M. Xiong^2^ and David A Bennett^4^

#### ^1^School of Public Health, Houston Health Science Center, Houston, TX, USA; ^2^University of Texas, Houston Health Science Center, Houston, TX, USA; ^3^Tulane University, New Orleans, LO, USA; ^4^ Rush Alzheimer’s Disease Center, Rush University, Chicago, IL, USA

##### **Correspondence:** Z. Hu – School of Public Health, Houston Health Science Center, Houston, TX, USA

**Objectives**

Alzheimer’s disease (AD) is the most common progressive neurodegenerative disease and represents a major cause of disability for elderly patients. DNA methylation–are increasingly seen as playing an important role in AD development. However its causal mechanisms remain unclear. Recent studies indicate that AD develops essentially as a result of dysfunction of molecular networks. Our purpose is develop large-scale causal methylation networks to uncover the mechanism of AD development.

**Methods**

We propose to use causal graphs as a major concept and a general framework for causal methylation network analysis and develop “score and search”-based methods for exact learning causal graphs of methylation networks to find the best-scoring structures for a given methylation dataset. Specifically, we develop novel functional structural equations for modeling methylation networks and use integer programming to search the network with optimal score.

**Results**

The proposed methods were applied to AD data with 460045 CpG sites from 748 samples. At the first stage, the methylation data of 168 gene from the pathway ‘Alzheimer’s disease were used to create a causal network describing the connection among the methylation sites between these genes. According to the current result, 148 gene was matched and tested in the model. We identified a largest connected causal methylation network with 47 nodes and 96 edges. Most genes were confirmed to play an important role in the AD development from the literature.

**Conclusion**

The proposed methods provide a highly flexible general framework for causal methylation network analysis and provide more rich information than co-methylation network. The exact learning algorithms will guarantee to find optimal solutions and hence provide accurate estimations of causal graphs of methylation networks. The causal methylation networks are able to uncover the mechanism of AD development.

**Disclosure of interest**

None declared.

## O11 A microRNA signature identifies subtypes of triple-negative breast cancer and reveals MIR-342-3P as regulator of a lactate metabolic pathway

### A. Hidalgo-Miranda^1^, S. Romero-Cordoba^1^, S. Rodriguez-Cuevas^2^, R. Rebollar-Vega^1^, E. Tagliabue^3^, M. Iorio^3^, E. D’Ippolito^3^, S. Baroni^3^

#### ^1^Cancer Genomics Laboratory, National Institute Of Genomic Medicine (INMEGEN), Mexico City, Mexico; ^2^FUCAM , Mexico City, Mexico; ^3^National Tumor Institute, Milan, Italy

##### **Correspondence:** A. Hidalgo-Miranda – Cancer Genomics Laboratory, National Institute Of Genomic Medicine (INMEGEN), Mexico City, Mexico

**Objectives**

Triple negative breast cancer (TNBC) represents a challenging tumor type due to their poor prognosis and limited treatment options. It is well recognize that clinical and molecular heterogeneity of TNBC is driven in part by post-transcriptional regulators such as miRNAs. To stratify TNBCs, we profiled 1050 miRNAs in 132 adjuvant TNBC tumors and 40 tumors from other immunophenotypes using an Affymetrix microarray platform.

**Methods**

A NMF clustering analysis allowed us to identify 4 TNBC subtypes featuring unique miRNA expression patterns, disease free and overall survival rates and particular gene ontology enrichments. Our agglomerative approach was cross-validated by using two other clustering algorithms. 3 cell line models were classified according to our miRNA signature, recapitulating two different miRNA subgroups. The TNBC tumors were compared against other phenotypes identifying differentially expressed miRNAs to define interesting miRNAs for further functional analysis.

**Results**

We found low expression levels of miR-342-3p in TNBC tumors compared with other breast cancer phenotypes, and this down-regulation characterizes one of our miRNA subgroups with high risk to relapse. To characterize its functional role, miR-342-3p was transiently transfected in the cell line MDA-MB-468, showing a decrease in cell proliferation, viability and migration rates. A gene expression profile revealed 140 altered mRNAs, from which 35 are potential direct targets of miR-342-3p defined by an in-silico analysis. The monocarboxylate transporter 1(MCT1), was confirmed as one target of miR-342-3p by a luciferase assay and western blot analysis. MCT1 repression by the miRNA promotes lactate efflux changes in the tumor cells, reflected in the accumulation of exogenous lactate and the increase in levels of extracellular endogenous lactate together with a decrease level of intra and inter cellular glucose concentration.

**Conclusion**

These data suggest a metabolic change that favors a more glycolytic environment, which lead to a glucose deprivation context that may contribute to the reduction in proliferation, viability and migration capabilities already described.

**Disclosure of interest**

None declared.

## O12 Transcriptome analysis identifies genes, enhancer RNAs and repetitive elements that are recurrently deregulated across multiple cancer types

### B. Kaczkowski^1^, Y. Tanaka^2^, H. Kawaji^2^, A. Sandelin^3^, R. Andersson^3^, M. Itoh^1^, T. Lassmann^4^, the FANTOM5 consortium^1^, Y. Hayashizaki^5^, P. Carninci^1^, A. R. R. Forrest^6^

#### ^1^Division of Genomic Technologies, RIKEN Center for Life Science Technologies, Yokohama, Japan; ^2^Preventive Medicine and Applied Genomics unit, RIKEN Advanced Center for Computing and Communication, Yokohama, Japan; ^3^Department of Biology, University of Copenhagen, Copenhagen, Denmark; ^4^Telethon Kids Institute, the University of Western Australia, Perth, Australia; ^5^RIKEN Preventive Medicine & Diagnosis Innovation Program, Wako, Japan; ^6^Harry Perkins Institute of Medical Research, the University of Western Australia, Nedlands, Australia

##### **Correspondence:** B. Kaczkowski – Division of Genomic Technologies, RIKEN Center for Life Science Technologies, Yokohama, Japan

**Objectives**

We aim to find genes that are frequently deregulated in cancer and thus can be useful as diagnostic markers for early detection, and potentially as therapeutic targets. We focus on biomarkers with pan-cancer potential that can be applicable to multiple cancer types.

**Methods**

We used the Cap Analysis of Gene Expression (CAGE) profiles of 225 cancer cell lines and 339 normal primary cells from FANTOM5 project. CAGE is a 5′ sequence tag technology that enables promoter-level expression analysis and can be used to estimate the activity of enhancers from bidirectional transcription of enhancer RNAs. As a complementary data set, we used RNA-seq data from 14 tumor types profiled by The Cancer Genome Atlas (TCGA). In both data sets (FANTOM5 and TCGA), we performed cancer vs. normal differential expression analysis in all cancer types.

**Results**

We identified a set of pan-cancer markers (of both coding and non-coding transcripts) that are recurrently perturbed in both the cancer cell lines (FANTOM5) and clinical tumors (TCGA). The FANTOM5 CAGE data provided novel insights into cancer transcriptome. We used the genomic location of the CAGE TSSs to show that promoters that overlap repetitive elements (especially SINE/Alu and LTR/ERV1 elements) are often upregulated in cancer. Specifically, a little known repeat family, REP522 (~1.8Kb in size, largely palindromic, unclassified interspersed repeat), was strongly enriched for the most cancer-activated promoters. Here we present previously un-published, follow-up results that detail the REP522 activation in cancer. Finally, we present 90 enhancers that are activated in cancer cell lines. With ENCODE ChIA-PET data, we linked 16 of those enhancers to promoters of known cancer genes.

**Conclusion**

Our transcriptome analysis identified candidate biomarkers with pan-cancer potential and provided new insights into enhancers and repetitive elements that are recurrently activated in cancer.

**References**

1. Kaczkowski B, et al. Transcriptome analysis of recurrently deregulated genes across multiple cancers identifies new pan-cancer biomarkers. *Cancer Research*. 2015; 76(2): 216–226.

**Disclosure of interest**

None declared.

## O13 Elevated mutation and widespread loss of constraint at regulatory and architectural binding sites across 11 tumour types

### C. A. Semple

#### MRC Human Genetics Unit, MRC Institute of Genetics and Molecular Medicine, Edinburgh, UK

**Objectives**

Disruption of gene regulation is thought to play major roles in carcinogenesis and tumour progression. Here, we characterize the mutational profiles of diverse transcription factor binding sites (TFBSs) across 1,574 completely sequenced cancer genomes encompassing 11 tumour types. We assess the relative rates and impact of mutation at the binding sites of 87 different transcription factors (TFs) by comparing the abundance and patterns of single base substitutions within putatively functional binding sites to matched control sites.

**Methods**

To detect putatively regulatory binding sites in the genome, we used a combination of computational prediction and experimental data. Position weight matrices for 118 transcription factor binding motifs were used to find TFBS motif matches in the genome. We intersected these motif matches with experimentally defined open chromatin regions to define putatively functional TFBSs. Motif matches not occurring within open chromatin were used as control, putatively non-functional sites. Comparisons between these functional and control sites underlie our methods, and we develop novel metrics to assess the relative rates and functional impact of cancer mutations at putatively funcitonal regulatory sites.

**Results**

We observe a strong and significant excess of mutations at functional binding sites across TFs, and show that the substitutions that accumulate in cancers are often more disruptive than those that are tolerated as germline variants. Putatively functional CTCF binding sites suffer an exceptionally high mutational load in cancer relative to control sites, and those involved in the architecture of higher order chromatin structures are the most highly mutated. The mutational load at CTCF-binding sites appears to be dominantly determined by replication timing and the mutational signature of the tumor sample in question, suggesting that selectively neutral processes underlie the unusual mutation patterns seen at CTCF sites across tumor types.

**Conclusion**

We show that mutations at active TFBSs are common in tumours, they appear to accumulate largely unchecked by selective processes and are independent of mutations in coding sequences, exhibiting distinct rates among tumor types. Our study thus underlines the functional importance and fragility of the regulatory genome in cancer.

**Disclosure of interest**

None declared.

## O14 Exome sequencing provides evidence of pathogenicity for genes implicated in colorectal cancer

### E. A. Rosenthal^1^, B. Shirts^1^, L. Amendola^1^, C. Gallego^2^, M. Horike-Pyne^1^, A. Burt^1^, P. Robertson^1^, P. Beyers^1^, C. Nefcy^1^, D. Veenstra^1^, F. Hisama^1^, R. Bennett^1^, M. Dorschner^1^, D. Nickerson^1^, J. Smith^1^, K. Patterson^1^, D. Crosslin^1^, R. Nassir^3^, N. Zubair^4^, T. Harrison^4^, U. Peters^1,4^, G. Jarvik^1^, NHLBI GO Exome Sequencing Project

#### ^1^Univ of Washington, Seattle, WA, USA; ^2^Univ of Michigan, Ann Arbor, MI, USA; ^3^Univ California, Davis, CA, USA; ^4^Fred Hutch, Seattle, WA, USA

##### **Correspondence:** E. A. Rosenthal – Univ of Washington, Seattle, WA, USA

**Objectives**

In the U.S., the lifetime risk of developing colorectal cancer (CRC) is 4.5%. ~5% of cases carry an identified pathogenic variant in known causal genes. In another 20%, CRC appears inherited but no known pathogenic variant has been detected. This is partly due to lack of evidence to classify variants as pathogenic when they occur in genes that are implicated (GWAS, linkage, or biological pathway information), but not proven, to be associated with CRC.

**Methods**

To find evidence of association for 1128 suspected CRC associated genes, we compared the number of rare (MAF < 0.005), potentially disruptive variants (PDV) (stop gain (SG), splice acceptor/donor change (SA, SD), and frameshift (FS)) found in 169 CRC cases and 3524 controls. Cases included individuals from the Clinical Sequencing Exploratory Research NEXT Medicine study (CSER, N=78), Women’s Health Initiative (WHI, N=76), and Northwest Institute of Genetic Medicine Family Polyposis Study (NWIGM, N=15). Controls were selected randomly with respect to CRC from the Exome Sequencing Project (ESP), and were not known to have any Lynch associated cancers or to carry a known pathogenic CRC variant.

**Results**

We found a significant association between case status and rare PDV carrier status: 25% of CRC cases carried a rare PDV compared to 4% of controls (p<2e-16, OR 8.1). 96 genes had 174 rare PDVs: 92 SGs, 24 SDs, 15 SAs and 43 FSs. Rare PDVs occurred in cases only for 35 genes, in controls only for 55 genes, and in both for 6 genes. Among cases, there were 11 SGs, 2 SDs, 6 SAs and 26 FSs. Among controls, there were 81 SGs, 22 SDs, 9 SAs and 17 FSs. Case specific genes had ≤2 rare PDVs: those with 2 were *MKL2* and *PMS1*. Interestingly, *ATM*, *ATR* and *BRCA2* contained PDVs in both cases and controls.

**Conclusion**

We show the power of aggregate information to find support for disease association in a subset of CRC implicated genes. Additionally, we provide evidence that *ATM*, *ATR* and *BRCA2* may be associated with CRC in addition to the known association with breast cancer.

**Disclosure of interest**

None declared.

## O15 The tandem duplicator phenotype as a distinct genomic configuration in cancer

### F. Menghi^1^, K. Inaki^1^, X. Woo^1^, P. Kumar^1^, K. Grzeda^1^, A. Malhotra^1^, H. Kim^1^, D. Ucar^1^, P. Shreckengast^1^, K. Karuturi^1^, J. Keck^2^, J. Chuang^1^, E. T. Liu^1^

#### ^1^The Jackson Laboratory, Farmington, CT, USA; ^2^The Jackson Laboratory, Sacramento, CA, USA

##### **Correspondence:** F. Menghi – The Jackson Laboratory, Farmington, CT, USA

**Objectives**

Next generation sequencing studies have revealed genome-wide structural variation patterns in cancer, such as chromothripsis and chromoplexy that do not engage a single discernable driver mutation, and that currently have no clinical relevance. We aimed at a detailed molecular characterization of one of these genomic configurations, the tandem duplicator phenotype (TDP).

**Methods**

We combined whole genome sequencing (WGS) data from 277 human genomes representing 11 cancer types and devised a robust genomic metric able to identify cancers with a chromotype called tandem duplicator phenotype (TDP) characterized by frequent and distributed tandem duplications (TDs).

**Results**

Enriched only in triple negative breast, ovarian, endometrial, and liver cancers, TDP tumors conjointly exhibit *TP53*-mutations, low expression of *BRCA1*, and increased expression of DNA replication genes pointing at re-replication in a defective checkpoint environment as a plausible causal mechanism. The resultant TDs in TDP augment global oncogene expression and disrupt tumor suppressor genes. Importantly the TDP strongly correlates with cisplatin sensitivity in both triple negative breast cancer cell lines and primary patient-derived xenografts.

**Conclusion**

We conclude that the TDP is a common cancer chromotype that coordinately alters oncogene/tumor suppressor expression with potential as a marker for chemotherapeutic response.

**Disclosure of interest**

None declared.

## O16 Modeling genetic interactions associated with molecular subtypes of breast cancer

### B. Ji, A. Tyler, G. Ananda, G. Carter

#### The Jackson Laboratory, Bar Harbor, ME, USA

##### **Correspondence:** G. Carter – The Jackson Laboratory, Bar Harbor, ME, USA

**Objectives**

The characterization of mRNA-expression subtypes in breast cancer facilitates genomic and genetic studies to identify biological processes that drive distinct molecular subtypes and elucidates the potential feasibility of subtype-specific drug targets. However, such therapies tend to have limited efficacy, often due to unpredicted compensation in the network of mutations. Polygenic models that account for multiple somatic mutations and their interactions can potentially improve target selection and provide a more detailed view of tumor genetic architecture.

**Methods**

We addressed this problem with a multi-trait genetic interaction analysis of copy-number variation and gene expression data from breast cancer samples in The Cancer Genome Atlas. Modules of co-expressed genes were derived and assessed for biological function and genetic association with mutations in oncogenes and tumor suppressors. Summary module phenotypes with pleiotropic associated loci were simultaneously analyzed to infer direct genetic effects as well as effects mediated by genetic interactions for each module.

**Results**

We observed widespread evidence of genetic redundancy, in which two mutations combine to yield a less than additive effect that is similar to either mutation in isolation. In addition, we also identified interacting mutations that combinatorially associate with distinct modules and subtypes in a non-additive manner. These somatic mutant combinations were often predictive of molecular subtypes when single mutations were not.

**Conclusion**

Accounting for interactions among somatic mutations in tumor samples reveals high genetic redundancy and complex regulatory hypotheses for breast cancer subtypes. Our work demonstrates how integrative genetic and genomic analysis can be used to generate more precise hypotheses for tumor genetics, which may be used to prioritize therapeutic targets for robust tumor suppression.

**Disclosure of interest**

None declared.

## O17 Recurrent somatic mutation in the MYC associated factor X in brain tumors

### H. Nikbakht^1^, M. Montagne^2^, M. Zeinieh^1^, A. Harutyunyan^1^, M. Mcconechy^1^, N. Jabado^3^, P. Lavigne^2^, J. Majewski^1^

#### ^1^Human Genetics, McGill University, Montreal, Quebec, Canada; ^2^Biochemistry, Sherbrooke University, Sherbrooke, Quebec, Canada; ^3^Pediatrics, McGill University, Montreal, Quebec, Canada

##### **Correspondence:** H. Nikbakht – Human Genetics, McGill University, Montreal, Quebec, Canada

**Objectives**

Activation of MYC pathway has been shown in diverse cancers. MYC Associated Factor X (MAX) plays a key role in the MYC-MAX-MAD gene regulatory network; however, its direct involvement in cancer has not yet been reported. Here we report discovery of a novel recurrent somatic mutation in MAX in brain tumors and study its effects on the development and progression of tumors.

**Methods**

We found a mutation on Arg 51 residue to Glu in MAX gene in a patient with bilateral thalamic pediatric astrocytoma in which the primary tumors (left and right thalamus) had nearly identical mutation profiles except for the presence of the MAX R51Q only in one.

We used this unique opportunity to study the effects of this mutation on the progression and development of brain tumors.

We performed differential expression on these samples to find the pathways affected by this mutation. Using ChipSeq we studied changes in the chromatin conformation in genes regulated by this network. We used CD experiments, to study how this mutation affects the affinity between Max and other proteins in this family and with DNA.

**Results**

We screened our dataset and found 7 cases in 180 HGAs exome sequenced by our group (3.8%) with this mutation. We also identified 14 cases in published datasets.

We found that this mutation always appears later in tumor development in subclonal fashion and is accompanied by at least one driver such as H3 K27M.

Our differential expression and ChipSeq experiments revealed lack of a global effect of this mutation but specific effects on groups of genes involved in some pathways such as apoptosis.

We also demonstrate that this mutation has no effect on the binding efficacy between proteins in its regulatory network, but a Max R51Q homodimer binds less efficiently to nonspecific DNA than its wild type. It however, only affects the binding between Myc/Max heterodimer to DNA in E-boxes.

**Conclusion**

We identify MAX as a new cancer gene, particularly relevant to brain cancer.

Our results show the possible effects of MAX mutation in promoting tumor progression and development. It also suggests the effect of this mutation on the spread of the tumor.

These findings shed new light on the mechanisms underlying cancer progression and the involvement of MYC signalling in development of brain tumors which, in turn, can point us towards new targets for therapeutic approaches.

**Disclosure of interest**

None declared.

## O18 Predictive biomarkers to metastatic pancreatic cancer treatment

### J. B. Goldstein^1^, M. Overman^2^, G. Varadhachary^2^, R. Shroff^2^, R. Wolff^2^, M. Javle^2^, A. Futreal^1^, D. Fogelman^2^

#### ^1^Genomic Medicine, MD Anderson Cancer Center, Houston, TX, USA; ^2^Gastrointestinal Medical Oncology, MD Anderson Cancer Center, Houston, TX, USA

##### **Correspondence:** J. B. Goldstein – Genomic Medicine, MD Anderson Cancer Center, Houston, TX, USA

**Objectives**

In 2015, we demonstrated that a strong family history of BRCA related tumors portends a better prognosis in metastatic pancreatic cancer patients. We now investigate if this holds true for more recent patients treated with standard-of-care FOLFIRINOX (FNX) or Gemcitabine/nab-paclitaxel (GA). We hypothesize that targeted sequencing of these tumors for DNA repair pathway aberrations will better predict outcomes than the surrogate marker of family history, which may be subject to patient bias.

**Methods**

We identified patients with de novo stage 4 pancreatic cancer initially treated at MD Anderson Cancer Center with first-line FNX or GA. We excluded patients with prior surgical resection (bypass was allowed) or radiation as initial therapy, and patients with unknown family history. Survival analysis was performed using the Kaplan-Meier method.

**Results**

We identified 153 patients initially treated with FNX and 80 patients treated with GA. Median age of the entire cohort was 62 years (36–84), 58% were male. Median OS was 286 and 295 days, respectively. Approximately 5% of patients had a family history of 3 or more BRCA tumors (breast, ovarian, prostate, pancreas). Median OS for these patients was 469 days, as compared to 285, 268, and 296 days for patients with 0, 1, and 2 affected family members. Median survival in patients with 3+ family members affected was 463 days and 511 days for patients on FNX and GA respectively (95% CI 240-636d, 0–1092 d). For patients with 0–2 such family members, median OS was 283 and 268 days, respectively. As expected, ECOG 0–1 and the absence of liver metastases were associated with longer survival. We identified 126 of 153 FNX patients and 51 of 80 GA patients with pathology specimens available for targeted sequencing.

**Conclusion**

As in our earlier report, we see a trend towards increased survival in patients with 3 or more family members with BRCA related tumors. However, the small number of these patients precludes a definitive assessment. We believe that targeted sequencing of DNA repair pathway and associated genes will better elucidate the mechanism of survival benefit over biased family history.

**References**

1. Fogelman D, Sugar EA, Oliver G, et al. Family history as a marker of platinum sensitivity in pancreatic adenocarcinoma. Cancer chemotherapy and pharmacology. 2015. 76(3): 489–498.

**Disclosure of interest**

None declared.

**Fig. 1 (abstract O18).**
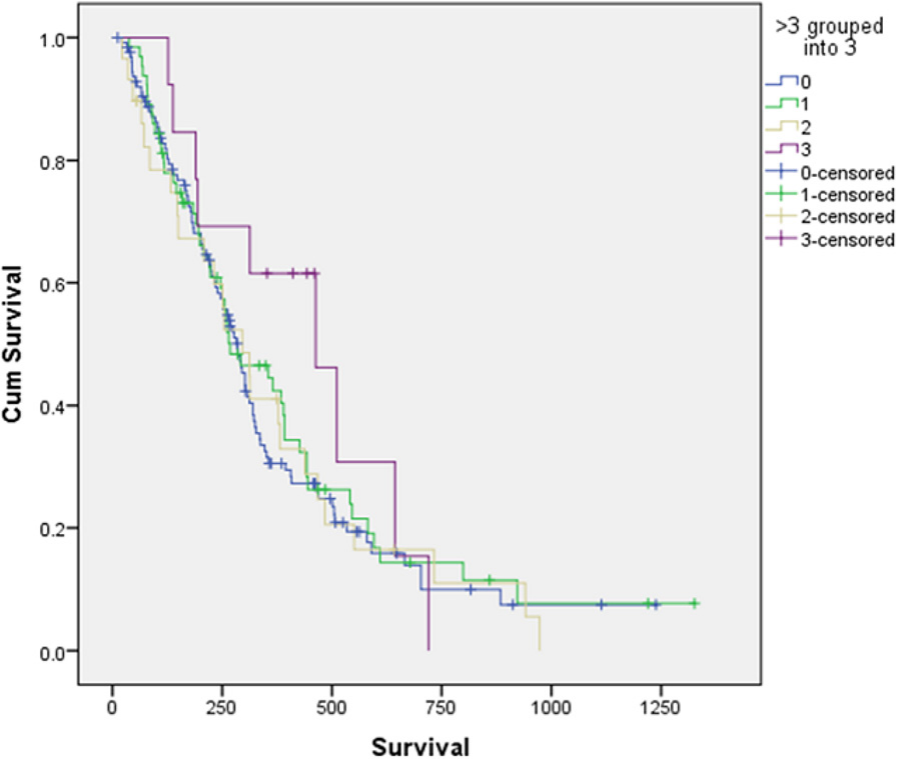
Overall survival based on number of family members with BRCA associated

## O19 DDIT4 gene expression as a prognostic marker in several malignant tumors

### L. Bravo^1^, W. Fajardo^1^, H. Gomez^2^, C. Castaneda^2^, C. Rolfo^3^, J. A. Pinto^2^

#### ^1^Escuela de Medicina Humana, Universidad Privada San Juan Bautista, Lima, Peru; ^2^Unidad de Investigación Básica y Traslacional, Oncosalud-AUNA, Lima, Peru; ^3^Oncology Department, University Hospital Antwerp, Antwerp, Belgium

##### **Correspondence:** J. A. Pinto – Unidad de Investigación Básica y Traslacional, Oncosalud-AUNA, Lima, Peru

**Objectives**

The DDIT4 gene (DNA-damage-inducible transcript 4) encodes a protein related to adverse environmental conditions, whose action is the inhibition of mTOR. In a recent work we found DDIT4 levels was associated with the outcome in triple negative breast cancer (J Clin Oncol 33, 2015 (suppl; abstr 1097)). There are not previous reports relating DDIT4 with prognosis of cancer patients. Our aim in this study was to explore the influence of this gene in several types of malignant tumors.

**Methods**

We evaluated the influence of DDIT4 expression in the outcome (either, disease-free survival or progression-free survival or overall survival). Univariate Cox regression analysis of DDIT4 in the online platforms KM Plotter (http://kmplot.com/) and SurvExpress (http://bioinformatica.mty.itesm.mx/) was done. Datasets were split based in the median of expression. Cancer types evaluated include: Acute Myeloid Leukemia, Brain Cancer, Breast Cancer, Bone Cancer, Cervical Cancer, Head and Neck Cancer, Hematological Cancer, Liver Cancer, Lung Cancer, Pancreatic Cancer, Ovarian Cancer and Prostate Cancer.

**Results**

A high level of DDIT4 was significantly associated with a worse outcome in breast cancer (n=4142), P=3x10^−14^ (HR=1.64, CI95%: 1.44-1.87); acute myeloid leukemia (n=168), P=3.47x10^−5^ (HR=2.31, CI95%:1.55-3.43), glioblastoma multiforme (n=538), P=0.005809 (HR=1.31, CI95%:1.08-1.59); ovarian cancer (n=1648), P=0.0096 (HR=1.2, IC95%:1.04-1.37); head and neck squamous cell carcinoma (n=283), P=0.03347 (HR=1.49, IC95%:1.03-2.15) and lung adenocarcinoma (n=866), P=0.0038 (HR=1.4, CI95%: 1.02-1.91). In contrast, a high level of DDIT4 was associated with a better prognosis in gastric cancer (n=641), P=1.7x10^−6^ (HR=0.62, CI95%:0.5-0.75) and lung squamous cell carcinoma (n=675), P=0.0015 (HR=0.42, IC95%:0.24-0.73). Frequency of structural alteration of DDIT4 found at cbioportal.org, indicate that 0 to 5.1% of primary tumors where DDIT4 was related with the prognostic had mutations, however in breast tumors xenografts, gene amplification occurs in 17.4% elucidating an key role in tumor aggressiveness.

**Conclusion**

DDIT4 is a promising molecular marker for outcome in several types of cancer. In addition elucidation of DDIT4 participation in cancer aggressiveness could lead to improve the therapeutic strategies, mainly those related with mTOR inhibition.

**Disclosure of interest**

None declared.

**Fig. 2 (abstract O19).**
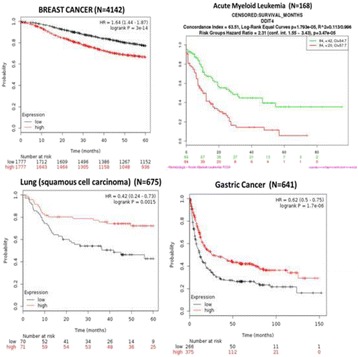
ᅟ

## O20 Spatial organization of the genome and genomic alterations in human cancers

### K. C. Akdemir^1^, L. Chin^2^, A. Futreal^1^, ICGC PCAWG Structural Alterations Group

#### ^1^Genomic Medicine, MD Anderson Cancer Center, Houston, TX, USA; ^2^University of Texas System, Houston, TX, USA

##### **Correspondence:** K. C. Akdemir – Genomic Medicine, MD Anderson Cancer Center, Houston, TX, USA

**Objectives**

The hierarchical folding of genomic DNA within the nucleus is closely related with transcriptional regulation. Recent chromosome conformation studies have suggested that mammalian chromosomes are structured into tissue-invariant topologically associating domains (TADs) where the DNA within a domain is interacting more frequently than with regions in other domains. Genes within the same TADs represent similar gene-expression, histone-modification profiles. Therefore regions separating different TADs (boundaries) have important roles in reinforcing the stability of these domain-wide organizations. TAD boundary disruptions in human limb malformations and cancer lead to dysregulation of certain genes, due to *de novo* promiscuous enhancer exposure to promoters.

Here we sought to identify relationship between genomic architecture and genomic alterations in human cancers.

**Methods**

We utilized approximately 200 thousand somatic genomic alterations (deletions, inversions, duplications) and more than 34 million somatic mutations from 2575 high-coverage whole genome sequencing data across 45 different cancer studies with paired normal samples. We integrated mutations, gene expression and structural alterations with TAD boundaries that we have identified from 5 different human cell lines, representing three different germ layers.

**Results**

Our analysis revealed a strong correlation between the mutational landscape and the TAD organization of the genome. In addition, we found that TAD boundaries inflicted structural alterations that not only affected nearby gene regulation but also the distribution of mutations in human cancers.

**Conclusion**

Structural alterations affecting the spatial organization of the human genome, could lead to dysregulation of genes as well as aberrant mutation distributions in human cancers.

**Disclosure of interest**

None declared.

## O21 Landscape of targeted therapies in solid tumors

### S. Patterson, C. Statz, S. Mockus

#### The Jackson Laboratory for Genomic Medicine, Farmington, CT, USA

##### **Correspondence:** S. Patterson – The Jackson Laboratory for Genomic Medicine, Farmington, CT, USA

**Objectives**

Precision medicine initiatives in oncology focus on specific genetic aberrations as predictive biomarkers for targeted therapies. Next-generation sequencing technologies have driven a projectile shift in patient management through somatic tumor profiling. Due to the rapid pace of this ensuing momentum, it is difficult to grasp such a dynamic landscape. Therefore, an analysis of the targeted therapy landscape was conducted and methods employed are disseminated to foster interoperability among datasets.

**Methods**

A Clinical Knowledgebase (CKB) was created to capture and rapidly retrieve therapeutic information related to patient molecular aberrations. One requirement was the development of a drug class controlled vocabulary to categorize drugs relative to their target specificity, including both pan-level and more gene specific targeted drugs. Additionally, to support capture of combination therapies, one or more single drug entries can be concatenated to a therapy. Drug classes are annotated to molecular variants and therapies are annotated to complex molecular profiles. Using the JAX-CKB, an analysis was conducted on the number and types of targeted therapies for solid tumors associated with 358 genes and in actively recruiting clinical trials.

**Results**

The CKB drug class ontology currently contains 198 terms, consisting of 113 parent and 83 child terms. There are 1006 targeted therapies in CKB and of these, 59 are FDA approved. Pan drug classes include PI3K inhibitors and mTOR inhibitors, which contain 34 and 41 individual drugs within each class, respectively. The drug class VEGFR inhibitors (Pan) has the highest number of hits in actively recruiting clinical trials, and 19 drug classes, including EZH2 inhibitor and p53 activator, are represented in a single solid tumor clinical trial. Furthermore, the most common drug in solid tumor clinical trials is Bevacizumab.

**Conclusion**

Consistency and interoperability of knowledgebases to support clinical next-generation sequencing is pivotal. The JAX-CKB, described here, is built upon controlled vocabularies and ontologies to achieve this mission. Methods on the design and build are shared to foster collaborative processes in this rapidly evolving NGS domain. Furthermore, analysis of content regarding targeted therapies provides an objective view of clinical trial research investigating targeted therapies in solid tumors.

**Disclosure of interest**

None declared.

**Fig. 3 (abstract O21).**
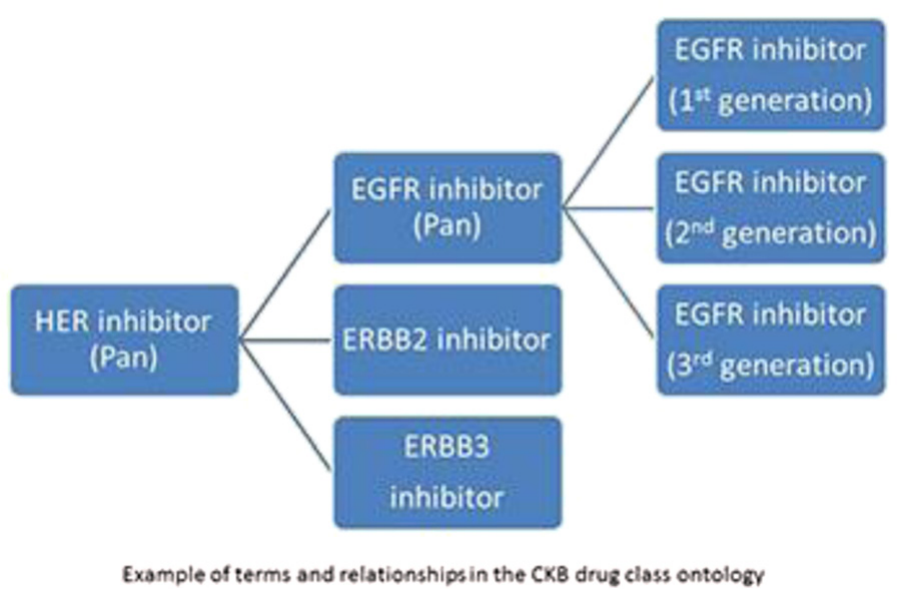
ᅟ

## O22 Genomic analysis reveals novel drivers and progression pathways in skin basal cell carcinoma

### S. N. Nikolaev^1^, X. I. Bonilla^1^, L. Parmentier^2^, B. King^3^, F. Bezrukov^4^, G. Kaya^5^, V. Zoete^6^, V. Seplyarskiy^7^, H. Sharpe^8^, T. McKee^9^, A. Letourneau^10^, P. Ribaux^10^, K. Popadin^10^, N. Basset-Seguin^11^, R. Ben Chaabene^10^, F. Santoni^10^, M. Andrianova^7^, M. Guipponi^12^, M. Garieri^10^, C. Verdan^9^, K. Grosdemange^5^, O. Sumara^13^, M. Eilers^13^, I. Aifantis^3^, O. Michielin^6^, F. de Sauvage^8^, S. Antonarakis^1^

#### ^1^Department of Genetic Medicine and Development, University of Geneva, Geneva, Switzerland; ^2^Department of Dermatology, Hospital of Valais, Sion, Switzerland; ^3^Department of Pathology, NYU School of Medicine, New York, NY, USA; ^4^Department of Physics, University of Connecticut, CT, USA; ^5^Department of Dermatology, University Hospitals of Geneva, Geneva, Switzerland; ^6^Swiss Institute of Bioinformatics, Swiss Institute of Bioinformatics, Lausanne, Switzerland; ^7^Institute of Information Transmission Problems, Russian Academy of Sciences, Moscow, Russian Federation; ^8^Department of Molecular Oncology, Genentech Inc, San Francisco, CA, USA; ^9^Service of Clinical Pathology, University Hospitals of Geneva, Geneva, Switzerland; ^10^Department of Genetic Medicine and Development, University of Geneva Medical School, Geneva, Switzerland, ^11^University of Paris 7, Hospital of Saint-Louis, Paris, France; ^12^Service of Genetic Medicine, University Hospitals of Geneva, Geneva, Switzerland; ^13^Department of Biochemistry and Molecular Biology, University of Würzburg, Würzburg, Germany

##### **Correspondence:** S. N. Nikolaev – Department of Genetic Medicine and Development, University of Geneva, Geneva, Switzerland

**Objectives**

Basal cell carcinoma of the skin (BCC) is the most common malignant neoplasm in humans. BCC is primarily driven by aberrant activation of the Sonic Hedgehog (Hh) pathway. However, its extensive phenotypical variation remains to be explained.

**Methods**

The genetic profiling of 293 BCCs revealed the highest mutation rate observed in cancer (65 Mutations/Mb), with strong prevalence of UV-light signature mutations.

**Results**

85% of BCCs harbored mutations in Hh pathway genes: mutually exclusive *PTCH1* (73%) and *SMO* (20%) (*P=*6.6x10^−8^), *SUFU* (8%), and in *TP53* (61%). 85% of BCCs also harbored additional driver mutations in other genes implicated in BCC tumorigenesis. Recurrent driver mutations were observed in *MYCN* (30%), *PPP6C* (15%), *STK19* (10%),*LATS1* (8%), *ERBB2* (4%), *PIK3CA* (2%), *RAC1* (1%) and *N/K/H-RAS* (2%). Loss of function (LoF) and deleterious missense mutations were observed in *PTPN14* (23%), *RB1* (8%) and *FBXW7* (5%). In line with the mutational profiles detected by DNA sequencing, we observed activation of the Hh pathway as well as upregulation of target genes of the Hippo-YAP pathway and activation of *MYCN* target genes in RNAseq experiments.

**Conclusion**

The functional analysis of the novel tumorigenic driver mutations in *MYCN*, *PTPN14* and *LATS1* demonstrates their relevance in BCC tumorigenesis and provides an expanded molecular understanding of BCC.

**Disclosure of interest**

None declared.

## O23 Identification of differential biomarkers of hepatocellular carcinoma and cholangiocarcinoma via transcriptome microarray meta-analysis

### S. Likhitrattanapisal

#### Department of Biology, Faculty of Science, Mahidol University, Bangkok, Thailand

**Objectives**

Hepatocellular carcinoma (HCC) and cholangiocarcinoma (CCA) are the members of hepato-biliary diseases. As both HCC and CCA arise from similar cell types, they often exert high levels of similarity in terms of phenotypic characteristics, thus leading to difficulties in HCC and CCA differential diagnoses. In this study, a meta-analysis was performed on HCC and CCA transcriptome microarray data for the purpose of investigating differential transcriptome networks and potential biomarkers of CCA and HCC.

**Methods**

Raw data from 9 HCC and CCA gene expression microarray datasets, consisting 1,185 samples in total, were methodologically compiled and analyzed. For determining differentially-expressed genes in the cancers, gene expression were compared between cancer and its respective normal samples (HCC vs Normal Liver and CCA vs Normal Bile Duct) using *t*-test and *k*-fold validation (*P* < 0.05).

**Results**

Comparing to normal samples, 226 differentially-expressed genes were specifically observed in HCC, 249 genes in CCA, and 41 genes in both. Gene Ontology and KEGG pathway enrichment analyses showed different patterns between functional transcriptome networks of HCC and CCA. Cell cycle and glycolysis/gluconeogenesis pathways were specifically affected in HCC whereas complement and coagulation cascades as well as glycine, serine and threonine metabolism were predominantly presented in CCA.

**Conclusion**

Our meta-analysis revealed different dysregulation in transcriptome networks between HCC and CCA. Some genes in these networks were selectively discussed in the context of HCC-CCA transition, unique characteristics of HCC and CCA, and their potentiality as HCC/CCA differential biomarkers.

**References**

1. Franceschini A, Szklarczyk D, Frankild S, Kuhn M, Simonovic M, Roth A, et al. STRING v9.1: protein-protein interaction networks, with increased coverage and integration. Nucleic Acids Res. 2013;41(Database issue):D808–D815.

2. Wang J, Duncan D, Shi Z, Zhang B. WEB-based GEne SeT AnaLysis Toolkit (WebGestalt): update 2013. Nucl Acids Res. 2013;41(W):W77–W83.

3. Xue TC, Zhang BH, Ye SL, Ren ZG. Differentially expressed gene profiles of intrahepatic cholangiocarcinoma, hepatocellular carcinoma, and combined hepatocellular-cholangiocarcinoma by integrated microarray analysis. Tumor Biol. 2015;36(8):5891–5899.

**Disclosure of interest**

None declared.

**Fig. 4 (abstract O23).**
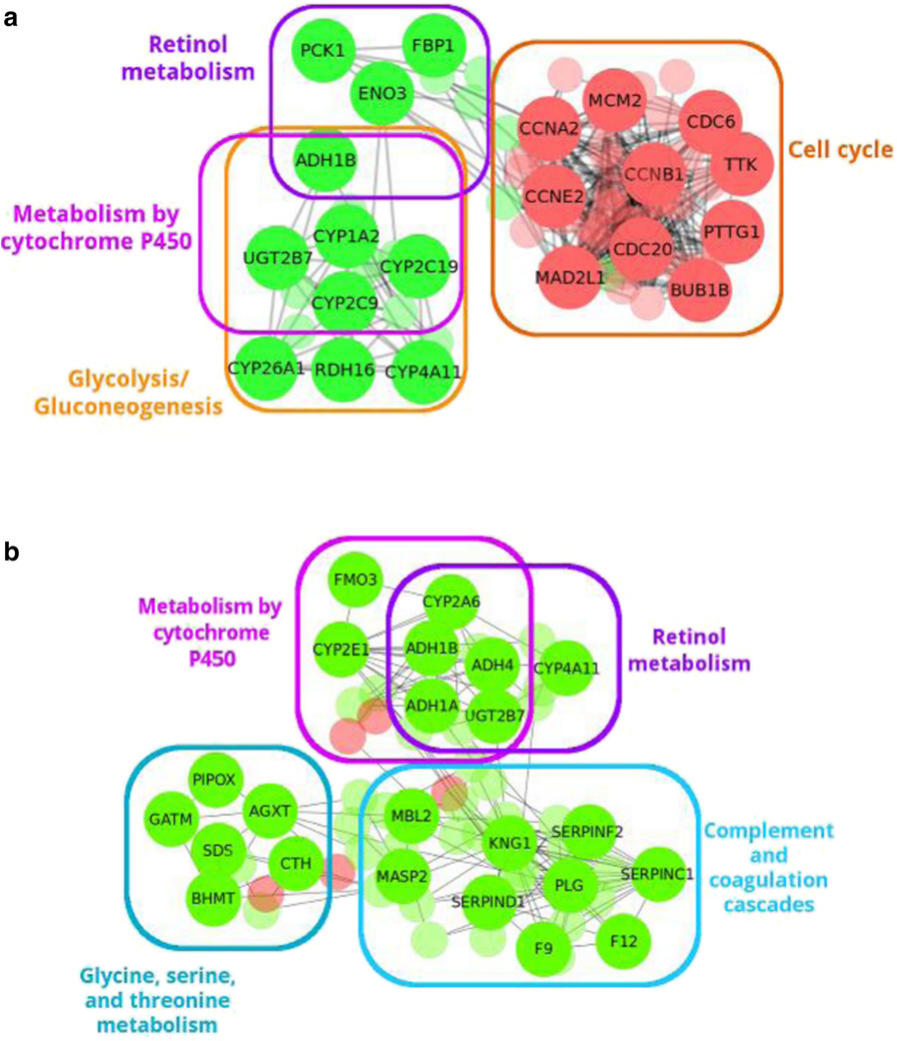
ᅟ

## O24 Clinical validity and actionability of multigene tests for hereditary cancers in a large multi-center study

### S. Lincoln^1^, A. Kurian^2^, A. Desmond^3^, S. Yang^1^, Y. Kobayashi^1^, J. Ford^4^, L. Ellisen^3^

#### ^1^Invitae, San Francisco, CA, USA; ^2^Stanford Medical Center, Palo Alto, CA, USA; ^3^Massachusetts General Hospital, Boston, MA, USA; ^4^Stanford Medical Center, San Francisco, CA, USA

##### **Correspondence:** S. Lincoln – Invitae, San Francisco, CA, USA

**Objectives**

Clinical genetic testing is rapidly evolving with the introduction of next-generation sequencing (NGS) however questions remain about these new tests. First, can NGS methods be deployed that deliver equal or improved performance vs. traditional methods on the full spectrum of (often complex) disease causing variants? Second, do expanded NGS tests provide medical benefits which outweigh the increased uncertainty that naturally follows from testing more genes in more patients? In recently published work [1,2] we tested a large panel of cancer risk genes by NGS in a clinically representative population to evaluate these questions. Here we expand upon that work with additional cases including patients with expanded indications for testing and complex presentations.

**Methods**

We tested a large panel of 25–32 cancer risk genes by NGS in a representative cohort of over 1000 patients meeting current medical guidelines for BRCA1/2 testing. Traditional genetic test results were available for the patients for comparison. Using an interpretation system (Sherloc) based on ACMG 2015 guidelines and employing only publicly available data, variants uncovered by NGS were classified. We established a uniform algorithm based on current practice guidelines to recommend management actions for the non-BRCA1/2 positive individuals, and we evaluated which of these recommendations would represent changes in management above and beyond any recommendations based on personal and family history alone.

**Results**

We find 100% concordance with traditional methods on both sequence and copy-number alterations using a battery of 5 calling algorithms and associated biochemistries., we find 99.8% concordance with BRCA1/2 classifications produced by a different laboratory that uses a large proprietary database. Finally, we find that 52% of genetic test results positive for genes other than BRCA1/2 would warrant consideration of a change in care for mutation-positive patients under current medical practice guidelines.

**Conclusion**

In appropriately referred patients, multi-gene panel testing yields clinically relevant findings with potential management impact for substantially more patients than does BRCA1/2 testing alone.

**References**

1. Lincoln, JMD 2015

2. Desmond, JAMA Oncol 2015

**Disclosure of interest**

S. Lincoln Shareholder of: Invitae, Employee of: Invitae, A. Kurian: None declared., A. Desmond: None declared., S. Yang Shareholder of: Invitae, Employee of: Invitae, Y. Kobayashi Shareholder of: Invitae, Employee of: Invitae, J. Ford Grant/Research Support from: Myriad, Consultant for: Invitae, L. Ellisen Consultant for: GeneDx.

## O25 Correlation with tumor ploidy status is essential for correct determination of genome-wide copy number changes by SNP array

### T. L. Peters^1^, K. R. Alvarez^2^, E. F. Hollingsworth^1^, D. H. Lopez-Terrada^1,2^

#### ^1^Pathology & Immunology, Baylor College of Medicine, Houston, TX, USA; ^2^Pathology, Texas Children’s Hospital, Houston, TX, USA

##### **Correspondence:** T. L. Peters – Pathology & Immunology, Baylor College of Medicine, Houston, TX, USA

**Objectives**

Neuroblastoma, the most common extra-cranial solid tumor in the pediatric population, is a histologically and clinically heterogeneous neoplasm. Tumors characterized only by whole chromosome changes tend to act favorably, whereas tumors with *MYCN* gene amplification and/or segmental chromosomal aberrations have poor outcomes. Given the clinical relevance, our aim was to verify the accuracy of OncoScan FFPE SNP Array (Affymetrix) in assessing genome-wide copy number changes in tumor samples by comparison to other tumor ploidy testing methods.

**Methods**

39 neuroblastic tumors from 38 patients were analyzed using the OncoScan FFPE SNP Array at a pediatric hospital. Tumor slides were macrodissected to increase tumor purity, genomic DNA was isolated and arrays hybridized according to manufacturer’s protocol. Data was analyzed using the OncoScan Console and results viewed using Chromosome Analysis Suite (ChAS) and OncoScan Nexus Express. Copy number results determined by SNP array were compared to ploidy results obtained by karyotype analysis and/or flow cytometry. Additionally, chromosome 1 and 2 aneuploidy data were used as a surrogate ploidy marker in cases where cytogenetics and flow cytometry were unavailable.

**Results**

Data were obtained for 33 of the 39 samples. Review showed that 3 of the 33 samples required recentering of the tumor ploidy baseline. Two samples from 2 lesions in one patient showed widely discordant copy number calls by the software, with one assigned a near tetraploid status and the other a near diploid status. Karyotype analysis confirmed a near tetraploid state. Correlation by karyotype, flow cytometry or chromosome 1 and 2 aneuploidy revealed an additional 4 discordant cases (total of 5/33 cases or 15%).

**Conclusion**

Despite complex algorithms used by the OncoScan software to assign copy number calls, in 15% of the analyzed cases a 2nd method was necessary to correctly assign tumor ploidy baseline. The complex chromosomal copy number changes present in tumors, in addition to tumor impurity, heterogeneity, and poor sample quality, can challenge the software’s ability to correctly assign ploidy state. Our results demonstrate that a second independent method may be necessary in complex tumor cases to correctly assign ploidy that truly reflects tumor biology and that may be necessary for correct patient management.

**Disclosure of interest**

None declared.

## O26 Nanochannel based next-generation mapping for interrogation of clinically relevant structural variation

### A. Hastie, Z. Dzakula, A. W. Pang, E. T. Lam, T. Anantharaman, M. Saghbini, H. Cao, BioNano Genomics

#### BioNano Genomics, Inc., San Diego, CA, USA

##### **Correspondence:** A. Hastie – BioNano Genomics, Inc., San Diego, CA, USA

**Objectives**

Structurally complex loci underlie many diseases. These loci can be very challenging to resolve by currently available methods such as karyotyping, clinical array, PCR-based tests, and NGS. Next-generation mapping by BioNano Genomics Irys® System offers a high-throughput, genome-wide method able to interrogate genome structural differences hundreds of kilobase pairs and span interspersed and even long tandem repeats making it ideally suitable for elucidating the structure and copy number of complex regions of the genome, such as complex pseudogene and paralogous gene families. Clinically relevant regions often contain genes with paralogs and other complex repetitive structures complicating the interpretation of data and diagnosis of disease. We present several examples of genetic loci that can be easily interrogated with genome map data including tandem repeats, paralogous gene families, and loci flanked by segmental duplications.

**Methods**

Some open reading frames or entire genes are randomly amplified with variable copy number such as tRNAs, kringle IV, and *D4Z4*. The LPA gene contains variable copies of a repeat, kringle IV, that results in different lengths of the resultant Lp(a) protein; related to coronary heart disease, cerebrovascular disease, atherosclerosis, thrombosis, and stroke. Tandem repeat is D4Z4, associated with facioscapulohumeral muscular dystrophy (FSHD), with a low copy number (<10 units), occurring in 95% of FSHD cases.

**Results**

We show that the Irys System can accurately measure the copy number of the kringle IV domain and *D4Z4*. A second class of complex structural variation are those that involve genes with paralogs such as amylase and *UGT2B17*, two genes whose copy number have been shown to be involved in human health. We show deletions of *UGT2B17* in a family trio and > 10 different structures at the Amylase region. The third class of genomic variation are those flanked by segmental duplications, especially important because spontaneous rearrangements are common between paralogous segmental duplications causing copy number aberrations and translocation, thus resulting in developmental disorders, such as the 22q11.2 deletion syndrome mediated by segmental duplication rearrangements.

**Conclusion**

We show the assembly of the region, including the normal and pathogenic alleles, using molecules that span and disambiguate the structure of the segmental duplications.

**Disclosure of interest**

None declared.

## O27 Mutation spectrum in a pulmonary arterial hypertension (PAH) cohort and identification of associated truncating mutations in TBX4

### C. Gonzaga-Jauregui^1^, L. Ma^2^, A. King^1^, E. Berman Rosenzweig^2,3^, U. Krishnan^2^, J. G. Reid^1^, J. D. Overton^1^, F. Dewey^1^, W. K. Chung^2,3^

#### ^1^Regeneron Genetics Center, Regeneron Pharmaceuticals, Tarrytown, New York, NY, USA; ^2^Department of Pediatrics, New York, NY, USA; ^3^Department of Medicine, Columbia University Medical Center, New York, NY, USA

##### **Correspondence:** C. Gonzaga-Jauregui – Regeneron Genetics Center, Regeneron Pharmaceuticals, Tarrytown, New York, NY, USA

**Objectives**

To identify the genetic determinants of Pulmonary Arterial Hypertension (PAH) in a cohort of pediatric PAH patients.

**Methods**

We performed whole-exome sequencing (WES) in a cohort of 60 probands with PAH and family members when available (180 total individuals) without a molecular diagnosis after most of the series was screened for mutations in *BMPR2*. In addition, we performed WES in additional 118 singleton cases. We screened all samples for variants in known PAH associated genes and performed trio-based analysis to identify novel candidate PAH genes.

**Results**

We identified known and novel mutations in the known PAH genes. In addition we identified novel truncating variants in *TBX4* occurring *de novo* or inherited from an asymptomatic parent in 5 patients and a *de novo* predicted deleterious nonsynonymous variant in one additional patient.

*TBX4* is a transcription factor of the T-box gene family. It is expressed in a variety of tissues during early mouse development including the atrium of the heart, the limbs, and the mesenchyme of the lung and trachea. TBX4, jointly with TBX5, has been shown to interact with FGF10 during lung growth and branching. Mutations in *TBX4* have been previously reported to cause small patella syndrome, an autosomal-dominant skeletal dysplasia characterized by patellar aplasia or hypoplasia. A study in 2012 [1] identified an association of *TBX4* mutations with PAH in 6 patients.

**Conclusion**

We identified 6 different deleterious variants in *TBX4* (2 inherited and 5 *de novo*) in our initial cohort of trios where patients were ascertained for primary pulmonary arterial hypertension, accounting for ~10% of the probands in this series. Subsequently we confirmed this association in additional 4 singleton cases, including a patient with a large intragenic microdeletion of *TBX4*. Our results confirm the role of *TBX4* as an important cause of hereditary PAH, accounting for ~5% of our whole PAH cohort.

**References**

1. Kerstjens-Frederikse WS, Bongers EM, Roofthooft MT, Leter EM, Douwes JM, Van Dijk A, Vonk-Noordegraaf A, Dijk-Bos KK, Hoefsloot LH, Hoendermis ES, Gille JJ, Sikkema-Raddatz B, Hofstra RM, Berger RM., TBX4 mutations (small patella syndrome) are associated with childhood-onset pulmonary arterial hypertension, J Med Genet. 2013;50(8):500–6.

**Disclosure of interest**

C. Gonzaga-Jauregui Employee of: Regeneron Pharmaceuticals, L. Ma: None declared., A. King: None declared., E. Berman Rosenzweig: None declared., U. Krishnan: None declared., J. Reid Employee of: Regeneron Pharmaceuticals, J. Overton Employee of: Regeneron Pharmaceuticals, F. Dewey Employee of: Regeneron Pharmaceuticals, W. Chung: None declared.

## O28 NORTH CAROLINA macular dystrophy (MCDR1): mutations found affecting PRDM13

### K. Small^1^, A. DeLuca^2^, F. Cremers^3^, R. A. Lewis^4^, V. Puech^5^, B. Bakall^6^, R. Silva-Garcia^1^, K. Rohrschneider^7^, M. Leys^8^, F. S. Shaya^1^, E. Stone^9^

#### ^1^Molecular Insight Research Foundation, Glendale; ^2^Ophthalmology, University of Iowa, Iowa City, IA, USA; ^3^Biology, Raboud University Medical Center, Nijmegen, Netherlands; ^4^Ophthalmology, Baylor College of Medicine, Houston, TX, USA; ^5^Service d’Exploration de la vision et Neuro-ophtalmologie CHRU, Service d’Exploration de la vision et Neuro-ophtalmologie CHRU, Lille, France; ^6^Associated Retina Consultants, University of Arizona College of Medicine, Phoenix, TX, USA; ^7^University of Heidelberg, Heidelberg, Germany; ^8^WVU Eye Institute, Morgantown, WV, USA; ^9^University of Iowa, Iowa City, IA, USA

##### **Correspondence:** K. Small – Molecular Insight Research Foundation, Glendale

**Objectives**

To identify mutations causing North Carolina macular dystrophy (NCMD, MCDR1).

**Methods**

We performed targeted Nex Gen sequencing of the MCDR1 region (870kb) in 8 affected individuals from 3 families representing 3 different haplotypes affected with chromosome 6 linked NCMD (MCDR1). In addition to our original 11 MCDR1 families recently published (141 total subjects), we now have an additional cohort of 23 families with the NCMD phenotype available for study (total of 367 subjects, 32 families).

**Results**

We initially found 14 rare variants spanning 870kb of the disease-causing allele. One of these variants (V1, ch6:1000400906) was absent from all published databases and all 261 controls, but was found in a total of 13 NCMD kindreds. This variant lies in a DNase 1 hypersensitivity site (DHS) upstream of both the PRDM13 and CCNC genes. Sanger sequencing of 1 kb centered on V1 was performed in the remaining NCMD probands, and 2 additional novel single nucleotide variants (V2, ch6:10000987, in 6 families and V3, ch6:100041040 in 1 family) were identified in the DHS within 134 bp of the location of V1. A complete duplication of the PRDM13 gene was also discovered in a single family (V4). The 4 mutations V1 to V4 segregated perfectly in the 118 affected and 33 unaffected members of the 21 NCMD families.

**Conclusion**

We identified 4 rare mutations in a non-coding region, each capable of arresting human macular development by causing over expression of PRDM13. Additional families with the NCMD phenotype continue to support that these mutations are causative of MCDR1 / NCMD.

**References**

1. Kent W. Small, *MD*, Adam P. DeLuca, PhD, Edwin. M. Stone, MD, PhD et al. North Carolina Macular Dystrophy Is Caused by Dysregulation of the Retinal Transcription Factor PRDM13Ophthalmology 2015;-:1e10 ª 2015 by the American Academy of Ophthalmology.

**Disclosure of interest**

None declared.

## O29 PhenoDB and genematcher, solving unsolved whole exome sequencing data

### N. L. Sobreira^1^, F. Schiettecatte^2^, H. Ling^3^, E. Pugh^3^, D. Witmer^3^, K. Hetrick^3^, P. Zhang^3^, K. Doheny^3^, D. Valle^1^, A. Hamosh^1^

#### ^1^Johns Hopkins University School of Medicine, Baltimore, MD, USA; ^2^FS Consulting, Salem, MA, USA; ^3^Center for Inherited Disease Research, JHUSOM, Baltimore, MD, USA

##### **Correspondence:** N. L. Sobreira – Johns Hopkins University School of Medicine, Baltimore, MD, USA

**Objectives**

To identify the causative variant(s) and gene(s) of rare Mendelian phenotypes by the re-analysis of unsolved whole exome sequecing (WES) data.

**Methods**

To address some of these cases, we have incorporated maternal and paternal imprinting analysis and polygenic analysis to the PhenoDB Variant Analysis tool. We also analyzed WES data from 1063 samples for rare, functional variants in known imprinted genes, in the genes on pseudoautosomal regions, genes that escape X-inactivation, and genes on chromosome Y. To facilitate data sharing as well as improve the search for patients or model organisms with variants in specific candidate genes we have also been adding capabilities to GeneMatcher (www.genematcher.org). In GeneMatcher there is an option to match based upon OMIM® number, genomic location and, as of October 2015, on phenotypic features. As part of the Matchmaker Exchange (MME) (http://matchmakerexchange.org/), we have also developed an API that was implemented in August 2015 and allows the GeneMatcher users to submit their data to query PhenomeCentral and/or DECIPHER. Also, as part of the MME we have been working with other matchmaker databases on the API implementation to connect them to GeneMatcher and have been working on the version 2.0 of the API that will allow for more detailed queries.

**Results**

We found that the genes in the pseudoautosomal regions are not captured by the Agilent SureSelect v4 baits that we used to sequence these samples. The analysis of variants in the genes on chromosome Y identified 52 rare functional variants and the analysis of variants in the 242 imprinted genes identified 4,337 rare functional variants. These variants are being further evaluated to define causality. As of December 2015, 3,568 genes were submitted to GeneMatcher by 984 individuals from 48 countries and 1252 matches (60 matches with PhenomeCentral and 34 matches with DECIPHER).

**Conclusion**

The GeneMatcher approach has enabled collaborations and the description of novel Mendelian phenotypes and novel Mendelian genes like *SPATA5*, *HNRNPK* and *TELO2.* We expect that further use of GeneMatcher and other MME matchmaker databases will enable many new gene/phenotype connections and that the full impact this approach will be revealed in the published literature over the next years.

**Disclosure of interest**

None declared.

## O30 Baylor-Johns Hopkins Center for Mendelian genomics: a four year review

### S. N. Jhangiani^1^, Z. Coban Akdemir^2^, M. N. Bainbridge^1^, W. Charng^2^, W. Wiszniewski^2^, T. Gambin^2^, E. Karaca^2^, Y. Bayram^2^, M. K. Eldomery^2^, J. Posey^2^, H. Doddapaneni^1^, J. Hu^1^, V. R. Sutton^2^, D. M. Muzny^1^, E. A. Boerwinkle^1^, D. Valle^3^, J. R. Lupski^2^, R. A. Gibbs^1^

#### ^1^Human Genome Sequencing Center, Baylor College of Medicine, Houston, TX, USA; ^2^Department of Molecular and Human Genetics, Baylor College of Medicine, Houston, TX, USA; ^3^McKusick-Nathans Institute of Genetic Medicine, Johns Hopkins School of Medicine, Baltimore, MD, USA

##### **Correspondence:** S. N. Jhangiani – Human Genome Sequencing Center, Baylor College of Medicine, Houston, TX, USA

**Objectives**

At its inception in 2011 the Baylor-Johns Hopkins Center for Mendelian Genomics (BHCMG), as one of three NIH funded Centers for Mendelian Genomics (CMGs), began its efforts towards: i) novel gene/mutation discovery, ii) elucidating the molecular bases of disease, iii) understanding the genetic susceptibility to disease traits, and iv) determining the genetic/genomic architecture of disease. Collectively the CMGs have sequenced ~19,000 patient samples in collaboration with more than 500 investigators from 36 countries in the past four years. The BHCMG has learned many lessons from its contribution of 5,200 exomes ranging in 475 phenotypes and presented in 82 publications.

**Methods**

The Human Genome Sequencing Center (HGSC) at the Baylor College of Medicine has generated 46 TB of whole exome sequencing (WES) data using the HGSC-VCRome capture reagent and now includes ‘Spike-in PKv2’ to capture more difficult regions and additional gene targets. The HGSC-VCRome capture reagent, along with a multiplex strategy and use of full-length blocking oligos employed for hybridization, has yielded 7.7Gb of data per exome providing a coverage of 96% at 20X or greater. The Spike-in PKv2 reagent is comprised of 3,643 additional unique gene targets derived from GeneTests, OMIM, selected cancer genes and Baylor Miraca Genetics Laboratory positive cases. The addition of this reagent converts >700-800 genes from partially covered to fully covered at ≥ 20X coverage.

**Results**

Success rates in this program have varied by phenotype and cohort collections ranging from 37% to 85%. The BHCMG has established valuable sample acquisition approaches and resources, enhanced sequencing methodology, curated a well-characterized phenotype-rich genetic database enabling genotype/phenotype relationships and encouraged collaborative efforts (i.e. GeneMatcher) to implicate 491 disease genes including 192 novel, 152 known and 147 phenotypic expansion genes.

**Conclusion**

Each discovery has shown the diagnostic capabilities in using WES and has taught lessons in disease mechanisms that continue to drive investigation into those cases that remain unsolved.

**Disclosure of interest**

None declared.

## O31 Using read overlap assembly to accurately identify structural genetic differences in an Ashkenazi Jewish Trio

### S. Shekar^1^, W. Salerno^2^, A. English^2^, A. Mangubat^1^, J. Bruestle^1^

#### ^1^Spiral Genetics, Seattle, WA, USA; ^2^Human Genome Sequencing Center, Baylor College of Medicine, Houston, TX, USA

##### **Correspondence:** S. Shekar – Spiral Genetics, Seattle, WA, USA

**Objectives**

Accurately identifying genetic differences between individuals or within samples taken from the same individual (tumor with normal control) is important to understanding the etiology of diseases, particularly for disease areas where large structural changes in the genome have been associated with the disease, such as neurological conditions, cardiological conditions and cancer. In clinical practice, identifying a previously uncharacterized de novo SV in an offspring that could be causing a condition is challenging with current methods that often have high false discovery rates.

**Methods**

Here, we present Biograph Anchored Assembly (BAA), an SV caller using whole read overlap assembly of reads that do match the reference exactly. In a previous study with next-generation sequencing of the reference individual HS1011 (English et al. (2015)), the method has been shown to have high sensitivity compared to other SV callers and a false discovery rate of less than 5%. The method is based upon the BioGraph data storage format (BAF). The BAF is a specialized index of the read overlap graph of a genome that can be queried up to one million times a second. Querying by both coordinate and sequence is particularly applicable to SV typing.

**Results**

Using BAA on a trio sequenced by the Personal Genome Project (PGP), the BAA variant caller detected a 3.4kb insertion inherited in the offspring that matched an alternate allele assembly now in GRCh38. The breakpoint and sequence of the insertion were reported. The resolution of this inserted sequence allowed for five SNPs and an indel that were inherited from the father and a single SNP inherited from the mother to be distinguished in the offspring.

**Conclusion**

This level of resolution and accuracy in calling allows for structural variants, and even differences between structural variants, to be compared across individuals. This is important both for understanding the etiology of disease in larger studies as well as identifying de novo variants in an offspring in a clinical setting. Here, we further present results from 100 HiSeq X samples sequenced at 30x, including multiple classes of structural variants and multi-sample classification of shared breakpoints.

**References**

English AC, Salerno WJ, Hampton OA, et al. Assessing structural variation in a personal genome-towards a human reference diploid genome. BMC Genomics. 2015; 16: 286.

**Disclosure of interest**

None declared.

## O32 Legal interoperability: a sine qua non for international data sharing

### A. Thorogood, B. M. Knoppers, Global Alliance for Genomics and Health - Regulatory and Ethics Working Group

#### Centre of Genomics and Policy, McGill University, Montreal, Quebec, Canada

##### **Correspondence:** A. Thorogood – Centre of Genomics and Policy, McGill University, Montreal, Quebec, Canada

**Objectives**

The successful translation of genomic discovery into improvements in human health hinges on the effective and responsible sharing of genomic and health related data. The Global Alliance for Genomics and Health (GA4GH) brings together over 375 leading healthcare, research, disease advocacy, life science, and information technology organizations committed to establishing harmonized approaches to data sharing. The Regulatory and Ethics Working Group (REWG) of the GA4GH specifically promotes the legal interoperability of data.

**Methods**

The REWG establishes Task Teams with diverse, international membership across sectors to develop tools and solutions to facilitate data sharing. Existing Task Teams address Consent, Privacy & Security, Accountability, Paediatrics, Ageing and Dementia, Registered Access, Participant Values, and Machine Readable Consent.

**Results**

The REWG has developed a *Framework for Responsible Sharing of Genomic and Health-Related Data*, which aims to activate the human right of every citizen to benefit from the progress of science. This human rights basis gives the *Framework* political and legal dimensions that reach beyond the moral appeals of bioethics. Specific policies build on the *Framework*. The Consent policy provides guidance for balancing individual autonomy with the public good, and addresses sharing of legacy data where consent is silent on data sharing. The Privacy and Security policy emphasizes that safeguards should be proportionate to the risk of harm to patients. The new Accountability Policy provides best practices for transparency of data handling, as well as processes for monitoring and responding to non-compliance with data sharing standards.

**Conclusion**

In addition to these policies, ongoing initiatives within the REWG are tackling a host of related questions. What language is appropriate for consent to broad data sharing? Is there a middle ground between open and controlled access? Can conditions of data access be rendered machine readable, so as to enable automated data discovery? Can researchers be held accountable for *not sharing* data where patients have consented to broad sharing? And how do patients and the public feel about sharing their data for broad research purposes?

**References**

Knoppers BM. Framework for responsible sharing of genomic and health-related data. The HUGO Journal. 2014;8(1):3.

Knoppers BM. International ethics harmonization and the global alliance for genomics and health. Genome Med. 2014;6:13.

**Disclosure of interest**

None declared.

## O33 High throughput screening platform of competent sineups: that can enhance translation activities of therapeutic target

### H. Takahashi^1^, K. R. Nitta^1^, A. Kozhuharova^1^, A. M. Suzuki^1^, H. Sharma^1^, D. Cotella^2^, C. Santoro^2^, S. Zucchelli^3^, S. Gustincich^3^, P. Carninci^1^

#### ^1^Center for Life Science Technologies, Division of Genomic Technologies, RIKEN, Yokohama, Japan; ^2^Dipartimento di Scienze della Salute, Universita’ del Piemonte Orientale, Novara, Italy; ^3^Area of Neuroscience, SISSA, International School for Advanced Studies, Trieste, Italy

##### **Correspondence:** H. Takahashi – Center for Life Science Technologies, Division of Genomic Technologies, RIKEN, Yokohama, Japan

**Objectives**

Short interspersed elements B2 (SINE B2) are broadly distributed transposable elements in whole mouse genome. Evolutionally, SINE B2s share common ancestors with tRNAs. Among the various remarkable functions of recently discovered non-coding RNA functions, we discovered a new class of antisense non-coding RNAs (SINEUPs) that promote translation of partially overlapping sense coding mRNAs with no effects on RNA levels.. In order to develop synthetic SINEUPs to up-regulate therapeutically interesting genes, we have determined that SINEUPs function requires two essential domains; one is SINE B2 element, also called the Effector Domain (ED), and the other is overlapping antisense RNA sequence, which provide specificity and is called the Binding Domain (BD). We have produced functional SINEUPs for to a PD associate gene, PARK7 (DJ-1), as well as other genes. This synthetic SINEUP specifically enhances translation level of PARK7 mRNAs in human neuronal cell lines. Through a novel high-throughput screening (HTS) system, we aim at further optimization of the ED and BD of SINEUPs to produce very effective SINEUPs against any possible mammalian protein.

**Methods**

We report here our HTS, which is based on high-resolution automated fluorescent imaging of CeligoS instrument. We screened with the HTS several BDs for a hepatic transcription factor alpha (Hnf4-alpha), which is associated to maturity-onset diabetes of the young type 1.

**Results**

In addition, we validated that several SINEUPs targeting Hnf4-alpha are able to upregulate translation in mouse hepatoma cells and hepatocyte cells. We also validated that other EDs derived from natural SINE B2 sequences revealed target mRNA specific translation enhancement.

**Conclusion**

To conclude, synthetic SINEUPs are promising tools for gene/RNA therapy of haploinsufficiencies, and the HTS system is a powerful SINEUPs screening platform.

**Disclosure of interest**

None declared.

## O34 The undiagnosed diseases network international (UDNI): clinical and laboratory research to meet patient needs

### J. J. Mulvihill^1^, G. Baynam^2^, W. Gahl^3^, S. C. Groft^4^, K. Kosaki^5^, P. Lasko^6^, B. Melegh^7^, D. Taruscio^8^

#### ^1^Division of Genomic Medicine, National Human Genome Research Institute, Bethesda, MD, USA; ^2^Office of Population Health, Department of Health, Perth, Australia; ^3^Undiagnosed Diseases Program, National Human Genome Research Institute, Bethesda, MD, USA; ^4^National Center for Advancing Translational Sciences, National Institutes of Health, Bethesda, MD, USA; ^5^Center for Medical Genetics, Keio University School of Medicine, Tokyo, Japan; ^6^Department of Biology, McGill University, Montreal, Quebec, Canada; ^7^Department of Medical Genetics, University of Pecs, Pecs, Hungary; ^8^National Center for Rare Diseases, Istituto Superiore di Sanita, Rome, Italy

##### **Correspondence:** J. J. Mulvihill – Division of Genomic Medicine, National Human Genome Research Institute, Bethesda, MD, USA

**Objectives**

Rare and undiagnosed disorders challenge patients, families, and clinicians. In 2008, NIH started an Undiagnosed Diseases Program (UDP), with the goals of providing answers to patients with mysterious conditions that eluded diagnosis and advancing medical knowledge about diseases. The UDP has expanded in the US, as the Undiagnosed Diseases Network, with 6 additional clinical sites, a coordinating center, 2 DNA sequencing cores, a model organisms screening center, a metabolomics core, and a biorepository. For further expansion, meetings were held in Rome and Budapest with clinician scientists from 7 nations.

**Methods**

The plan includes launching the UDNI (Australia, Canada, Hungary, Italy, Japan, Sweden, and the United States). The goals are to improve the level of diagnoses and care for such patients by common protocols, to facilitate research in disease etiology, and to create a collaborative research community. A comprehensive “–omics” approach would include exomic and genomic sequencing as well as metabolomics. The interim website is http://test.areasrl.com/udni/home.

**Results**

To date, several principles are being implemented: Engaging centers of excellence, fostering a collaborative research environment, establishing a cooperative governance structure, designing a common research protocol, providing a uniform patient experience, collecting data by recognized standards, protecting patient data, observing ethical, legal, and social guidelines, devising broad data sharing, stimulating dissemination of results, and ensuring a well-functioning network. A Board of Directors integrates working committees, including Patient Advocacy, Clinical Management, Sequencing, Databases, and Repositories.

**Conclusion**

Further meetings should consolidate support and logistics. Details are available (Taruscio E, et al., Undiagnosed Diseases Network International (UDNI): White Paper for Global Actions to Meet Patient Needs (*Mol Genet Metabol,* in press).

**Disclosure of interest**

None declared.

## O36 Performance of computational algorithms in pathogenicity predictions for activating variants in oncogenes versus loss of function mutations in tumor suppressor genes

### R. Ghosh, S. Plon

#### Pediatrics-Oncology, Baylor College of Medicine, Houston, TX, USA

##### **Correspondence:** R. Ghosh – Pediatrics-Oncology, Baylor College of Medicine, Houston, TX, USA

**Objectives**

Several computational methods have been developed to predict whether amino acid substitutions result in disease. This type of analysis is included in the ACMG/AMP guidelines for pathogenicity classification of variants and is being used by the Clinical Genomics (ClinGen) resource. These methods are generally blind to the underlying disease mechanism. Little is known about how mechanism of disease affects the predictive ability of these algorithms for variants implicated in inherited diseases. We address this by focusing on two classes of genes that differ in their molecular mechanism of action. Activating/gain-of-function mutations in oncogenes and loss-of-function mutations in tumor-sppressor genes(TSG) are pathogenic in cancer development. Moreover, unlike TSG, oncogenes are recurrently mutated at several amino acid positions.

**Methods**

We obtained 5078 missense variants in 29 oncogenes and 50 TSG, classified based on their pattern of mutations in COSMIC(1), from the ClinVar database and annotated them with 20 computational algorithms. These variants had clinical assertions provided by the submitting laboratory. We analyzed variants classified as either pathogenic or benign in oncogenes (n=321) and TSG (n=832).

**Results**

We found less concordance among the algorithms assessed for pathogenic variant prediction in either class of genes. Also the set of algorithms that were concordant in predicting benign and pathogenic variants differed on whether the variant was an oncogene or TSG. The concordant (e.g. GERP++) algorithms are primarily based on evolutionary conservation. A combination of GERP++ and the functional prediction algorithm FATHMM is more likely to produce discordant results for oncogenes. This implies that curators choosing different sets of computational algorithm are likely to result in different inferences for the same variants. We are developing statistical approaches to identify algorithms that produces maximal separation of benign and pathogenic variants for oncogenes and TSGs and applying them in a larger set of variants in the list of 56 genes recommended for reporting for incidental findings differing in their disease mechanism.

**Conclusion**

We find evidence that disease mechanism needs to be taken into consideration when deciding on algorithms for predicting pathogenicity. Our findings may aid in further classification of variants of uncertain significance.

**References**

1. Vogelstein B, et al. Cancer genome landscapes. Science. 2013; 339(6127):1546–1558.

**Disclosure of interest**

None declared.

## O37 Identification and electronic health record incorporation of clinically actionable pharmacogenomic variants using prospective targeted sequencing

### S. Scherer^1^, X. Qin^1^, R. Sanghvi^1^, K. Walker^1^, T. Chiang^1^, D. Muzny^1^, L. Wang^2^, J. Black^3^, E. Boerwinkle^1^, R. Weinshilboum^4^, R. Gibbs^1^

#### ^1^Human Genome Sequencing Center, Baylor College of Medicine, Houston, TX, USA; ^2^Department of Molecular Pharmacology and Experimental Therapeutics, Mayo Clinic, Rochester, NY, USA; ^3^Department of Psychiatry, Mayo Clinic, Rochester, NY, USA; ^4^Department of Pharmacology, Mayo Clinic, Rochester, NY, USA

##### **Correspondence:** S. Scherer – Human Genome Sequencing Center, Baylor College of Medicine, Houston, TX, USA

**Objectives**

The Baylor College of Medicine’s Human Genome Sequencing Center and the Mayo Clinic’s Center for Individualized Medicine are collaboratively working to sequence up to 10,000 patients from the Mayo Clinic Biobank. The objective of these efforts is to incorporate the results in patient electronic health records (EHRs) thus guiding clinical drug prescribing practices in terms of efficacy and avoidance of adverse events.

**Methods**

This study takes a prospective approach using a combination of reagents including a targeted panel of seventy-six pharmacogenomically relevant genes developed as part of the Pharmacogenomic Research Network (PGRN). Specific targets are based primarily on a combination of community feedback and clinical guidelines published by the PGRN’s Clinical Pharmacogenetics Implementation Consortium (CPIC). The targets include both gene coding regions and SNP targets aimed at characterizing both known and novel variants while keeping the costs equal to or below microarray-based genotyping approaches.

**Results**

Preliminary data was generated using 500 samples used previously as part of the Mayo Clinic’s eMERGE Network studies. Each institution developed both data generation and analysis pipelines geared toward identification of genomic variants and haplotypes influencing commonly prescribed drug efficacies and toxicities. Preliminary sequencing data quality was outstanding and demonstrated high variant correlation with the previous dataset. Improvements in haplotype calling and clinical decision support are ongoing. Moving forward, the Mayo Clinic’s Center for the Science of Health Care Delivery will be tracking outcomes to confirm the value of this approach.

**Conclusion**

In conclusion, this study will provide a large cohort blueprint for implementation of pharmacogenomics in precision individualized health care.

**Disclosure of interest**

None declared.

## O38 Melanoma reprogramming state correlates with response to CTLA-4 blockade in metastatic melanoma

### T. Karpinets, T. Calderone, K. Wani, X. Yu, C. Creasy, C. Haymaker, M. Forget, V. Nanda, J. Roszik, J. Wargo, L. Haydu, X. Song, A. Lazar, J. Gershenwald, M. Davies, C. Bernatchez, J. Zhang, A. Futreal, S. Woodman

#### MD Anderson Cancer Center, Houston, USA

##### **Correspondence:** T. Karpinets – MD Anderson Cancer Center, Houston, USA

**Objectives**

Targeting immune checkpoints has proven to be an effective strategy for the treatment of metastatic melanomas. However, less than half of patients respond to the immune checkpoint blockade. A complete understanding of molecular mechanisms underlying tumor response is lacking. In this study, we propose that the degree of melanoma cell “re-programming” may contribute to melanoma tumor resistance to immune therapy.

**Methods**

We employ RNA-sequencing (RNA-seq) and Reverse Phase Protein Array (RPPA) data from 68 early passage melanoma cell lines derived from tumor infiltrating lymphocyte harvests of 63 patients to identify biological processes and marker genes underlying the melanocyte “re-programming”. We propose a scoring system of the process based and employ it to study effects of the re-programming on outcomes of the immune therapy using a transcriptomics dataset (Van Allen et all, 2015) from pretreatment metastatic melanoma tumor samples from patients treated with ipilimumab (anti-CTLA-4).

**Results**

**Fig. 5 (abstract O38).**
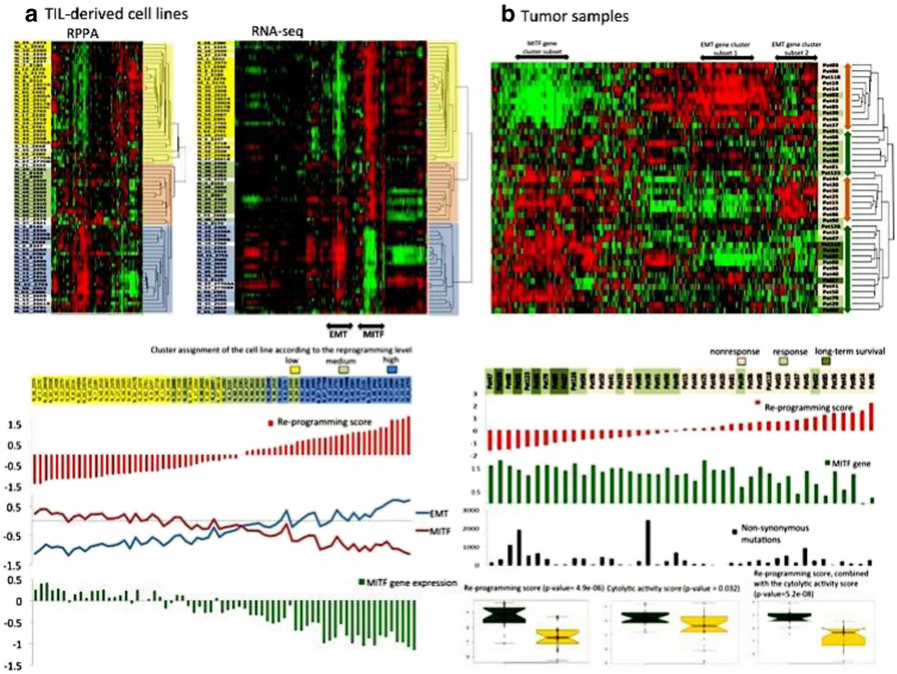
ᅟ

Melanoma cells grouped into 3 major concordant clusters by both RNA-seq and RPPA analysis (Fig. 5). Examination of the genes underlying the clustering revealed profound differences in the expression of genes associated with melanocyte differentiation (including MITF) and with the Epithelial to Mesenchymal Transition (EMT) process. We determined the mean Z value for genes within each process, and designated the difference between the mean expressions as the “re-programming score” (RPS). Using the same set of marker genes for melanoma tumor samples we significantly separated responders from non-responders of the immune therapy and revealed 2 groups of non-responding tumors. Each group had a different subset of highly expressed EMT-associated genes, and opposite expression of the differentiation-associated genes. Combining a subset of genes that are differentially expressed between responders and non-responders we markedly enhanced the prognostic value of the cytolytic score, a known prognostic feature.

**Conclusion**

The proposed scoring system of the melanocyte re-programming based on the RPS may hold prognostic value for immunotherapy treatments outcomes.

**Disclosure of interest**

None declared.

## O39 Data-driven refinement of complex disease classification from integration of heterogeneous functional genomics data in GeneWeaver

### E. J. Chesler^1^, T. Reynolds^2^, J. A. Bubier^1^, C. Phillips^3^, M. A. Langston^3^, E. J. Baker^2^

#### ^1^The Jackson Laboratory, Bar Harbor, ME, USA; ^2^Baylor University, Waco, TX, USA; ^3^University of Tennessee, Knoxville, TN, USA

##### **Correspondence:** E. J. Chesler – The Jackson Laboratory, Bar Harbor, ME, USA

**Objectives**

Challenges in research, diagnosis and treatment of complex disease emerge from the poor alignment of the underlying biology of disease with a nosology defined by externally manifest signs and symptoms. Aggregate functional genomics data can enable the development of a data driven nosology in which disease characteristics, research models, diagnostic categories and drugs are more precisely aligned to specific, biologically based facets of disease.

**Methods**

GeneWeaver consists of a database and analysis tools for aggregation of heterogeneous functional genomics data across species, including curated pathways, ontology annotations, publication data, genetic mapping, transcriptome and proteome experiments, and other functional genomics data including user-submitted experimental results. Each is described with meta-content, enabling retrieval by disease related terms. Gene identifiers are harmonized to enable aggregation of data through homologous genes and gene products. To evaluate specificity of disease descriptors, we analyzed intersections among gene sets associated with disease-related terms. These gene sets were derived from studies of nine different organisms and consisted of genome-wide experiments, curated annotations to ontology terms and genes associated to disease-related terms through transitive association of genes, publications and MeSH terms.

**Results**

Analyses of term-to-term associations reveals that genes associated to co-occurring or difficult to discern diseases exhibit weak overlap across many different terms, especially those associated with psychiatric disorders which display extensive cross-disorder overlap. In contrast, more well-bounded disorders, such as degenerative ocular disorders, reveal strong and specific overlap and good matching of empirically derived data sets and annotations. By enumerating all intersecting associations of genes to disorders, we are simultaneously able to identify genes that differentiate among overlapping disorders, potentially defining the specific and unique aspects of these conditions for precise differentiation of disease.

**Conclusion**

The integration of heterogeneous functional genomics data provides insight into the latent biological basis underlying the organization of heterogeneous disease.

Supported by NIH AA18776, jointly funded by NIAAA and NIDA.

**Disclosure of interest**

None declared.

## O40 A general statistic framework for genome-based disease risk prediction

### M. Xiong^1^, L. Ma^1^, N. Lin^1^, C. Amos^2^

#### ^1^University of Texas School of Public Health, Houston, TX, USA; ^2^Geisel School of Medicine at Dartmouth, Hanover, NH, USA

##### **Correspondence:** M. Xiong – University of Texas School of Public Health, Houston, TX, USA

**Objectives**

How to efficiently extract biomarkers for risk prediction and treatment selection from millions or dozens of millions of genomic variants raises a great challenge. Traditional paradigms for identifying variants of clinical validity are to test association of the variants. However, significantly associated genetic variants may or may not be efficient for diagnosis and prognosis of diseases. Alternative to association studies for finding genetic variants of predictive utility is to systematically search variants that contain sufficient information for phenotype prediction.

**Methods**

To achieve the goal, we introduce concepts of sufficient dimension reduction (SDR) which project the original high dimensional data to very low dimensional space while preserving all information on response phenotypes. We then formulate a clinically significant genetic variant discovery problem into the sparse SDR and optimal scoring problem and develop algorithms that can select significant genetic variants from high dimensional data. To speed up computation, we apply the alternating direction method of multipliers to solving the sparse optimal scoring problem which can easily be implemented in parallel.

**Results**

To illustrate its application, the proposed method is applied to a coronary artery disease (CAD) dataset from the Wellcome Trust Case Control Consortium (WTCCC) study, Rheumatoid Arthritis (RA) dataset from the GWAS of North American Rheumatoid Arthritis Consortium (NARAC) and the early-onset myocardial infarction (EOMI) exome sequence datasets which have European origin from the NHLBI’s Exome Sequencing Project. To evaluate the performance of the SDR for disease risk prediction, we present Table 1 that lists AUC of our SDR and other 10 existing methods. Table 1 clearly demonstrated that our proposed SDR method has much larger AUC than other 10 existing methods.

**Conclusion**

We shift the paradigm of feature selection from P-value and risk score ranking to optimal genome-wide searching. The SDR-based optimal genome-wide searching methods substantially outperform other existing methods for disease risk prediction. Our results strongly demonstrate that the rich genetic variation information provides powerful resources for disease risk prediction.

**Disclosure of interest**

None declared.

**Table 1 (abstract O40):** ᅟ

Disease	BLUP	GRC	BSLMM	MultiBLUP	Elastic Net	Lasso	Disease	BLUP	GRC	BSLMM	MultiBLUP
CAD	0.58	0.57	0.59	0.59	0.683	0.678	CAD	0.58	0.57	0.59	0.59
RA	0.61	0.67	0.69	0.73	0.765	0.766	RA	0.61	0.67	0.69	0.73

## O41 Integrative large-scale causal network analysis of imaging and genomic data and its application in schizophrenia studies

### N. Lin^1^, P. Wang^1^, Y. Zhu^2^, J. Zhao^2^, V. Calhoun^3^, M. Xiong^4^

#### ^1^Biostatistics, University of Texas Health Science Center at Houston, Houston, TX, USA; ^2^Tulane University, New Orleans, LO, USA; ^3^University of New Mexico, Albuquerque, NM, USA; ^4^University of Texas Health Science Center at Houston, Houston, TX, USA

##### **Correspondence:** N. Lin – Biostatistics, University of Texas Health Science Center at Houston, Houston, TX, USA

**Objectives**

Next generation genomic and image technologies produce a deluge of DNA sequencing, transcriptomes, metabolic, image, physiological phenotypes with millions of features. Analysis of increasingly larger and more complex data gives scientists access to vast amounts of information that was previously unavailable, but also poses great methodological and computational challenges. This talk provides perspectives for paradigm changes in current public health data analysis.

**Methods**

We develop novel statistical methods for paradigm changes in big genomic, epigenomic and imaging data analysis from low dimensional data to high dimensional data analysis. We develop novel functional structural equations with integer programming as a new framework for inferring large-scale causal networks of genomic-images and detecting pleiotropic effects of genetic variants on imaging. In addition, we develop new causal machine learning methods for network classification and combine images and genomic data for disease risk prediction.

**Results**

The proposed method for large-scale genomic-imaging causal network analysis was applied to the MIND clinical imaging consortium’s schizophrenia image-genetic study with 142 diffusion tensor images (DTI) with 538265 voxels and 14,412 genes in 64 schizophrenia patients and 78 controls. Each DTI were segmented into 41 regions. The causal image-genotype networks were constructed for all the individuals. In cases, the image network consisted of 41 nodes and 68 edges, and in controls, the image network consisted of 41 nodes and 65 edges. We identified 1,035 and 1,618 genes that were significantly connected to the image regions respectively. 27 genes in cases and 40 genes in controls were in the 108 schizophrenia associated genetic loci. The developed network classification algorithm was also applied to predict schizophrenia. Using cross validation, we can achieve 100% prediction accuracy in the training data and the average prediction accuracy, sensitivity and specificity in the test data were 95.1%, 96.2% and 93.9% respectively.

**Conclusion**

We shift the paradigm of big genomic and imaging data analysis from association studies to causal inference and provide powerful tools for unravelling causal chain of mechanisms of psychiatric disorders, delivering new therapeutic targets and biomarkers for precision medicine

**Disclosure of interest**

None declared.

## O42 Big data and NGS data analysis: the cloud to the rescue

### O. Dobretsberger, M. Egger, F. Leimgruber

#### EPS Software Corp, Spring, TX, USA

##### **Correspondence:** O. Dobretsberger – EPS Software Corp, Spring, TX, USA

**Objectives**

In the wake of the development of new Next Generation Sequencing (NGS) instruments and methodologies, genetic research has become more prominent during the past several years. However, many laboratories and institutions still face significant issues in their modus operandi: Storage, processing, and sharing of NGS data and results. We propose a novel approach to overcome mentioned issues: Wikinome, a cloud based platform specifically designed and developed to deal with the issues typical bioinformatics laboratories are confronted with, allowing faster and more efficient ways to conduct NGS data analysis.

**Methods**

Wikinome allows its users to store, manipulate, analyze and share their data and results from anywhere, using desktop computers, laptops, or even mobile devices, without the need of maintaining a high powered computer in the lab. Once the NGS data are uploaded, it can be analyzed using various pipelines that can be planned and executed using any of Wikinome’s clients. Utilizing a Service oriented Architecture (WCF), all steps of an analysis workflow are called individually to perform the underlying analysis process, such as reads-mapping, reads-clustering, or performing a BLAST search. Access-controlled files in a Big Data Storage environment allow users to share data with collaborators at the ease of a button click, instead of physically or digitally moving data.

**Results**

We have successfully implemented a platform allowing all people and institutions that conduct genetic sequence analysis, to completely move their analysis procedures into the cloud, independent from the sequencing instruments that were used to produce the NGS data. Utilizing standard modules such as quality control, reference-mapping, gene-detection, de-novo assembly, alignment, and BLAST, Wikinome not only allows users to define custom analysis workflows; users can even add services hosted inside their own labs, without the need of exposing them in the cloud. Instead of working with command-line based algorithms and tools, users are automatically notified upon finishing certain procedures of the currently running workflow, and have access to live updates. Users can also add their own custom analysis modules and share them with collaborators within the Wikinome network.

**Conclusion**

With Wikinome, we have successfully developed a solution to perform analysis on NGS data on a previously unthinkable scale with the potential to overcome many of the typical Big Data issues in the field of genetic research and analysis.

**Disclosure of interest**

None declared.

## O43 Cpipe: a convergent clinical exome pipeline specialised for targeted sequencing

### S. Sadedin, A. Oshlack, Melbourne Genomics Health Alliance

#### Bioinformatics, Murdoch Childrens Research Institute, Parkville, Australia

##### **Correspondence:** S. Sadedin – Bioinformatics, Murdoch Childrens Research Institute, Parkville, Australia

**Objectives**

Efforts to move high throughput sequencing into the clinic must confront many challenges including meeting clinical standards for cost, reproducibility, quality, ethical and privacy considerations. The Melbourne Genomics Health Alliance was formed from a diverse group of institutions with the aim of sharing the burden of these challenges through a common sequencing and bioinformatics platform. In this work, we present a shared, open source analysis pipeline that was developed to meet these needs.

**Methods**

To enable a single solution for many different diseases and laboratories, we employed the Bpipe pipeline platform which allows for analysis stages to be easily added, substituted, replaced or customized on a per-sample or per-disease basis. Bpipe also offers powerful features for parallelization, so that pipelines can easily run on different distributed clusters or on dedicated computing resources. To ensure that the solution can be freely shared, it is released under the GPLv3 open source license.

**Results**

We have developed Cpipe, a shared, open source sequencing pipeline designed for clinical users. Cpipe includes support for the full range of features required in real world clinical sequencing applications. It allows for customized sets of targeted regions, prioritized genes, screening of incidental findings, automatic exclusion of sequencing artefacts and population variants, PDF provenance and quality reports. Cpipe produces a clinically interpretable report available in Excel format. Results can be optionally imported into an LOVD (Leiden Open Variant Database) instance for curation. Cpipe is available at http://cpipeline.org.

**Conclusion**

Cpipe offers an open source solution that any clinical laboratory can employ, either as their main analysis pipeline or as a benchmark for comparison to improve their results.

**References**

Sadedin SP, Pope B, Oshlack A. Bpipe: a tool for running and managing bioinformatics pipelines. Bioinformatics. 2012;28(11):1525–1526.

Sadedin SP, Dashnow H, James PA, Bahlo M, Bauer DC, Lonie A, Lunke S, Macciocca I, Ross JP, Siemering KR, Stark Z, White SM, Taylor G, Gaff C, Oshlack A, Thorne NP: Cpipe: a shared variant detection pipeline designed for diagnostic settings. Genome Med 2015, 7:68.

**Disclosure of interest**

None declared.

## O44 A Bayesian classification of biomedical images using feature extraction from deep neural networks implemented on lung cancer data

### V. A. A. Antonio, N. Ono, Clark Kendrick C. Go

#### Computational Systems Biology Laboratory, Nara Institute of Science and Technology, Ikoma-cho, Japan

##### **Correspondence:** V. A. A. Antonio – Computational Systems Biology Laboratory, Nara Institute of Science and Technology, Ikoma-cho, Japan

**Objectives**

This project aims the formulated algorithm will be implemented explicitly on lung cancer pathological images. Specifically, this project has two goals. First, it aims to apply the concept of deep neural networks to supervised learning in the classification of images, with the understanding that modifying existing machine learning methods to target specific image sets can optimize the precision and accuracy of the analysis. The algorithm that will be formulated will be able to sort a given set of data (in this case, images) into desired sets with given qualifications. Second, it aims to apply the concept of deep neural networks, supplemented by Bayesian networks to pattern analysis of lung cancer data sets. Performing Bayesian network procedures on given lung cancer data can help us determine parameter values that will characterize those data. In turn, those parameters can be used to infer whether or not new data can be classified with the given training data or otherwise.

**Methods**

First, we take tiles of size 512x512 from images published on the cancer genome atlas database (http://cancergenome.nih.gov). Several samples from those 512x512 will be segmented further into 32x32 subregions, which will be used as an example training dataset. We then incorporate the Sobel operator feature detection method, along with the standard RGB image histogram to serve as the main features for classification. These features will be extracted through the notion of deep neural networks. The algorithm will then return a classification of the subregions into either normal or abnormal, by virtue of a Bayesian classification scheme.

**Results**

3,233 tiles of size 512x512 were gathered from two whole-slide lung cancer images. Samples from these tiles were segmented further into 32x32 subregions, which served as training data for the algorithm. The main output will be an algorithm embedded in a web application wherein the user can just input newly acquired images, and the program can provide classify which 32x32 subregions depict cancer cells. A demonstration of the application can be shown during the presentation.

**Conclusion**

An executable program, whose input is a lung cancer image, will be segmented into 32x32 subregions, each of whose features will be extracted using deep neural networks and will undergo a Bayes’ classification scheme, and determine whether the subregion is normal or abnormal, either because of a broken cell, a stained cell, or an actual cancer cell.

**Disclosure of interest**

V. A. Antonio Grant/Research Support from: CICP Research Project (https://cicp.naist.jp/ja/node/85), N. Ono: None declared.

## O45 MAV-SEQ: an interactive platform for the Management, Analysis, and Visualization of sequence data

### Z. Ahmed, M. Bolisetty, S. Zeeshan, E. Anguiano, D. Ucar

#### The Jackson Laboratory for Genomic Medicine, Farmington, CT, USA

##### **Correspondence:** Z. Ahmed – The Jackson Laboratory For Genomic Medicine, Farmington, CT, USA

**Objectives**

The increasing amount of heterogeneous genomic datasets generated today necessitates a robust platform for efficient data management and analysis. To address this need, we developed a software for the **M**anagement, **A**nalysis, and **V**isualization of **seq**uence data (MAV-seq), capable of addressing the issues related to the exponential growth of genomic applications and their datasets of enormous size and diversity. Our software also integrates various genomic pre-processing pipelines with user-friendly graphical interface to enable biologist with no programming experience conduct complex data analyses.

**Methods**

MAV-seq (Figure) is a desktop application developed to integrate bioinformatics methods, software engineering principles, human computer interaction guidelines and big data analytics. The graphical user interface (GUI) and back end development of MAV-seq is performed in Java and Python, and schemas for backend data storage are implemented in MySQL and MariaDB relational database management systems. MAV-seq allows direct data manipulation using GUI as well as data import and export in “csv” file formats.

**Results:** We developed MAV-seq as an interactive, user friendly, cross platform, encrypted and multi-roles based system for the management of sample repertoires and automation of the data pre-processing of epigenomic and transcriptomic data. It supports users in performing downstream data analysis by integrating several analysis pipelines for diverse data sets including ATAC-seq, mRNA-seq, tRNA-seq, Chip-seq, WES, WGS. MAV-seq can be customized for increasingly large scaled and complex datasets of different types. Moreover, it can directly interact with multiple data clusters to locate, input and process genomics data by automatically generating and running multiple-sequential and parallel pipelines.

**Conclusion**

MAV-seq is a comprehensive data management and analyses platform that is newly designed, developed, tested, validated and deployed at The Jackson Laboratory for Genomic Medicine. With this platform, we aim to advance genome-wide big data management, standardization and automation, which will expedite the pace and improve the levels of efficiency in loading, handling, tracking, securing, sharing, processing, analyzing and visualizing data.

**Acknowledgements**

Funding for research and development is acknowledged for Project: “Research Oriented Data Management and Analysis” at the Ucar Lab by The Jackson Laboratory, USA.

**Disclosure of interest**

None declared.

**Fig. 6 (abstract O45).**
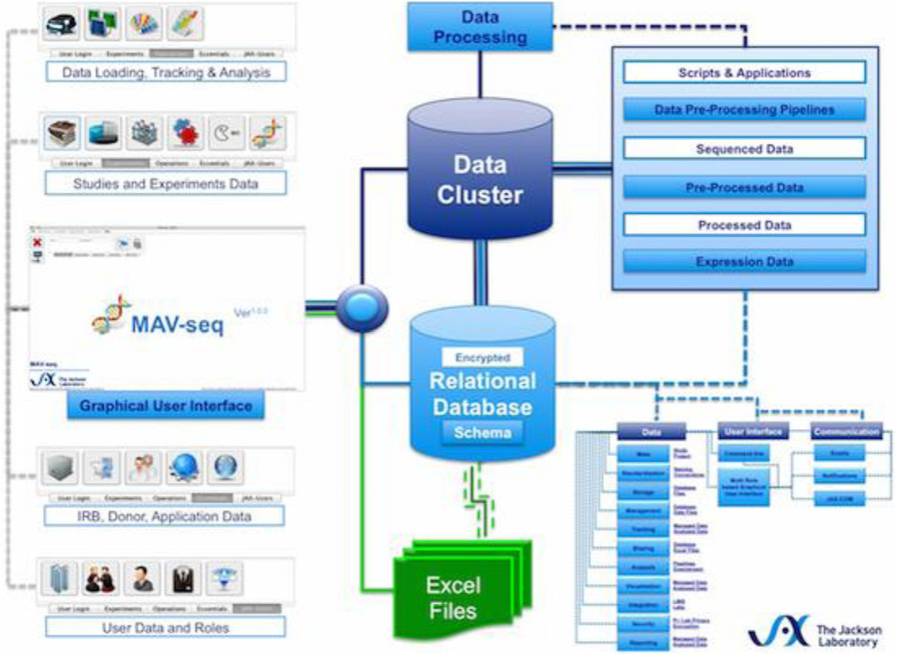
ᅟ

## O47 Allele specific enhancer in EPAS1 intronic regions may contribute to high altitude adaptation of Tibetans

### C. Zeng, J. Shao

#### Beijing Institute of Genomics, Chinese Academy of Sciences, Beijing, China

##### **Correspondence:** C. Zeng – Beijing Institute of Genomics, Chinese Academy of Sciences, Beijing, China

**Objectives**

Tibetans have shown their physiological phenotypes adaptive to hypoxia environment, including relative low hemoglobin concentration and higher oxygen saturation. Among tens of candidate genes revealed by various studies that relate to living in high altitude, *EPAS1* and *EGLN1* showed strongest selection signals as well as solid correlation to lower hemoglobin concentration in Tibetans. However, several tens of Tibetan specific SNPs in *EPAS1* were all found in introns and the mechanism of this important gene contributing to genetic adaptation in Qinghai-Tibet Plateau is still elusive. This study aims at the function of Tibetan specific SNPs in *EPAS1*.

**Methods**

1) 82 candidate genes were designed for capture and sequencing in 45 Tibetans samples to discover more SNPs for further association and functional studies. 2) Statistical tests including Chi-square tests, haplotype association, and integrative Haplotype Score, and Refseq and NONCODE were used for annotation. 3) Cell culture of HepG2 and HEK293 under normoxia and hypoxia (1%) for all functional assays. 4) Enhancer validation in a dual-luciferase reporter system. 5) Circular chromosome conformation capture (4C) in HepG2 followed by sequencing in Hi-Seq 2000.

**Results**

By re-sequencing 82 genes selected from previous studies and our recent discoveries, totally 9947 SNPs were revealed in 45 Tibetan individuals (15 from Bomi; 20 from Anduo; 10 from Dingri). Six genes were consistently identified by three statistical tests. In addition to *EPAS1* and *EGLN1*, studies on quantitative traits identified *DISC1* and *EDAR* in association with diameter of Main Pulmonary Artery and SaO2, respectively. By integrating enhancer annotation data from FANTOM5 and ENCODE, 12 *EPAS1* intronic regions were resulted. Further validation for the enhancer function demonstrated that three of them are true enhancers. Moreover, enhancer 1 showed an allele specific activity in both HepG2 and HEK293 cell lines. Especially, two Tibetan-Han divergent SNPs showed the addictive effect in enhancer activity. Further 4C-sequncing results of Enhancer 1 demonstrated the similar chromosomal conformation structures in most enhanced regions under hypoxia and normoxia treatments but a few interactions were shown to be hypoxia sensitive. Annotation with Refseq and NONCODE database revealed that targets potentially regulated by Enhancer 1 were mostly non-coding RNAs.

**Conclusion**

An allele-specific enhancer in *EPAS1* intronic region may contribute to high altitude adaptation in Tibetans.

**Disclosure of interest**

None declared.

## O48 Nanochannel based next-generation mapping for structural variation detection and comparison in trios and populations

### H. Cao, A. Hastie, A. W. Pang, E. T. Lam, T. Liang, K. Pham, M. Saghbini, Z. Dzakula

#### BioNano Genomics, Inc., San Diego, CA, USA

##### **Correspondence:** H. Cao – BioNano Genomics, Inc., San Diego, CA, USA

**Objectives**

Genome structural variations (SV) have been well established to be associated with diseases and traits; however, SV analysis of human genomes has been severely limited to date by technical shortcomings. Traditionally, SVs have been detected by microarray (limited to imbalanced copy number variation (CNV) with a short dynamic range, low resolution, and relative readouts), next-generation sequencing (NGS) (primarily CNV, some balanced events but too short to span most repeats) and karyotyping and fluorescence *in situ* hybridization (FISH) (both are very low resolution).

**Methods**

Using a single-molecule genome analysis system, BioNano Genomics Irys® System, utilizing next-generation mapping (NGM) technology, it is now possible to comprehensively analyze whole genomes for SVs > 2 kilobase pairs (kbp), including balanced events in a cost-effective and high-throughput manner. This technology allows for the comparison of family pedigrees and populations, which is needed to potentially uncover genomic structural causes of Mendelian and complex diseases.

**Results**

We demonstrate the robustness of NGM for genome-wide discovery of structural variation in the CEPH trio set from the 1000 Genomes Project where the individuals were sequenced and analyzed in-depth. We generated *de novo* assemblies that covered at least 96% of the hg38 reference assembly. Compared to tens of large SV events detected by NGS, we uncovered hundreds of insertions, deletions, and inversions greater than 5 kbp, a large portion of which was novel, and some are located in the regions likely leading to disruption of gene function or regulation. Based on the pedigree structure, we estimated that the Mendelian concordance rate was 96%. We have also begun analysis of a trio of Ashkenazi Jewish descent from the NIST GIAB project, where we have found hundreds of inversions, insertions, and deletions, including large deletions in the UGT2B17 gene (involved in graft versus host disease, osteopathic health, and testosterone and estradiol levels) in the mother and son.

**Conclusion**

We show that NGM is a robust and effective method for structural variation detection in the human genome. Systematic whole genome structural variation within disease population cohorts is needed, in additional to the conventional SNP analysis, to study the effects of a full spectrum of genomic variations in human disease and complex traits.

**Disclosure of interest**

None declared.

## O49 Archaic introgression in indigenous populations of Malaysia revealed by whole genome sequencing

### Y. Chee-Wei^1^, L. Dongsheng^2^, W. Lai-Ping^3^, D. Lian^2^, R. O. Twee Hee^3^, Y. Yunus^4^, F. Aghakhanian^5^, S. S. Mokhtar^4^, C. V. Lok-Yung^1^, J. Bhak^6^, M. Phipps^7^, X. Shuhua^8^, T. Yik-Ying^3^, V. Kumar^1^, H. Boon-Peng^9^

#### ^1^Biotechnology Research Institute, Universiti Malaysia Sabah, Kota Kinabalu, Malaysia; ^2^Shanghai Institutes for Biological Sciences, Chinese Academy of Sciences Shanghai, Shanghai, China; ^3^Saw Swee Hock School of Public Health, National University of Singapore, Singapore, Singapore; ^4^Institute of Medical Molecular Biotechnology, Universiti Teknologi MARA, Sungai Buloh; ^5^Jeffrey Cheah School of Medicine and Health Sciences, Monash University Sunway Campus, Petaling Jaya, Malaysia; ^6^ Personal Genomics Institute, Genome Research Foundation, Suwon, Republic Of Korea; ^7^Jeffrey Cheah School of Medicine and Health Sciences, Monash University Sunway Campu, Petaling Jaya, Malaysia; ^8^Shanghai Institutes for Biological Sciences, Chinese Academy of Sciences Shanghai, Shanghai, China; ^9^UCSI University, Kuala Lumpur, Kuala Lumpur, Malaysia

##### **Correspondence:** H. Boon-Peng – UCSI University, Kuala Lumpur, Kuala Lumpur, Malaysia

**Objectives**

The investigations of the indigenous populations from Malaysia and their migration history have been scarce. In this study, we performed the whole genome sequence for Malaysian indigenous (MI) genomes from Peninsular Malaysia (PM) and Northern Borneo (NB). The genomic structures of these samples were assessed, followed by identification of the archaic genomes introgression between these samples with Denisovan and Neandertal.

**Methods**

Seventeen genomes from indigenous populations from PM and NB were sequenced using Illumina Hi-Seq, thus unveiled the full spectrum of genetic architecture of the MI. Population genetic structure of these samples were assessed with PCA and ADMIXTURE. Inference of coalescent time and effective population size were performed using PSMC, and gene flow was estimated using TreeMix. Archaic genomes introgression was estimated using the D-stat and f-test. S* analysis was applied to identify the introgressed genome segments.

**Results**

The divergence between Negrito and Austronesian occurred ~20K years ago, and were gradually replaced by the Austronesian expansion. Events of multiple gene flow into PM and NB was observed, in line with previous investigations. However no evidence of significantly higher gene flow from archaic genomes to MIs was observed, and that the archaic DNA segments found in MIs were different from those carried by the East Asia and Europeans.

**Conclusion**

Our analyses further strengthens the findings of the population structure of the indigenous people revealed by various earlier studies using SNP array, yet suggests that the history of these populations are far more complex than expected. The archaic genome introgression provided evidence of no significantly higher archaic genome component in our samples. This preliminary study complements the gaps of various speculations about archaic genomes introgression in the regions of SEA and Oceanic.

**Disclosure of interest**

None declared.

## O50 Breast and ovarian cancer prevention: is it time for population-based mutation screening of high risk genes?

### I. Campbell^1^, M.-A. Young^2^, P. James^2^, Lifepool

#### ^1^Research Division, Peter Maccallum Cancer Centre, East Melbourne, Australia; ^2^Familial Cancer Centre, Peter Maccallum Cancer Centre, East Melbourne, Australia

##### **Correspondence:** I. Campbell – Research Division, Peter Maccallum Cancer Centre, East Melbourne, Australia

**Objectives**

Germline mutations in *BRCA1* and *BRCA2* confer high lifetime risk of breast and ovarian cancer but importantly these risks are not irreversible. Identification of asymptomatic carriers could significantly reduce the incidence of these diseases. As a first step toward population based *BRCA* gene screening, we are sequencing the entire coding region of 20 known and proposed HBOC genes in 4,000 cancer-free Australian women.

**Methods**

Cancer-free women were selected from the *LifePool* study (www.lifepool.org) which is a cohort of women attending the Australian population, based mammographic screening program. All exons of the target genes were enriched using the Haloplex system (Agilent) and sequenced on a HiSeq2500 instrument (Illumina). The data were filtered for known pathogenic or novel loss of function mutations.

**Results**

To date, data from 1,997 women has identified 17 with actionable mutations in *BRCA1* (4 mutations), *BRCA2* (9 mutations) or *PALB2* (4 mutations). All 17 women subsequently accepted an invitation to attend a Familial Cancer Centre and then proceeded to formal clinical genetic testing. In addition 4 women had pathogenic mutations in *BRIP1*.

**Conclusion**

Our unique pilot data directly demonstrates a population carrier frequency of ~1% for pathogenic mutations in these recognized high risk breast and/or ovarian cancer genes and that such testing is well accepted by the screened population.

**Disclosure of interest**

None declared.

## O53 Comprehensive coverage from low DNA input using novel NGS library preparation methods for WGS and WGBS

### C. Schumacher, S. Sandhu, T. Harkins, V. Makarov

#### Swift Biosciences Inc, Ann Arbor, MI, USA

##### **Correspondence:** C. Schumacher – Swift Biosciences Inc, Ann Arbor, MI, USA

**Objectives**

In order to conduct comprehensive analysis of whole genome sequencing (WGS) or whole genome bisulfite sequencing (WGBS), unbiased, even coverage of the genome is required. To maximize time and cost efficiency, it is imperative to attain coverage from the lowest possible sequence read depth. Highly efficient conversion of DNA fragments into library molecules is especially imperative when DNA input quantity or quality is limited. To address these concerns, we have developed two novel library preparations which enable highly efficient DNA library preparation from low input while maintaining even genomic coverage.

**Methods**

The WGS method uniquely repairs damage on both the 3′ and 5′ termini to enhance ligation efficiency to DNA fragments. Combined with sequential ligation steps, this single tube method supports PCR-free sequencing from inputs as low as 10 ng circulating, cell-free DNA (cfDNA) or 50 ng physically sheared DNA. For WGBS, our library preparation is performed on denatured, bisulfite-converted fragments. This improves library recovery significantly compared to traditional library prep methods that ligate methylated adapters to double-stranded DNA prior to bisulfite conversion. Our adapter attachment to single-stranded DNA supports inputs from 100 pg to 10 ng Input quantities down to 10 pg can be used with PCR amplification.

**Results**

Using the WGS method, library conversion efficiency was ~50% for physically sheared DNA and up to 90% for cfDNA. Human WGS using this method demonstrates high complexity with exceptional coverage of GC-rich promoter regions. At inputs as low as 1 ng human DNA, at 16X coverage, the genome was fully represented with consistent, uniform coverage. Libraries made with the WGBS method required less PCR amplification than other available kits and this improvement was further seen in the sequencing data, particularly at 1 ng input. Human WGBS demonstrated comprehensive coverage of CpG islands when 10 ng input was used at low depth of sequencing. This library preparation method enables single base resolution of methylation status throughout the genome, even from limiting DNA input quantities.

**Conclusion**

We have demonstrated the utility of increasing the efficiency of library preparation as a means of improving sequencing results obtained through both WGS and WGBS. This innovative technology enables sequencing of sample types that have been previously unusable due to input or quality limitations.

**Disclosure of interest**

C. Schumacher Shareholder of: Swift Biosciences Inc, Employee of: Swift Biosciences Inc, S. Sandhu Shareholder of: Swift Biosciences Inc, Employee of: Swift Biosciences Inc, T. Harkins Shareholder of: Swift Biosciences Inc, Employee of: Swift Biosciences Inc, V. Makarov Shareholder of: Swift Biosciences Inc, Employee of: Swift Biosciences Inc.

## O54 Methods for large scale construction of robust PCR-free libraries for sequencing on Illumina HiSeqX platform

### H. Doddapaneni, R. Glenn, Z. Momin, B. Dilrukshi, H. Chao, Q. Meng, B. Gudenkauf, R. Kshitij, J. Jayaseelan, C. Nessner, S. Lee, K. Blankenberg, L. Lewis, J. Hu, Y. Han, H. Dinh, S. Jireh, K. Walker, E. Boerwinkle, D. Muzny, R. Gibbs

#### Human Genome Sequencing Center, Baylor College of Medicine, Houston, TX, USA

##### **Correspondence:** H. Doddapaneni – Human Genome Sequencing Center, Baylor College of Medicine, Houston, TX, USA

**Objectives**

With drastic drop in the cost of whole genome sequencing (WGS) on Illumina’s HiSeqX systems, it’s now economically feasible to plan large scale sequencing projects in research as well as clinical settings. However, successful execution of such projects will require design of robust sample preparation (library) workflows that can work with a spectrum of DNA quality and quantities. This is especially true for the highly desirable PCR-Free libraries that are known to provide improved gene and genome representation compared to PCR amplified libraries. Here we discuss the use of multiple PCR-Free protocols for use on HiSeqX.

**Methods**

Previously we automated Illumina TruSeq PCR-Free protocol, which is recommended to use with 1 ug of good quality DNA to prepared size selected libraries. However, to broaden the scope of samples that can be used for preparing PCR-Free libraries, we evaluated two additional library construction methods 1. Swift Biosciences 2S library kit and 2. Kapa Biosystems, hyper prep kit. PCR-Free libraries were prepared using 200 ng - 1 ug DNA of HGSC internal human control sample (HS1011) with these kits and sequenced on HiSeqX to generate 34-38X genome coverage data.

**Results**

Exome representation as measured by complete coverage of Online Mendelian Inheritance in Man (OMIM) genes at 20X read depth was lowest for the TruSeq PCR-Free libraries (2687 genes) when compared to the 2S libraries (2750 – 3020 genes) and the Kapa hyper libraries (2800 genes). GC representation was better in PCR-free libraries when compared to the TruSeq Nano libraries. Kapa Hyper protocol was optimized on Beckman Coulter’s Biomek FXP liquid handler using 500 ng DNA and can prepare 96 libraries in ~ 6 hours. This protocol also works well with DNA of different integrities. Enhancements have also been made for precise quantification of PCR-Free libraries by qPCR and to eliminate unused adapter molecules in libraries that can impact sequencing.

**Conclusion**

Availability of such robust and automated protocols has positioned us to efficiently work with large sample sets to fully exploit the use of HiSeqX platforms for population level genomic studies and to drive its use in clinical setting.

**Disclosure of interest**

None declared.

## O55 Rapid capture methods for clinical sequencing

### J. Hu^1^, K. Walker^1^, C. Buhay^1^, X. Liu^1^, Q. Wang^1^, R. Sanghvi^1^, H. Doddapaneni^1^, Y. Ding^1,2^, N. Veeraraghavan^1^, Y. Yang^2^, E. Boerwinkle^1,3^, A. L. Beaudet^2^, C. M. Eng^2^, D. M. Muzny^1^, R. A. Gibbs^1^

#### ^1^Human Genome Sequencing Center, Baylor College of Medicine, Houston, TX, USA; ^2^Department of Molecular and Human Genetics, Baylor College of Medicine, Houston, TX, USA; ^3^Human Genetics Center, University of Texas Health Science Center at Houston, Houston, TX, USA

##### **Correspondence:** J. Hu – Human Genome Sequencing Center, Baylor College of Medicine, Houston, TX, USA

**Objectives**

Advancement of next generation sequencing in clinical settings has required methods for rapid, robust delivery of high-quality sequencing data. Effective and timely diagnoses or prediction of risk of genetic diseases are important for medical intervention.

**Methods**

We have developed a ‘*lightning capture*’ process to deliver variant calls in 5–7 days after sample intake. This process includes: quick enrichment library preparation (5–6 hours), capture enrichment (about 8 hours), rapid sequencing (Illumina HiSeq2500) and data analysis via the HGSC-developed Mercury pipeline. The lightning capture protocol has been deployed in BMGL clinic lab for whole exome sequencing (WES) and recent carrier screening with a novel 500kb carrier mutation gene capture panel. Our WES design contains 3643 clinically relevant genes primarily from GeneTests and OMIM, and the carrier panel includes 168 complete genes that contain at least 850 known common genetic variants of clinical relevance. We also employ the genotyping with SNPTrace panel by Fluidigm in order to ensure reliable sample identification, and to test for sample cross-contamination.

**Results**

This method has been validated with ~500 WES and more than 5000 carrier samples. WES samples are processed in single capture or 3-plex co-capture, while carrier samples are in a cost-effective 47plex co-capture format for hybridization followed by sequencing of 94 samples (2 capture pools) per HiSeq 2500 lane. High enrichment efficiency was observed (72-80% reads on target and buffer) and superior coverage metrics across the design with 11 Gb sequencing yield for WES and ~400 Mb for Carrier samples. A detailed analysis of the carrier design performance using 140 de-identified samples found that known carrier mutations were correctly identified with high confidence (98.5%), including large/complex indel mutations.

**Conclusion**

Sample turnaround time for tests is often considered one of the most significant measures of performance for a clinical lab. The lightning capture process we developed enables data delivery in 5–7 days without sacrifice of data quality. This novel method may impact applications in prenatal, neonatal intensive care and other critical settings for clinical and research samples.

**Disclosure of interest**

None declared.

## O56 A diploid personal human genome model for better genomes from diverse sequence data

### K. C. C. Worley^1^, Y. Liu^1^, D. S. T. Hughes^1^, S. C. Murali^1^, R. A. Harris^1^, A. C. English^1^, X. Qin^1^, O. A. Hampton^1^, P. Larsen^2^, C. Beck^3^, Y. Han^1^, M. Wang^1^, H. Doddapaneni^1^, C. L. Kovar^1^, W. J. Salerno^1^, A. Yoder^2^, S. Richards^1^, J. Rogers^1^, J. R. Lupski^3^, D. M. Muzny^1^, R. A. Gibbs^1^

#### ^1^Human Genome Sequencing Center, Molecular and Human Genetics, Baylor College of Medicine, Houston, TX, USA; ^2^Department of Biology, Duke University, Durham, NC, USA; ^3^Molecular and Human Genetics, Baylor College of Medicine, Houston, TX, USA

##### **Correspondence:** K. C. C. Worley – Human Genome Sequencing Center, Molecular and Human Genetics, Baylor College of Medicine, Houston, TX, USA

**Objectives**

Generate a high quality reference genome from a single individual to avoid limitations of the haploid mosaic human reference and explore the contribution of different data types to understanding the comprehensive clinical genome.

**Methods**

Illumina data from a variety of libraries (180 bp, 300 bp and 500 bp paired end data; 3kb, 6.5 kb and 8 kb mate-pair data), as well as Illumina Hi-C data, 20x PacBio RS long read data and BioNano optical mapping data were produced and assembled *de novo*. Structural variants that are difficult to characterize with exome sequencing or short sequence reads from small fragments were identified using two methods. We identified putative novel insertions in reads that did not map to the GRCh38 reference with calls supported by 6.5 kb mate-pair data, and confirmed in the *de novo* WGS assembly contigs and 3 kb mate-pair data. We identified tandem duplications with combined signatures of inverted 300 bp to 500 bp read pairs identifying the boundaries and larger read pairs confirming the size of the duplicated region.

**Results**

We report here the assembly of data from a single individual (HS1011). The data are available at NCBI under BioProject 203659. The released assembly is highly contiguous with a 394 kb Contig N50 and 148 Mb Scaffold N50 with full chromosome scaffolds. Data from the parents of HS1011 and long-read data allow us to phase variants within this genome. Putative novel insertions (78) and tandem duplications (70) were identified.

**Conclusion**

The assembly and underlying data reported here allow us to optimize methods to combine these data types and explore the utility of the different data types to identify structural variation and define which heterozygous variants are located on the same haplotype for haplotype-aware downstream analyses.

**Disclosure of interest**

None declared.

## O57 Development of PacBio long range capture for detection of pathogenic structural variants

### Q. Meng, M. Bainbridge, M. Wang, H. Doddapaneni, Y. Han, D. Muzny, R. Gibbs

#### HGSC, Baylor College of Medicine, Houston, TX, USA

##### **Correspondence:** Q. Meng – HGSC, Baylor College of Medicine, Houston, TX, USA

**Objectives**

Discoveries of chromosomal alterations ranging from single nucleotide variants (SNVs) to large structural variants (SVs), via next-generation sequencing (NGS) techniques, have drastically impacted the delineation of genomic architecture and disease mechanisms. However, in contrast to being able to identify SNVs efficiently and accurately, current short-read NGS platforms have been inefficient for discovery of complex chromosomal rearrangements. To detect chromosomal pathogenic SVs, we have developed a novel method, Pacific Biosystems large insert targeted capture-sequencing (PB-LITS). We further tested PB-LITS protocol for detection of clinical relevant complex gene structural mutations with a set of 6 breast cancer samples.

**Methods**

Genomic DNA was fragmented to 6kb and ligated with Illumina adaptors, followed by size-selection at 4.5kb-9kb range. Long range PCR was performed on size-selected inserts. Pre-Capture PCR products were hybridized with a set of NimbleGen SeqCap probes targeting 94 genes that represent a spectrum of diseases with low, and thus unsatisfactory, sequencing access by current short-read NGS methods. Captured products were processed by standard PacBio large insert library preparation procedure. The final product, a 6kb insert capture library with single molecule, real-time (SMRT) bell adaptors, was sequenced on PacBio RSII platform.

**Results**

Initial PB-LITS protocol has shown its value of discerning complex structural rearrangements with 1.5ug input DNA^1^. Further development of PB-LITS enabled successful construction of 6kb-insert capture library with minimal 150ng input, by optimizing adaptor ligation and long range PCR reaction. Sequencing with 2~3 SMRT cells of HapMap DNA libraries constructed by current protocol yielded 20x coverage of 93% of 14mb target size with an average insert size of 3.7kb-7.1kb. Sequencing and further analysis of the libraries constructed from 6 cases with strong family histories of breast cancer who previously tested negative for mutations in BRCA1 and BRCA2 are undergoing.

**Conclusion**

Optimized PB-LITS protocol provided a reliable research tool for studying complex chromosomal SVs. Development of library construction procedure of PB-LITS has laid solid foundation to employ the method in clinical settings where identification of pathogenic complex SVs with limited sample amount is challenging.

**References**

1. Wang M, et al. PacBio-LITS: a large-insert targeted sequencing method for characterization of human disease-associated chromosomal structural variations. BMC Genomics. 2015; 16:214.

**Disclosure of interest**

None declared.

## O58 Rhesus macaques exhibit more non-synonymous variation but greater impact of purifying selection than humans

### R. A. Harris^1,2^, M. Raveenedran^1,2^, C. Xue^1,^, M. Dahdouli^1,2^, L. Cox^3^, G. Fan^4^, B. Ferguson^5^, J. Hovarth^6^, Z. Johnson^7^, S. Kanthaswamy^8^, M. Kubisch^9^, M. Platt^10^, D. Smith^11^, E. Vallender^12^, R. Wiseman^13^, X. Liu^14^, J. Below^15^, D. Muzny^1,2^, R. Gibbs^1,2^, F. Yu^1,2^, J. Rogers^1,2^

#### ^1^Molecular and Human Genetics, Baylor College of Medicine, Houston, TX, USA; ^2^Human Genome Sequencing Center, Baylor College of Medicine, Houston, TX, USA; ^3^Genetics, Southwest National Primate Research Center, San Antonio, TX, USA; ^4^Human Genetics, Univeristy of California Los Angeles, Los Angeles, CA, USA; ^5^Division of Neuroscience, Oregon National Primate Research Center, Beaverton, OR, USA; ^6^Genomics & Microbiology Research Laboratory, NC Museum of Natural Sciences, Raleigh, NC, USA; ^7^Yerkes Nonhuman Primate Genomics Core, Yerkes National Primate Research Center, Atlanta, GA, USA; ^8^Environmental Toxicology, California National Primate Research Center, Davis, CA, USA; ^9^Physiology, Tulane National Primate Research Center, New Orleans, LO, USA; ^10^Neuroscience, University of Pennsylvania, Philadelphia, PA, USA; ^11^Anthropology, University of California Davis, Davis, CA, USA; ^12^Psychiatry and Human Behavior, University of Mississippi Medical Center, Jackson, MS, USA; ^13^Genetics, Wisconsin National Primate Research Center, Madison, WI, USA; ^14^Epidemiology, Human Genetics & Environmental Sciences; ^15^Epidemiology and Disease Control Human Genetics Center, University of Texas Health Science Center, Houston, TX, USA

##### **Correspondence:** R. A. Harris – Molecular and Human Genetics, Baylor College of Medicine, Houston, TX, USA

**Objectives**

We generated whole genome sequence data for 133 rhesus macaques (*Macaca mulatta*), the primary nonhuman primate in biomedical research.

**Methods**

Using the intersection of GATK and SNPTools SNV calls, we identified 43 million high-quality SNVs, including >126,000 missense and >148,000 synonymous coding variants.

**Results**

Comparisons with equivalent whole genome data from the Human 1000 Genomes project shows that macaques have 2.5-fold higher levels of overall variation and 20% higher levels of nonsynonymous variation per individual. Comparing the ratio of nonsynonymous to synonymous variants between species shows a lower ratio of NS:Syn in macaques, indicating more effective purifying selection, which can be explained by higher effective population size. Looking more specifically at 740 genes from the SFARI autism genetic association database, the ratio of NS:Syn variants among the macaques is lower in the SFARI gene set than in the complete macaque gene set.

**Conclusion**

These data suggest that large effective size in macaques leads to higher levels of both total and nonsynonymous variation than humans, and that purifying selection in macaques is more efficient in restricting mildly deleterious mutations.

**Disclosure of interest**

None declared.

## O59 Assessing RNA structure disruption induced by single-nucleotide variation

### Z. Ouyang^1^, J. Lin^1^, Y. Zhang^2^

#### ^1^The Jackson Laboratory for Genomic Medicine, Farmington, CT, USA; ^2^Department of Statistics, University of Connecticut, Storrs, CT, USA

##### **Correspondence:** Z. Ouyang – The Jackson Laboratory for Genomic Medicine, Farmington, CT, USA

**Objectives**

It has been challenging to interpret noncoding variants in complex traits and human diseases. RiboSNitches, single-nucleotide variants (SNVs) that alter the structures of RNAs, have recently found in many human diseases. RNA structure change mediated by riboSNiches has emerged as a plausible mechanism of pathogenic consequences of mutations. Thus, it is desirable to automatically predict riboSNitches from millions of SNVs of the human genome. However, current computational methods based on *in silico* RNA-folding algorithms suffer from limited accuracy. We seek to develop a new method for improved riboSNitch detection.

**Methods**

We introduce a new measurement to quantify the structural difference between the wild-type and mutant RNAs in which the two sequences are differed by an SNV. The new measurement is based on assessing the consistency of RNA structure change of individual bases. Using this new measurement, our method automatically selects a region that maximizes the effect of RNA structure disruption induced by an SNV.

**Results**

We applied our method to analyze a genome-scale dataset of riboSNitches and non-riboSNitches determined from the parallel analysis of RNA structure experiments on a family trio of human lymphoblastoid cell lines. The dataset contains rigorously validated subsets of 11 “probed”, 63 “validated”, and 223 “symmetric” riboSNitches. We found that our method consistently outperforms other existing methods on these rigorously validated subsets of riboSNitches.

**Conclusion**

Our new method improves the accuracy of computational prediction of riboSNitches. It facilitates the prioritization of noncoding variants for interpreting personal genomes. It also holds the promise to identify disease-causing variants potentially through RNA structure disruption.

**Disclosure of interest**

None declared.

## P1 A meta-analysis of genome-wide association studies of mitochondrial dna copy number

### A. Moore^1^, Z. Wang^2^, J. Hofmann^3^, M. Purdue^1^, R. Stolzenberg-Solomon^1^, S. Weinstein^1^, D. Albanes^1^, C.-S. Liu^4^, W.-L. Cheng^4^, T.-T. Lin^4^, Q. Lan^1^, N. Rothman^1^, S. Berndt^1^

#### ^1^National Cancer Institute, Rockville, USA; ^2^St. Jude Children’s Research Hospital, Memphis,USA; ^3^National Cancer Institute, NIH, DHHS, Rockville, USA, ^4^Changhua Christian Hospital, Changhua, Taiwan, Province of China

##### **Correspondence:** A. Moore – National Cancer Institute, Rockville, USA

**Objectives:** Variation in mitochondrial DNA (mtDNA) copy number (CN) has been shown to be related to the risk of several cancers in prospective studies. The inter-individual variability of mtDNA CN is thought to be partially heritable; however, no genome-wide association study (GWAS) of the nuclear genome has yet been performed.

**Methods:** We conducted a meta-analysis of GWAS of peripheral blood mtDNA CN using data from participants of European ancestry from nested case–control studies of prostate cancer and non-Hodgkin lymphoma in the Prostate, Lung, Colorectal, and Ovarian (PLCO) Screening Trial (n=1664). MtDNA CN was natural log-transformed and linear regression was used to evaluate the association, assuming an additive genetic model and adjusting for age at mtDNA blood draw, ancestry, and sex. The three GWAS were combined in a fixed-effects meta-analysis.

**Results:** A quantile-quantile plot of the association results revealed some enrichment for SNPs with small p-values, but no evidence of genomic inflation (λ=1.007). Six loci, defined as +/− 1 Megabase, reached genome-wide significance (p < 5X10^−8^), but all appeared to be singletons, indicating that they are likely to be false positives. Ten Single Nucleotide Polymorphisms (SNPs) in an intronic region of the long-range sonic Hedgehog signaling gene, *DISP1,* were found suggestively associated (p < 5X10^−5^)with mtDNA CN, with consistency in the direction of associations among all three GWAS.

**Conclusion:** Preliminary findings from this meta-analysis suggest that there may be common genetic variants of the nuclear genome associated with mtDNA CN. We are currently augmenting our meta-analysis by including additional GWAS of nested case–control studies in PLCO and the Alpha-Tocopherol, Beta-Carotene Cancer Prevention Study. We expect that the added statistical power will yield novel loci for mtDNA CN and provide new insight into the regulation of mtDNA CN.

**Competing interests**

None declared.

## P2 Missense polymorphic genetic combinations underlying down syndrome susceptibility

### E. S. Chen

#### Biochemistry, National University of Singapore, Singapore, Singapore

**Objectives:** Single nucleotide polymorphisms (SNPs) drawn much attention as prospective biomolecular markers for human health management and disease therapies. Moreover SNPs have been surmised to constitute unique genetic makeup characteristic to each human individual, which can be utilized to cater therapeutic approaches to personalized medical care. Down syndrome (DS) or trisomy 21 is the most frequently occurring birth-related defects affecting live-born children. Molecular mechanisms that regulate and/or result in the formation of trisomy 21 in DS mothers, remain hitherto unknown. We posit a genetic basis for disposition of DS occurrence. We therefore performed bioinformatics studies of published nonsynonymous SNPs in conjunction with structural information of the proteins encoded by DS risk genes in the attempt to identify novel governing principles of DS risk.

**Methods:** We surveyed all SNPs in the published literature focusing on missense mutations, and superimpose on bioinformatic reconstruction of secondary structural motifs of proteins encoded by DS genes

**Results:** In our survey, we observed that even the most penetrant SNP implicated in DS is not completely associated with the disease. On the other hand, a combination of co-occurrence of SNPs is important, suggesting a synthetic cooperation between missense mutations to underlie occurrence of DS phenotype. Superimposing documented SNPs from several public databases showed a preferential localization of these SNPs with specific structural motifs within the proteins. Interesting, we noticed several closely situated SNPs that have not been reported to be associated with DS risk within regulators of the one carbon folate metabolism that included reduced folate carrier 1 (RFC1) and methionine synthase. These may represent novel SNPs that can be assessed experimentally in a targeted manner in future population studies.

**Conclusion:** Taken together, our analyses showed that SNPs that result in change of protein sequences act synergistically to impact DS phenotype and suggest that SNP combinations to be a more reliable criteria than single SNPs for ascertaining DS risk, at least in the case of missense SNPs. Our study also identified probable secondary structural motifs implicated in DS risk-associating factors. These results will form the basis for future experiments that may hold potential for translation into personalized diagnosis or therapeutic management of DS.

**Competing interests**

None declared.

## P8 Differentiating inflammatory bowel diseases by using genomic data: dimension of the problem and network organization

### N. Mili^1^, R. Molinari^1^, Y. Ma^2^, S. Guerrier^3^

#### ^1^Research Center for Statistics, University of Geneva, Switzerland, Geneva, Switzerland; ^2^Department of Statistics, University of South Carolina, Columbia, USA: ^3^Department of Statistics , University of Illinois at Urbana Champaign, Champaign, USA

##### **Correspondence:** N. Mili – Research Center for Statistics, University of Geneva, Switzerland, Geneva, Switzerland

**Objectives:** To determine the minimum number of genes (dimension of the problem) and their network organization in the distinction between Crohn’s Disease (CD), Ulcerative Colitis (UC) and normal patients (N).

**Methods:** We relied on the results published by Burczynski *et al.* where transcriptional profiles in peripheral blood mononuclear cells (PBMC) from 42 healthy individuals, 59 CD patients, and 26 UC patients were assessed. We applied a newly proposed gene selection method, based on statistical and machine-learning principles, which finally delivered a set of models which best predicted the disease class. These models were inserted in a network where the biomarkers were placed in specific positions according to their relevance in discriminating between the diseases.

**Results:** We found that a set of models, each containing only two RNA’s from the PBMC, were sufficient to discriminate CD from UC patients and normal individuals. These RNA’s were organized in networks where the gene in first position could well classify when placed in a model with any of those in the corresponding second position. A summary of these networks is as follows.

**Conclusion:** Our statistical method is a new powerful tool that gives (1) the dimension of the statistical model, (2) the network organization of the selected genomic biomarkers, (3) a set of interchangeable models giving the same information.

Moreover, all the RNA’s in position 1 of the selected networks are known to have a clinical significance in Inflammatory Bowel Diseases. Amyloidosis is a well-known complication of CD and UC (Amyloid beta A4 precursor protein). Chemotaxis and neutrophil activation are fundamental pathways in the pathogenesis of IBD (Chemokine C-X-C motif ligand 5). RAB31 enhances FcγR-mediated phagocytosis through PI3K/Akt signaling in macrophages and plays a role in the maturation of phagosomes. Some clinical evidence links the menstrual cycle to the IBD activity (Progesterone receptor membrane component 1).

**References**

Michael E. Burczynski et al. *Molecular Classification of Crohn’s Disease and Ulcerative Colitis Patients Using Transcriptional Profiles in Peripheral Blood Mononuclear Cells*. J Mol Diagn. 2006 Feb; 8(1): 51–61.

Stephane Guerrier et al. *A Paradigmatic Regression Algorithm for Biomarker Selection Problems. Submitted*.

**Competing interests**

None declared.

## P9 Vulnerability of genetic variants to the risk of autism among Saudi children

### N. Elhawary^1,2^, M. Tayeb^2^, N. Bogari^2^, N. Qotb^3^

#### ^1^Department of Molecular Genetics, Medical Genetics Center, Ain Shams University, Cairo, Egypt; ^2^Department of Medical Genetics, Umm Al-Qura University, Saudi Arabia; ^3^Department of Psychology, Umm Al-Qura University, Faculty of Education, Mecca, Saudi Arabia

##### **Correspondence:** N. Elhawary – Department of Molecular Genetics, Medical Genetics Center, Ain Shams University, Cairo, Egypt

**Objectives:** Single nucleotide polymorphisms (SNPs) have been reported in different autistic populations. Here we present the first association study investigating SNPs of some genes; *serotonin receptor (HTR2A* IVS2A>G rs7997012; *HTR2C* 68G>C rs6318*), serotonin transporter* (*SLC6A4* rs3813034), *ankyrin repeat and kinase domain containing 1*(*ANKK1* rs1800497)*, methylenetetrahydrofolate reductase* (*MTHFR* rs1891394)*,* and *BDNF* rs6265 in Saudi autistic children. Epidemiologic, clinical and psychometric aspects were used to examine the possible risk factors of autism.

**Methods:** We used TaqMan SNP genotyping to examine 68 Saudi children (48 Males and 20 females) diagnosed with autism according to DSM-IV criteria & ICD-10 criteria, including deficits in reciprocal social interaction, impaired verbal, and non-verbal communication as well as restricted, repetitive, and stereotyped patterns of behaviors. Healthy controls (n= 78) with no history of mental illnesses, behavioral disorders or substance abuse were used. The severity of behavioral symptoms in cases was assessed at admission using the Childhood Autism Rating Scale (CARS). Hardy-Weinberg equilibria of the genetic variants were assessed using online software (http://www.oege.org/software/hwe-mr-calc.shtml). C*hi*-square tests were used to compare sociodemographic and clinical characteristics. Odds ratios and confidence intervals were calculated.

**Results:** Overall, our data provide strong evidence of associations between these two SNPs and risk of autism in this population. Compared to healthy subjects, children with autism showed significant overexpression of the mutant alleles in the SNPs rs7997012, rs6318, rs3813034, rs1800497, and rs1891394, but not in the rs6265 SNP. Data on linkages or associations between these genetic loci and the disease vary among different ethnicities.

**Conclusion:** Our findings presented associations between autism and some genetic variants under investigation, and showed that the potential influence of just one copy of the mutant alleles in the Saudi patients. Replicating our findings in other ethnic population may support our data before making any firm generalizations. Ongoing analyses of genetic variants associated with autism are being extended using next-generation sequencing and some fruitful data are going to be published.

**Competing interests**

None declared.

## P10 Chromatin profiles from ex vivo purified dopaminergic neurons establish a promising model to support studies of neurological function and dysfunction

### S. A. McClymont, P. W. Hook, L. A. Goff, A. McCallion

#### Institute of Genetic Medicine, Johns Hopkins School of Medicine, Baltimore, USA

##### **Correspondence:** S. A. McClymont – Institute of Genetic Medicine, Johns Hopkins School of Medicine, Baltimore, USA

**Objectives:** The majority of variants associated with common disease reside in non-coding sequence. Efforts to understand complex disease risk thus focus heavily on regulatory variation. How to seek biologically relevant sequences in which variation may reside and how to make *in vivo* predictions of the impact and function of regulatory variation remains a major challenge. We recently demonstrated how GWAS results can be informed by enhancer analyses in homogenous, phenotype-appropriate cell populations. Further, such enhanced datasets make feasible the prediction of functional variants. Presently, we are leveraging our past experiences to generate enhancer catalogues using homogenous populations of dopaminergic (DA) neurons better to understand the role of regulatory variation in Parkinson Disease (PD) and related disorders.

**Methods:** We assayed open chromatin regions using ATAC-seq, producing multiple enhancer catalogues. Each catalogue is generated from 50,000 FAC-sorted DA neurons from either the forebrain (n=3 libraries) or midbrain (n=3 libraries) of E15.5 transgenic mice. Each ATAC-seq library is of high quality, yields over 30 million reads sequenced, with over 28 million reads mapping, and following filtering of reads, MACS2 calls ~50,000 peaks, indicating intervals of open chromatin.

**Results:** Our preliminary analyses indicate that peaks from all libraries show evidence of functional sequence constraint (PhastCons scores>0.3) and significant enrichment for processes and functions appropriate to neuronal function/dysfunction by GO/GREAT. The high quality of these open chromatin signatures also facilitate development of a computational classifier (regulatory vocabulary) of DA neurons. Consequently, we have begun to gain further insight into the transcription factors active in DA neurons and the nature of variation that might influence the function of identified DA enhancers.

**Conclusion:** We are currently validating the DA neuron enhancer catalogues *in vivo* and are evaluating the shared and unique content of the catalogues and their pertinence to disease. Additionally, we have generated single cell and bulk RNA-seq from these isolated populations to corroborate and inform our chromatin-based findings. With this data in hand, we are able to assay the impact of PD and related movement disorders’ GWAS-implicated variation on DA neuron function and disease pathogenesis.

**Competing interests**

None declared.

## P11 Utilization of a sensitized chemical mutagenesis screen to identify genetic modifiers of retinal dysplasia in homozygous Nr2e3^rd7^ mice

### Y. Kong^1,2^, J. R. Charette^1^, W. L. Hicks^1^, J. K. Naggert^1^, L. Zhao^1^, P. M. Nishina^1,2^

#### ^1^The Jackson Laboratory, Bar Harbor, USA ^2^Graduate School of Biomedical Science and Engineering, University of Maine, Orono, USA

##### **Correspondence:** Y. Kong – The Jackson Laboratory, Bar Harbor, USA

**Objectives:** To identify and characterize genetic modifiers capable of altering the retinal dysplasia observed in *Nr2e3*^*rd7*^ mutants, a model for human Enhanced S-Cone Syndrome (ESCS).

**Methods:***Nr2e3*^*rd7*^ mice were chemically mutagenized, and mated to generate a G3 population. The G3 mice were screened by indirect ophthalmoscope to establish lines bearing genetic modifiers that altered the pan retinal fundus spotting phenotype, characteristic of homozygous *Nr2e3*^*rd7*^ mice. Quantitative trait locus analysis combined with high-throughput sequencing of an exome capture libraries was used to identify the molecular basis of modifiers of the retinal dysplasia in *Nr2e3*^*rd7*^ mutants. Apart from fundus imaging, longitudinal histological studies were carried out to characterize the progression of altered retinal phenotypes. Finally, immunoblotting and marker analyses were performed to reveal the defects that underlie the *rd7*-retinopathy and how the defects were affected by the modifier(s).

**Results:** Seven heritable modifier mouse lines with an altered retinal phenotype have been established so far. Among them three potential genetic modifiers have been identified, which directly or indirectly are associated with growth and development of neuroretinal cells. For example, the *Tvrm272* line bears a nonsense mutation of the *Rarb* gene, which leads to a vitreal dysplastic phenotype that is more severe in the presence of the *Nr2e3*^*rd7*^ mutation. While a reduced retinal spotting phenotype and suppression of formation of rosette-like structures associated with the *rd7* retinopathy in the *Tvrm222* line is due to a missense mutation in the *Frmd4b* gene. The modifying effect of the *Frmd4b*^*Tvrm222*^ allele is achieved through its effect on cell-cell junctions, revealed by immunoblotting and marker analysis.

**Conclusion:** As an animal model of ESCS, retinal dysplasia in *Nr2e3*^*rd7*^ mouse can be phenotypically altered by multiple genetic modifiers via different pathways. The modifying effects on *rd7*-associated retinopathies by these particular genetic modifiers, to our knowledge, are the first to be described. They provide novel insights into the pathogenesis of retinal dysplasia as well as degeneration in *Nr2e3*^*rd7*^-associated disease, and may become potential interfering targets for clinical applications against ESCS and related retinopathies.

**Competing interests**

None declared.

## P12 Ion torrent next generation sequencing of recessive polycystic kidney disease in Saudi patients

### B. M. Edrees^1^, M. Athar^1^, F. A. Al-Allaf^1^, M. M. Taher^1^, W. Khan^2^, A. Bouazzaoui^1^, N. A. Harbi^3^, R. Safar^4^, H. Al-Edressi^4^, A. Anazi^5^, N. Altayeb^6^, M. A. Ahmed^7^, K. Alansary^8^, Z. Abduljaleel^1^

#### ^1^Department of Medical Genetics, Faculty of Medicine, Umm Al-Qura University, Makkah, Saudi Arabia; ^2^Department of Basic Sciences, College of Science and Health Professions, King Saud Bin Abdulaziz University for Health Sciences, Riyadh, Saudi Arabia; ^3^Department of Pediatric, King Faisal Specialist Hospital and Research Centre, Jeddah, Saudi Arabia; ^4^Department of Pediatric, Madinah Maternity and Children’s Hospital, Madinah,Saudi Arabia; ^5^Pediatric, King Fahad Medical City, Riyadh, Saudi Arabia; ^6^Molecular Diagnostics Unit, Department of Laboratory and Blood Bank, King Abdullah Medical City, Makkah, Saudi Arabia; ^7^Medical Genetics, King Salman Armed Forces Hospital, Tabuk, Saudi Arabia; ^8^Medical Genetics, King Fahad Medical City, Riyadh, Saudi Arabia

##### **Correspondence:** Z. Abduljaleel – Department of Medical Genetics, Faculty of Medicine, Umm Al-Qura University, Makkah, Saudi Arabia

**Objectives:** Targeted customized sequencing of genes implicated in the Autosomal recessive polycystic kidney disease (ARPKD) phenotype to identify candidate variants using next-generation sequencing by Ion torrent PGM.

**Methods:** Eighteen unrelated ARPKD probands and healthy human adult control samples were recruited for genetic screening of ARPKD at a referral hospital from northern region of Saudi Arabia. Probands had survived the neonatal period and had age range in between 2 months to 13 years.

*Ion-Torrent PGM sequencing:* We have customized primers and a target enrichment kit for targeting of the ARPKD candidate gene (*PKHD1)*, and also included the *PKD1* and *PKD2* genes that may mimic the phenotype of the ARPKD. The NGS protocol involves three steps (1) Library preparation (2) Template preparation, finally sequencing was performed on PGM using Ion PGM 200 sequencing kit.

*Mapping assembly and variants discovery from NGS data:* For each resulted deleterious variants identified by NGS were also confirmed by Capillary sequencing.

**Results:** We identified five potential pathogenic missense variants in *PKHD1* gene in 12 ARPKD Saudi patients. One missense variant was novel and other four had been reported in other ethnic groups but not in Saudis. The rest of the patient’s samples have few variants in *PKD1* and *PKD2* genes that were not in damage but two causative variants observed. One missense homozygous variant c.4870C>T, p.(Arg1624Trp) was common in eight patients in PKHD1 gene derived from a male proband. Our results showed that the deleterious missense variant detected in *PKHD1* gene was pathological significant or damaged were identified by computational predictions Sorting Intolerant From Tolerant (SIFT) and Polymorphism Phenotyping (PolyPhen2). Taken together, this strategy significantly lowers the cost and time for simultaneous targeted genes sequence analysis, and facilitating routine genetic diagnostics of ARPKD.

**Conclusion:** Overall, the NGS TargetSeq exome sequencing may prove to be advantageous in the early diagnosis in patients with ARPKD disease.

**Competing interests**

None declared.

## P13 Digital expression profiling of Purkinje neurons and dendrites in different subcellular compartments

### A. Kratz^1^, P. Beguin^2^, S. Poulain^1^, M. Kaneko^2^, C. Takahiko^2^, A. Matsunaga^2^, S. Kato^1^, A. M. Suzuki^1^, N. Bertin^1^, T. Lassmann^1^, R. Vigot^1^, P. Carninci^1^, C. Plessy^1^, T. Launey^2^

#### ^1^Center for Life Science Technologies, RIKEN Yokohama, Yokohama City, Kanagawa, Japan; ^2^Brain Science Institute (BSI), Launey Research Unit, RIKEN Wako, Wako, Japan

##### **Correspondence:** A. Kratz – Center for Life Science Technologies, RIKEN Yokohama, Yokohama City, Kanagawa, Japan

**Objectives:** Neuronal cells are not homogeneously distributed and subtypes are intermingled with each other as well as with non-neuronal cells such as glia and blood vessel cells. When attempting to digitally profile the expression of a specific type of neuron, retrieving the RNA only of that cell type therefore poses a considerable challenge. Our aim was to isolate RNA specifically from Purkinje neurons in different parts of the cell body: soma (in different subcellular compartments, cytoplasm and rough endoplasmic reticulum) and dendrites.

**Methods:** In previous work, we used a technology called translating ribosome affinity purification (TRAP) to isolate the ribosome-associated transcriptome — the translatome. We modified it to target any cell type that can be specifically infected by a modified adeno-associated virus, and applied it to Purkinje cells (PCs) in the rat cerebellum. We obtained quantitative expression data in single-base-pair resolution by profiling the ribosome-associated, isolated RNA using the nanoCAGE protocol.

**Results:** Subsequent data analysis revealed the landscape of ribosome-associated RNA of PCs in different subcellular compartments: cytoplasm and rough endoplasmic reticulum in the soma. We published these results in [1]. Building on this work, we have now successfully retrieved RNA from dendrites in the same model system with replicated libraries, and thus obtained a deep sequencing using a newly developed protocol employing unique molecular identifiers (UMI) for a more precise measurement of expression.

**Conclusion:** In neurons, protein translation occurs not only in the soma but also distally in dendrites near or within the dendritic spines. This distal translation is thought to be regulated in response to external stimuli including long-term depression and memory formation. We have applied TRAP to Purkinje dendrites and sequenced the isolated RNA with an improved nanoCAGE protocol including a tagmentation step, to address the increased difficulty of sequencing from dendrites, which contain even less RNA than Purkinje cell soma.

**References**

[1] Kratz, Anton, et al. “Digital expression profiling of the compartmentalized translatome of Purkinje neurons.” *Genome research***24.8** (2014): 1396–1410.

**Competing interests**

None declared.

## P14 The evolution of imperfection and imperfection of evolution: the functional and functionless fractions of the human genome

### D. Graur

#### Biology and Biochemistry, University of Houston, Houston, USA

**Objectives:** Genomes are products of natural processes. Hence all genomes contain functional and nonfunctional parts. Here, I present a functional classification of genomic elements and estimate the functional fraction within the human genome.

**Methods:** The classification into different categories of functionality were based on the concept of selected-effect function. Intraspecific and interspecific genomic comparisons and standard evolutionary methodology were used to infer the functional fraction within the human genome.

**Results:** According to their selected-effect function, the genome is divided into functional and rubbish DNA. Functional DNA is further subdivided into literal and indifferent DNA. In literal DNA, the order of nucleotides is under selection; in indifferent DNA, only the presence or absence of the sequence is under selection. Rubbish DNA is further subdivided into junk and garbage DNA. Junk DNA neither contributes to nor detracts from the fitness and, hence, evolves under selective neutrality. Garbage DNA, on the other hand, decreases the fitness of its carriers; it exists in the genome because natural selection is neither omnipotent nor instantaneous. Each of these four functional categories can be transcribed and translated, transcribed but not translated, or not transcribed. The affiliation to a particular functional category may change during evolution: Functional DNA may become junk DNA, junk DNA may become garbage DNA, and so on; however, in the absence of prophetic powers determining the functionality or nonfunctionality of a genomic sequence must be based on its present status rather than on its potential to change in the future. Changes in functional affiliation are categorized into pseudogenes, Lazarus DNA, zombie DNA, and Jekyll-to-Hyde DNA. Intraspecific and interspecific genomic comparisons indicate that the functional fraction in the human genome ranges from 8% to 15%.

**Conclusion:** A common misconception exists according to which evolutionary processes can produce a genome that is wholly functional. Actually, evolution can only produce such a genome if and only if the effective population size is infinite, the deleterious effects of increasing genome size by even one nucleotide are considerable, and the generation time is short. Not even in the commonest of bacterial species are these conditions met. In species with small effective population sizes and long generation time, such as humans, a genome that is ~100% functional is contrary to reason.

**Competing interests**

None declared.

## P16 Species-independent identification of known and novel recurrent genomic entities in multiple cancer patients

### J. Friis-Nielsen, J. M. Izarzugaza, S. Brunak

#### Technical University of Denmark, Center for Biological Sequence Analysis, Lyngby, Denmark

##### **Correspondence:** J. M. Izarzugaza – Technical University of Denmark, Center for Biological Sequence Analysis, Lyngby, Denmark

**Objectives:** Here we present a new method for the identification of recurrent genomic entities that play a causative role in the onset of disease. Our approach is particularly amenable for the analyses high-throughput sequencing data.

Existing approaches often follow a bottom-up approach where taxonomic determination necessarily takes place before associations to disease can be determined; naturally failing to establish the causality of novel pathogens not present in reference databases.

**Methods:** To overcome this intrinsic limitation, we have developed a species-agnostic top-bottom approach that clusters sequences and identifies co-occurrence in multiple patients, associates recurrent sequences to disease and, finally, determines the taxonomic content where existing knowledge permits.

**Results:** We analysed 686 sequencing libraries from 252 cancer specimens and 56 controls. Recurrent sequences were statistically associated to biological, methodological and technical features to identify novel pathogens and contaminants stemming from laboratory reagents.

**Conclusion:** We provide examples of identified inhabitants of the healthy tissue flora, known experimental contaminants and uncharacterised sequences that co-occur with high statistical significance with disease. The latter represent a category that can only be addressed by a species-independent approach. Thus, our method helps to chart the unknown sequence-space where novel pathogens can be identified.

**Competing interests**

None declared.

## P18 Discovery of active gene modules which are densely conserved across multiple cancer types reveal their prognostic power and mutually exclusive mutation patterns

### B. S. Soibam

#### University of Houston-Downtown, Houston, USA

**Objectives:** An active module which is densely conserved across multiple gene networks exhibit strong and conserved interactions among its active member genes across all the networks. Identification of such modules across multiple species using gene networks in stem cell differentiation has provided new insights into evolutionary conserved developmental pathways and conserved pioneer factors. Similarly in cancer, such modules (if exists) will represent core common carcinogenesis-driving pathways in multiple types of cancer. These common pathways can have the same prognosis in cancers stemming from different tissues. Even though, a comprehensive amount of gene expression data for multiple types of cancer has been acquired, there is no study on comparison of several cancer gene networks. In this study, we perform local alignment of 8 cancer gene networks to identify active gene modules which are densely conserved across the cancer types. We further study the prognostic and mutation patterns within these modules.

**Methods:** We developed a computational framework to identify active modules of genes which are densely conserved across 8 different types of cancer by extending a previous tool called *neXus to allow it to work on more than 2 cancer gene networks.* We identified 174 modules of genes satisfying these *three strict criteria* – 1) member genes were differentially expressed between normal and cancer tissues (FDR < 0.05) in at least 7 cancer types of cancer of interest, 2) average fold change of 2 in the member genes across all 8 cancer types and 3) the clustering coefficient of at least 0.5 in all the 8 cancer gene networks.

**Results:** The identified modules represented some known pathways associated with multiple cancers such as the RAS pathway, but several new genes which may play important in triggering cancer in multiple types of tissues were discovered. We found that these conserved modules of genes have high prognostic power in all the 8 types of cancer of interest. We also found that these active and conserved modules exhibit similar mutually exclusive mutation patterns among the gene members and these patterns were also conserved across the majority of the types of cancer.

**Conclusion:** These findings reveal a new set of previously unknown pathways, which most likely run as a common thread during carcinogenesis in many cancer types and hence are worth investigating further for experimental validation.

**Competing interests**

None declared.

## P19 Whole exome sequencing of dysplastic leukoplakia tissue indicates sequential accumulation of somatic mutations from oral precancer to cancer

### D. Das^1^, N. Biswas^1^, S. Das^1^, S. Sarkar^2^, A. Maitra^1^, C. Panda^2^, P. Majumder^1^

#### ^1^National Institute of Biomedical Genomics, Kalyani, India; ^2^Chittaranjan National Cancer Institute, Kolkata, India

##### **Correspondence:** D. Das – National Institute of Biomedical Genomics, Kalyani, India

**Objectives:** Oral leukoplakia (OL) is the most common precancerous lesion in the oral cavity. The percentage of individuals with dysplastic OL in whom there is malignant transformation to oral squamous cell carcinoma (OSCC) is high, up to 36%. Germline and somatic copy number variations in mitochondrial DNA of OL patients have earlier been noted. We sought to test the hypothesis that about 36% of patients with the pre-cancerous lesion (OL) will possess somatic mutations in genes that are recurrently mutated in OSCC.

**Methods:** Whole exome sequencing of DNA isolated from the affected oral tissue and from peripheral blood of twelve OL patients with dysplasia, was used to profile the landscape of autosomal somatic recurrent mutations in OL and to investigate whether mutations in the genes that drive OSCC are present in OL patients or whether the mutational landscapes of OL and OSCC are largely disjoint.

**Results:** We have detected mutations in some genes that drive both oral leukoplakia and oral cancer. *TGFBR2* is recurrently mutated in OL as well as in head and neck squamous cell carcinoma (HNSCC). Some significantly mutated genes in OSCC or HNSCC, viz., *FAT1*, *NOTCH1* and *CDKN2A* are also found to be mutated in OL patients. Further, we have identified that MAPK signalling and oxidative phosphorylation (OXPHOS) pathways are significantly altered in OL patients.

**Conclusion:** We have found that the proportion of OL patients with epithelial dysplasia among whom mutations were found in the set of genes that is also recurrently mutated in OSCC/HNSCC, closely corresponds to the fraction (~36%) of patients with dysplastic leukoplakia who develop oral cancer. The leukoplakia patients recruited in this study were free of malignancy in the oral cavity; our results are, therefore, not influenced by field cancerization.

**Competing interests**

None declared.

## P21 Epigenetic mechanisms of carcinogensis by hereditary breast cancer genes

### J. J. Gruber, N. Jaeger, M. Snyder

#### Genetics, Stanford University, Palo Alto, USA

##### **Correspondence:** J. J. Gruber – Genetics, Stanford University, Palo Alto, USA

**Objectives:** BRCA2-induced breast cancers share a predominant histologic and molecular phenotype (ER+, luminal B) that distinguishes them from most sporadic breast cancers and breast cancers arising in other inherited disorders. This suggests that breast cancers arising in BRCA2-mutation carriers have essential shared properties that drive carcinogenesis and can be targeted for intervention. To investigate this observation, we performed a cell biological screen in non-transformed breast epithelial cells for phenotypes specific to BRCA2 loss-of-function.

**Methods:** Non-transformed breast epithelial cells were treated with siRNAs targeting hereditary breast cancer genes. Growth curves were obtained in complete growth media and growth factor-withdrawal medias. Whole genome sequencing and transcriptomics were performed. Functional cell biological assays including treatment with recombinant cytokines and inhibitors were performed.

**Results:** Despite the role of BRCA2 in homologous recombination-mediated DNA repair, no recurrent *de novo* mutations were recovered. Instead, we discovered a novel pathway whereby BRCA2 depletion induces strong, persistent transcriptional activation of the chemokines on chromosome 4q13 (CXCL1, CXCL3, CXCL5, CXCL8). Surprisingly, these chemokines were sufficient to stimulate EGF-independent growth of non-transformed breast cells. Furthermore, inhibitors of the receptors of the chemokines impaired cell proliferation after BRCA2 depletion.

**Conclusion:** Altogether, these findings indicate that transcriptional activation of the 4q13 chemokine locus induces an early cell-autonomous autocrine signaling pathway in BRCA2-mediated carcinogenesis that could be exploited to prevent cancer onset.

**Competing interests**

None declared.

## P22 RNA direct: a novel RNA enrichment strategy applied to transcripts associated with solid tumors

### K. Patel^1^, S. Bowman^1^, T. Davis^2^, D. Kraushaar^1^, A. Emerman^1^, S. Russello^2^, N. Henig^1^, C. Hendrickson^1^

#### ^1^Directed Genomics, USA; ^2^New England Biolabs, Ipswich, USA

##### **Correspondence:** K. Patel – Directed Genomics, USA

**Objectives:** RNA sequencing (RNA-seq) is a powerful tool used for the interrogation of transcripts that enables the analysis of gene expression and the identification of nucleotide or structural variations. RNA-seq, however, can be cost prohibitive due to the size and complexity of most transcriptomes, which require deep sequencing coverage to achieve the necessary sensitivity for a reliable analysis. By combining targeted enrichment strategies with next-generation sequencing (NGS), a subset of transcript regions or sequences can be enriched and analyzed with the resolution required for both research and clinical applications. Here, we applied a novel RNA enrichment strategy, RNA DIRECT, to the capture of targets commonly associated with solid tumors.

**Methods:** RNA DIRECT target enrichment utilizes cDNA and probe-based hybridization to capture only desired cDNA sequences with removal of off-target regions via enzymatic digestion. Targeted sequences are then ligated to NGS platform specific adaptors and amplified by PCR.

**Results:** RNA converted to cDNA was used for targeted enrichment with RNA DIRECT. Analysis of sequenced reads showed at least 97% alignment to the transcriptome with greater than 90% aligning to targeted regions. We also report the identification and detection of variants and gene fusions associated with solid tumors with high specificity and sensitivity.

**Conclusion:** The RNA DIRECT strategy provides a robust and cost effective method for the enrichment of targeted transcript sequences for the identification and detection of known or novel variants and gene fusions.

**Competing interests**

None declared.

## P23 RNA sequencing identifies gene mutations for neuroblastoma

### K. Zhang

#### Pathology, University of North Dakota, Grand Forks, USA

**Objectives:** Neuroblastomas are the most common of all malignancies in infants and the most common extracranial malignancy of childhood. With 650 new cases each year it accounts for over 15% of childhood cancer mortality. This neuroectodermally derived malignancy is most often found in the adrenal glands but can be found throughout the body in sympathetic ganglia. Though a small number of cases are familial (1-2%) with known genetic causes, most cases are sporadic with little known about what causes them or what causes the diverse outcomes of this disease.

**Methods:** The current study used transcriptome sequencing to locate possible genetic mutations from a small cohort of neuroblastoma samples. The mutations were used to construct a phylogenetic tree that demonstrated the tumor progression and predicted the outcome.

**Results:** Using this method a handful of associated mutations have been found, but few effect disease outcome. In this this study we used next generation RNA sequencing to fully sequence 249 neuroblastoma samples. Using these samples and focusing on indel mutations we located 1247 mutated genes that affect the favorable or unfavorable outcome of the disease. Due to the large number of mutations, online databases were used to identify genes associated with neuroblastomas. Comparing these genes to our sample we found a subset of genes that when mutated affected the survival rate of patients.

**Conclusion:** This study suggests the importance of a few genes that drive the progression of neuroblastomas and determine the clinical outcomes.

**Competing interests**

None declared.

## P24 Participation of SFRP1 in the modulation of TMPRSS2-ERG fusion gene in prostate cancer cell lines

### M. Rodriguez-Dorantes, C. D. Cruz-Hernandez, C. D. P. Garcia-Tobilla, S. Solorzano-Rosales

#### Oncogenomics, Inmegen, Mexico City, Mexico

##### **Correspondence:** M. Rodriguez-Dorantes – Oncogenomics, Inmegen, Mexico City, Mexico

**Objectives:** Was to determine the regulation of fusion gene *TMPRSS2-ERG* by SFRP1 protein through negative regulation of AR

**Methods**

**Cell culture**. LNCaP, VCaP and PC3 cell lines from ATCC, were grown in RPMI 1640 PrSC cell line was grown in Stromal Cell Basal medium at the same conditions mentioned above. RWPE-1 cell line, was cultured in medium. For hormonal treatment, RPMI without phenol red was used it, supplemented with Charcoal Stripped serum 5%.

**Viability assay**. Cells were plated in 96-well plates (1–2 x 10^4^ per well). 48 hours after treatment, 10mL of MTT reagent was added per well and incubated 2 hours at 37°C and 5% CO_2._ Next, medium was removed and 100mL of DMSO was added to solubilize formazan crystals. The absorbance it was readed at 575 nm wavelength.

**qRT-PCR**. Cells were plated in 24 well plates (1 x 10^5^ per well, except VCaP 2 x 10^5^). RNA extraction it was realized with RNAeasy QUIAGEN kit according to the manufacturer’s instructions. cDNA it was obtained retrotranscription assay, it was used Revert Aid Synthesis Kit according to the manufacturer’s instructions. To real time PCR assay, the following taqman expression probes from Life Technologies company were used for avery target gene : GAPDH , KLK3 , AR , TMPRSS2 , ERG , TMPRSS2-ERG , SFRP1, FZD4, Wnt1, Wnt3a and LEF1.

**Results:** We demonstrate that *TMPRSS2-ERG* is deregulated by SFRP1 protein through negative regulation of androgen receptor. Furthermore, our results indicates that androgen receptor (AR), is indirectly regulated by SFRP1 in the nucleus. We propose that the effect on fusion gene, down regulates the WNT pathway, and decreases the aggressive characteristics of the cell lines CaP. A negative effect on the TMPRSS2-ERG fusion gene by SFRP1, which was reflected in a decrease of neoplasic cell characteristics in LNCaP and VCAP PCa cells.

**Conclusion:***TMPRSS2-ERG* fusion gene is deregulated by SFRP1 protein through negative regulation of androgen receptor. Androgen receptor (AR), is indirectly regulated by SFRP1 in the nucleus. The effect on fusion gene, down regulates the WNT pathway, and decreases the aggressive characteristics of the cell lines CaP. A negative effect on the TMPRSS2-ERG fusion gene by SFRP1, which was reflected in a decrease of neoplasic cell characteristics in LNCaP and VCAP PCa cells.

**Competing interests**

None declared.

## P25 Targeted Methylation Sequencing of Prostate Cancer

### N. Jäger^1^, J. Chen^1^, R. Haile^2^, M. Hitchins^2^, J. D. Brooks^3^, M. Snyder^1^

#### ^1^Genetics, Stanford University, Palo Alto, USA; ^2^Stanford Cancer Institute, Stanford University, Palo Alto, USA; ^3^Urology, Stanford University, Palo Alto, USA

##### **Correspondence:** N. Jäger – Genetics, Stanford University, Palo Alto, USA

**Objectives:** Aggressive and potentially life-threatening prostate cancer often requires radical treatment. The many side effects that are associated with such treatments underscore the importance of accurate differentiation between these aggressive tumors and indolent prostate cancer in order to reduce overtreatment. Thus, we focus on the discovery of prognostic signatures based on changes in DNA methylation, stratifying by disease aggressiveness.

**Methods:** Whole-genome bisulfite sequencing (WGBS) is cost-prohibitive for analyzing large numbers of human cancer specimens. Further, in order to detect subtle methylation differences between cancer samples and in samples with low tumor purity, higher sensitivity is achieved through a greater read depth, further increasing sequencing costs. Therefore, we utilize a targeted bisulfite sequencing method for the comprehensive analysis of genomic regions relevant to cancer, comprising 84 megabases (~3% of the human genome), covering 3.7 million CpGs, including most RefSeq and GENCODE gene promoters, all known cancer genes, CpG islands and their shores. Up to 100-fold read depth can be achieved on average by pooling four human prostate samples on one Illumina HiSeq4000 lane.

**Results:** Of the differentially methylated regions (DMRs) we detected, the majority is hypermethylated in the aggressive versus the indolent prostate tumors. Most DMRs overlap a CpG island and are predominantly found in or close to gene promoters, as well as the promoter regions of long non-coding RNAs, and to a lesser extent within gene bodies or intergenic. Often, we find strong hypermethylation of the up- and downstream CpG islands surrounding the transcription start site (TSS), while the TSS itself stays unmethylated. Furthermore, ~70% of the hypermethylated DMRs overlap regions reported to be a bivalent promoter in various cell types from the ENCODE project. The DMRs that can be associated with RefSeq genes are enriched for transcription factors, and more than half of those contain a homeodomain, such as different members of the *HOX*, *SOX* and *FOX* gene families.

**Conclusion:** The overarching theme for DMRs called in this set of prostate cancers using a targeted deep Methylation-Sequencing approach is hypermethylation of regions within bivalent CpG islands, presenting a prognostic methylation signature that warrants further investigation.

**Competing interests**

None declared.

## P26 Mutant TPMT alleles in children with acute lymphoblastic leukemia from México City and Yucatán, Mexico

### S. Jiménez-Morales^1^, M. Ramírez^2^, J. Nuñez^3^, V. Bekker^4^, Y. Leal^5^, E. Jiménez^6^, A. Medina^7^, A. Hidalgo^8^, J. Mejía^9^

#### ^1^Cancer Genomic Laboratory, National Institute of Genomic Medicine (INMEGEN), Mexico; ^2^Biología, FES -Iztacala, UNAM, Mexico; ^3^Hospital de Pediatría, CMN SXXI, IMSS, Mexico; ^4^Investigación Médica en Inmunología, CMN La Raza, IMSS, Mexico; ^5^Diagnóstico Molecular H1N1-Influenza, UMAE-IMSS, Mérida, Yucatán, Mexico; ^6^Hematología Pediátrica, CMN La Raza, IMSS., Mexico; ^7^Hemato-Oncología, Hospital Infantil de México, Mexico; ^8^Cancer Genomics Laboratory, INMEGEN, Mexico; ^9^Coordinación de Investigación en Salud, IMSS., Mexico

##### **Correspondence:** S. Jiménez-Morales – Cancer Genomic Laboratory, National Institute of Genomic Medicine (INMEGEN), Mexico

**Objectives:** To know the frequency of the TPMT deficient alleles in children with acute lymphoblastic leukemia and healthy subjects from two Mexican populations

**Methods:** We included 813 unrelated subjects, 392 were children with ALL and 421 were healthy subjects. Genotyping of the rs1800462, rs1800460 and rs1142345 SNPs was performed by TaqMan assays. To assess the differences of the genotype and allele frequencies among groups we used the Chi-square test. Written informed consent was obtained from both ALL children’s parents and healthy participants.

**Results:** The mutant TPMT alleles were carried by 5% of the 1636 chromosomes analyzed. Overall ALL cases, 10.2% of the subjects were heterozygote for one of the tree variants and only 0.2% were homozygote to the mutant allele TPMT*3A. We did not find statistically significant differences between Mestizo and Mayan ALL or controls groups; however, 7.8 % of the ALL Mayan bore one *TPMT* mutant allele. Moreover, 2.5% of the Maya healthy subjects were homozygote to the null phenotype (TPMT*3A/TPMT*3A).

**Conclusion:** This study is the largest analysis of the *TPMT* mutant alleles performed in ALL Mexican pediatric patients [1]. Because ALL is the leading cause of childhood cancer in Mexico [2] and homozygotes *TPMT* deficient alleles subjects have high risk to develop severe and potentially fatal hematopoietic toxicity after treatment with standard doses of thiopurines [3]; *TPMT* alleles genotyping should be performed in Mexican ALL patients. Furthermore, large-scale genotype and phenotype correlation studies are needed to assess the contribution of this variants in other Mexican-Amerindian populations.

**References**

1) Taja-Chayeb L, et al. Med Oncol. 2008;25(1):56–62; 2) Pérez-Saldivar ML, et al. BMC Cancer. 2011;11:355; 3) McLeod HL, et al. Leukemia. 2000;14(4):567–72.

**Competing interests**

None declared.

## P28 Genetic modifiers of Alström syndrome

### J. Naggert, G. B. Collin, K. DeMauro, R. Hanusek, P. M. Nishina

#### Jackson Laboratory, Bar Harbor, USA

##### **Correspondence:** J. Naggert – The Jackson Laboratory, Bar Harbor, USA

**Objectives:** Alström syndrome (AS) is a progressive multi-systemic disorder caused by recessive mutations in the ciliary protein ALMS1. Amongst patients with AS, we observe variability in onset and/or severity of disease phenotypes including hearing and vision loss, obesity, hyperinsulinemia, hepatosteatitis, and cardiomyopathy, likely due to the presence of modifier genes. Like in patients, disease phenotypes in murine models of AS can be modified by the genetic background. The goal of the study was to map genetic modifier loci using an AS mouse model.

**Methods:** A genetrap in intron 13 of the mouse Alms1 gene was placed onto the C57BL/EiJ and BALB/cJ inbred background strains, respectively. To genetically dissect modifier loci of AS, we performed a phenotypic screen of 135 Alms1Gt/Gt backcross progeny from two backcrosses: ((C57BL6/Ei X Balb/cJ)F1-Alms1+/Gt X C57BL6/Ei-Alms1+/Gt) and (C57BL6/Ei X Balb/cJ)F1-Alms1+/Gt X Balb/cJ/Ei-Alms1+/Gt). DNA of the backcross progeny was typed using evenly spaced microsatellite markers throughout the genome and quantitative trait locus (QTL) analysis was performed. Recombinational fine mapping and characterization of subcongenic lines was used to refine a retinal degeneration QTL on Chr. 2.

**Results:** QTL for body weight, plasma insulin and triglyceride levels, alanine aminotransferase levels, hepatic steatosis, hepatic fibrosis, and retinal degeneration were mapped to regions on five chromosomes. The location of a major modifier locus on Chr. 2 in which the B6 allele protects *Alms1*^*Gt*/*Gt*^ retinas from rapid photoreceptor degeneration was refined to a 12 Mb region. A candidate mutation in the glutamylase TTLL9 was identified and is associated with reduced glutamylation of tubulin in Balb/cJ-*Alms1*^*Gt*/*Gt*^ retinas.

**Conclusion:** Elucidation of the genetic networks of ALMS1 may lead to a better understanding of the role of ALMS1 in metabolic and neurosensory disease and may provide novel targets for therapeutic intervention.

**Competing interests**

None declared.

## P31 Association of genomic variants with the occurrence of angiotensin-converting-enzyme inhibitor (ACEI)-induced coughing among Filipinos

### E. M. Cutiongco De La Paz^1,2^, R. Sy^3^, J. Nevado^1^, P. Reganit^3^, L. Santos^3^, J. D. Magno^3^, F. E. Punzalan^3^, D. Ona^3^, E. Llanes^3^, R. L. Santos-Cortes^4^, R. Tiongco^3^, J. Aherrera^5^, L. Abrahan^5^, P. Pagauitan-Alan^5^, the Philippine Cardiogenomics Study Group

#### ^1^National Institutes of Health, University of the Philippines, Manila, Philippines; ^2^Philippine Genome Center, University of the Philippines, Quezon City, Philippines; ^3^College of Medicine, University of the Philippines, Manila, Philippines; ^4^Department of Molecular and Human Genetics, Baylor College of Medicine , Houston, TX, United States; ^5^Philippine General Hospital, University of the Philippines, Manila , Philippines

##### **Correspondence:** E. M. Cutiongco De La Paz – National Institutes of Health, University of the Philippines, Manila, Philippines

**Objectives:** Angiotensin-converting-enzyme inhibitors (ACEIs) are among the most commonly used drugs in the management of cardiovascular disease. It is used as an antihypertensive and for the alleviation of progressive vascular injury. However, intake of ACEIs may lead to an adverse side effect, uncomfortable dry cough that occur in about 20-25% of patients. Although several genomic variants have been found to be associated with ACEI-induced coughing, there are no published data to adequately address pharmacogenetic utility among Filipino patients. This was undertaken to determine the prevalence and clinical association of candidate genomic variants among Filipinos.

**Methods:** A case–control study involving 186 unrelated patients who were taking ACEI for at least 6 months was done (101 males, 85 females; 62 cases, 124 controls). DNA from blood samples were extracted and were genotyped using customized Illumina Goldengate microarray chips for 384 gene variants.

**Results:** Results show that allelic variants of the genes ZPR1, ADAMTS7, CTB-129P6.7 and LOC157273 are significantly associated with ACEI-induced coughing (OR: 2.34, 3.120, 2.49 and 2.64, respectively, p<0.01). Using genotypic modeling, ZPR1 shows a dominant trend, while ADAMTS7 and CTB-129P6.7 manifest genotypic patterns (Cochran-Armitage test, p<0.01). Further, an intergenic loci in chromosome 4 is also significant. Interestingly, using logistic regression, in addition to ZPR1, ADAMTS7 and CTB-129P6.7, eight variants located proximal to each other in the X chromosome have been associated with the ACEI-induced coughing.

**Conclusion:** The study presents possible pharmacogenetic markers for Filipinos, as well as genomic regions of interest that may further shed light on the mechanisms of ACEI-induced coughing.

**Competing interests**

None declared.

## P32 The use of “humanized” mouse models to validate disease association of a de novo GARS variant and to test a novel gene therapy strategy for Charcot-Marie-Tooth disease type 2D

### K. H. Morelli^1,2^, J. S. Domire^3^, N. Pyne^3^, S. Harper^3^, R. Burgess^1^

#### ^1^The Jackson Laboratory , Bar Harbor, USA; ^2^Graduate School of Biomedical Sciences & Engineering, The University of Maine, Orono, USA; ^3^Center For Gene Therapy, The Research Institute at Nationwide Children’s Hospital, Columbus, Ohio, USA

##### **Correspondence:** K. H. Morelli – The Jackson Laboratory , Bar Harbor, USA

**Objectives:** Mutations in *GARS* (glycyl tRNA synthease) cause autosomal dominant Charcot-Marie-Tooth disease type 2D (CMT2D). Thirteen *GARS* variants have been previously linked to CMT2D. Recently, diagnostic whole exome sequencing revealed heterozygosity for a novel, *de novo,* 12 base-pair deletion in exon 8 of *GARS* (c.894_904del12) in a one year-old female showing symptoms including hypotonia and weakness. This variant causes an in-frame deletion of four amino acids (E299-302Qdel, referred to as “∆ETAQ”) within the catalytic domain of the enzyme and is thus likely deleterious. To validate ∆ETAQ as the causative mutation, we are currently engineering a “humanized” mouse model in which both the normally functioning human sequence of exon 8 and the mutant ∆ETAQ variant sequence of exon 8 have been introduced into the mouse genome.

**Methods:** We have successfully engineered a mouse that expresses the wild-type human sequence of *GARS* exon 8 using CRISPR/Cas genome editing technology and are currently engineering a mouse that will express the mutant sequence. Once both strains are verified we will cross them to produce a compound heterozygote with the same putatively pathogenic DNA sequences as the patient (*Gars*^∆ETAQ/+^). Once established as stocks, the *Gars*^*∆ETAQ/+*^ mice will be evaluated for features of neuropathy observed in other established mouse models of *GARS*-linked CMT2D. The *Gars*^*∆ETAQ*^ mice will also provide a humanized disease model for preclinical studies. Previous studies predict that knockdown of mutant *GARS* should be therapeutically beneficial for patients with CMT2D, provided wild type *GARS* is preserved. Therefore, we developed a gene therapy strategy that involves the allele-specific knockdown of mutant *GARS* transcripts by virally delivered RNAi. RNAi vectors designed to target the ∆ETAQ variant as well as other confirmed CMT2D-linked *GARS* variants have been developed and tested *in vitro* for knockdown efficacy and specificity.

**Results:** The results of our *in vitro* studies confirm that we have developed several RNAi sequences that specifically target several *GARS* variants but not wild type *GARS.*

**Conclusion:** Success with this novel gene therapy approach will provide a promising avenue for treatment of CMT2D and other dominantly inherited neuromuscular diseases.

**Competing interests**

None declared.

## P34 Molecular regulation of chondrogenic human induced pluripotent stem cells

### M. A. Gari^1^, A. Dallol^2^, H. Alsehli^2^, A. Gari^2^, M. Gari^2^, A. Abuzenadah^2^

#### ^1^Medical Laboratory Technology, Saudi Arabia; ^2^Center of Innovation in Personalized Medicine, King Abdulaziz University, Jeddah, Saudi Arabia

##### **Correspondence:** M. A. Gari – Medical Laboratory Technology, Saudi Arabia

**Objectives:** Human induced pleuripotent stem cells (hiPSC) are a promising source for chondrogenic stem cells. Sequential differentiation of hiPSC provides a platform for dissecting the molecular pathways associated with chondrogenesis *in vivo* and could reveal targets for better control of chondrocyte fate for cartilage repair applications. The aim of this study was to use next generation sequencing (NGS) to investigate the transcriptome and the methylome of chondrogenic hiPSCs.

**Methods:** The hiPSC line (C19) was derived through reprogramming of human dermal fibroblasts using viral vectors expressing Oct4, Sox2 and Klf4. A protocol of sequential growth factors including Activin A, FGF2 and BMP4 was used to drive the formation of chondroprogenitors directly from hiPSC colonies. The chondrogenic hiPSCs were characterised exhaustively by tissue engineering, histochemical analysis and biochemical analysis. The transcriptome of undifferentiated hiPSCs, hiPSC derived chondrocytes and native chondrocytes was interrogated utilizing RNASeq on the SOLiD 5500 XL platform where the polyA fraction was sequenced at a coverage level of at least 25 million reads.

**Fig. 7 (abstract P34).**
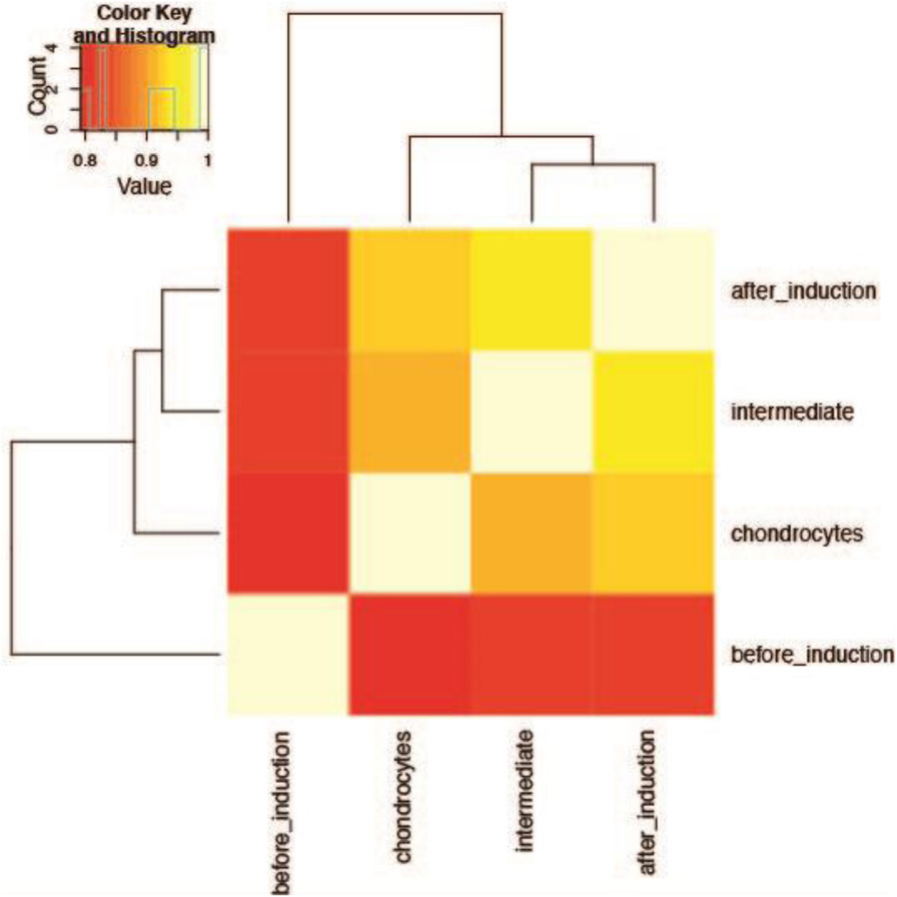
ᅟ

**Results:** Differential gene expression revealed the induction of several collagen genes including type 1 to type 12, type 14 and type 18 during transition from the pluripotent state to the chondrogenic state (Fig. 7). Collagen regulatory genes such as PCOLCE which drives the endopeptidase cleavage of procollagen as well as regulators of collagen glycosylation were upregulated. The expression of various fibroblasts growth factors (FGFs) including FGF11, FGFR2 and insulin growth factor2 (IGF2) was upgregulated as well as the Wnt induced secreted protein, WISP2. Mitotic genes were downregulated in derived and differentiated chondrocytes. Integrated methylome and transcriptome analysis revealed the step-wise differentiation process.

**Conclusion:** NGS analysis demonstrated the recapitulation of early events in cartilage development during hiPSC chondrogenesis. The upregulation of many members of the collagen family indicate the intricate nature of collagen expression during chondrogenesis. Further analysis of nonchondrogenic targets may reveal novel pathways for controlling the fate of chondrogenic hiPSCs.

**References**

1. Pearle, A.D., S.A. Warren Rf Fau - Rodeo, and S.A. Rodeo, *Basic science of articular cartilage and osteoarthritis.*

**Competing interests**

None declared.

## P35 Molecular profiling of hematologic malignancies: implementation of a variant assessment algorithm for next generation sequencing data analysis and clinical reporting

### M. Thomas^1^, M. Sukhai^1^, S. Garg^1^, M. Misyura^1^, T. Zhang^1^, A. Schuh^2^, T. Stockley^1^, S. Kamel-Reid^1^

#### ^1^Advanced Molecular Diagnostics Laboratory, Canada; ^2^Princess Margaret Cancer Centre, Toronto, Canada

##### **Correspondence:** M. Thomas – Advanced Molecular Diagnostics Laboratory, Canada

**Objectives:** Next generation sequencing (NGS) can be used to identify clinically relevant variants for accurate diagnosis, prognostic risk stratification, and identification of therapeutic targets for genotype-matched trials in cancer. Our objective was to design methods for efficient prioritization of variants for interpretation and reporting. We describe a triaging algorithm to identify clinically relevant variants from tumour-only analysis of NGS in hematological malignancies.

**Methods:** Blood or bone marrow DNA from 260 patients recruited in the Princess Margaret Cancer Centre’s Advanced Genomics in Leukemia (AGILE) trial were profiled using the 54 gene TruSight Myeloid Sequencing Panel (Illumina). Variants were called using the Illumina MiSeq Reporter (MSR) software. Data were uploaded into a commercially available tool, Cartagenia Bench NGS, for filtering and analysis.

**Results:** Of all variants detected by NGS (median 427, range 338–643 variants/case), 35% (median 150, range 125–172 variants/case) passed all MSR quality criteria. Applying a variant allele frequency threshold refined the data to 7.4% of the original dataset (median 30, range 16–48 variants/case). Reporting was restricted to well-covered, exonic nonsynonymous, intronic splice site, and known pathogenic synonymous variants, resulting in a median of 4 variants/case for manual review (range 0–13). When combined with our dataset of >600 interpretations across 8 hematological malignancies, this approach enabled rapid review and interpretation of previously known variants, and an effective system to prioritize novel variants in order of clinical actionability (Sukhai et al., 2015). We excluded variants with high germline population frequencies (median 50, range 42–62 variants/case) through the use of multiple reference population databases.

**Conclusion:** We describe our approach to prioritize NGS derived variants, based on data quality, functional effects, allele frequency, coverage depth, and coding effects. This approach iteratively utilizes our lab-developed variant knowledge base, and enables us organize and use variant interpretations to generate clinical reports.

**References**

Sukhai, M. A. *et al.* A classification system for clinical relevance of somatic variants identified in molecular profiling of cancer. *Genet Med*, doi:10.1038/gim.2015.47 (2015).

**Competing interests**

None declared.

## P36 Accessing genomic evidence for clinical variants at NCBI

### S. Sherry, C. Xiao, D. Slotta, K. Rodarmer, M. Feolo, M. Kimelman, G. Godynskiy, C. O’Sullivan, E. Yaschenko

#### NCBI, NIH, Bethesda, USA

##### **Correspondence:** S. Sherry – NCBI, NIH, Bethesda, USA

**Objectives:** NCBI provides many resources for evaluating and declaring evidence of pathogenicity for an increasing number of human sequence variants. Primary details about a genetic test’s analytical validity and clinical utility are reported in the Genetic Test Repository, and evidence for inferences of variant pathogenicity are summarized in ClinVar. NCBI has developed several tools that integrate these high level records with the more basic factual data describing them as population level variants (dbSNP/dbVar), and deeper still as individual-level observations with called genotypes (the NCBI Genotype Server), phenotypes (dbGaP), whole genome/exome sequences (SRA) and finally as positions on the reference genome (GRC human reference assemblies).

**Methods:** Traversing the connections between variant-level records, e.g. GTR, ClinVar and dbSNP, and individual-level data (e.g. genotypes, sequences, samples, and phenotypes) is a computationally intensive activity, and NCBI has developed several new services to pre-compute these relationships and permit users to quickly move from summary records to individual level data.

**Results:** This presentation will introduce several of these services including allele registry, the ClinVar beacon search, and the dbGaP genome browser with particular emphasis on how users can access and review individual level data for clinical variants or *ad hoc* genomic positions of particular interest.

**Conclusion:** Examples of use include the review of ClinVar records by expert panels, identify research into the existence of specific sequence alleles, automatic notification when new data for specific potential alleles of interest are submitted to NCBI, confirmation of variant properties during manuscript review, and research in general questions of human genetic architecture.

**Competing interests**

None declared.

## P37 NGS-SWIFT: a cloud-based variant analysis framework using control-accessed sequencing data from DBGAP/SRA

### C. Xiao, E. Yaschenko, S. Sherry

#### National Institutes of Health, Bethesda, USA

##### **Correspondence:** C. Xiao – National Institutes of Health, Bethesda, USA

**Objectives:** Genetic variation analysis plays an important role in elucidating the causes of various human diseases. The drastically reduced costs of genome sequencing driven by next generation sequence technologies now make it possible to analyze genetic variations with hundreds or thousands of samples simultaneously, but with the cost of ever increasing local storage requirements. The tera- and peta-byte scale footprint for sequence data imposes significant technical challenges for data management and analysis, including the tasks of collection, storage, transfer, sharing, and privacy protection. Currently, each analysis group must download all the relevant sequence data into a local file system before variation analysis is initiated. This heavy-weight transaction not only slows down the pace of the analysis, but also creates financial burdens for researchers due to the cost of hardware and time required to transfer the data over typical academic internet connections.

**Methods:** To overcome such limitations and explore the feasibility of analyzing control-accessed sequencing data in cloud environment while maintaining data privacy and security, here we introduce a cloud-based analysis framework that facilitates variation analysis using direct access to the NCBI Sequence Read Archive through SRA Toolkit, which allows the users to programmatically access data housed within SRA with encryption and decryption capabilities and converts it from the SRA format to the desired format for data analysis.

**Results:** A customized machine image (ngs-swift) with preconfigured tools, including SRA Toolkit and NGS Software Development Kit, and resources essential for variant analysis has been created for instantiating an EC2 instance or instance cluster on Amazon cloud. Performance of this framework has been evaluated using dbGaP study phs000710.v1.p1, and compared with that from traditional analysis pipeline, and security handling in cloud environment when dealing with control-accessed sequence data has been addressed. We demonstrate that with this framework, it is cost effective to make variant calls without first transferring the entire set of aligned sequence data into a local storage environment.

**Conclusion:** This direct data access approach using NCBI SRA Toolkit from cloud for next generation sequencing analysis is more cost-effective in terms of time and disc spaces being used for the analysis, and thus will accelerate variation discovery using control-accessed sequencing data.

**Competing interests**

None declared.

## P38 Computational assessment of drug induced hepatotoxicity through gene expression profiling

### C. Rangel-Escareño, H. Rueda-Zarate

#### Computational Genomics, National Institute of Genomic Medicine, Mexico City, Mexico

##### **Correspondence:** C. Rangel-Escareño – Computational Genomics, National Institute of Genomic Medicine, Mexico City, Mexico

**Objectives:** Liver is the primary organ responsible for drug metabolization process. Many currently and normally used drugs could affect the liver adversely in any combination of the reactions described. Gene expression profiling can be used to identify the mechanisms that underlie the potential toxicity of chemicals. This technology has also been applied to identify biomarkers of toxicity to predict potential hazardous chemicals. We propose a strategy that allows to combine models (Hu, Rat), protocols (iVV, iVT), dosages (None, Low, Med, High) and time points to correlate gene profiles to levels of toxicity of 131 compounds, mainly medical drugs and their effect in the liver.

**Methods:** The strategy involves differential expression by group contrasts or ranking genes based on absolute or relative amounts of change over time as a function of the drug concentration in relation to their replicate variances. Once genes are classified, a series of class discovery methods are applied to identify and analyze patterns of chemical structure, gene profiles over time or gene profiles by compound dose. We used the Japanese toxicogenomics project, data contains a collection of 17,657 Affymetrix^TM^ microarrays from human in vitro and animal samples. Data are pre-processed in the R statistical environment using a collection of libraries from Bioconductor.

**Results:** Due to the dynamic nature of the data, we implemented a time course analysis for each species in all dosages and for all compounds. For every subset, genes are classified according to a multivariate empirical Bayes statistic for replicated microarray time course data. Genes were ranked based on large absolute or relative amounts of change over time as a function of the drug concentration in relation to their replicate variances. A hierarchical clustering analysis to identify patterns of gene profiles was performed. Genes were selected based on top ranking according to their MB statistic.

**Conclusion:** Our approach involves the development of a pipeline that involves involves analyses that are hypothesis free or hypothesis driven. Hepatotoxicity caused by drugs, in particular idiosyncratic reactions, is a major challenge to the pharmaceutical industry and physicians. The application of new technologies, such as genomics, offers the potential to identify risk factors and clarify the pathogenesis of idiosyncratic hepatotoxicity. Even with these technologies the data mining process is the real challenge.

**Competing interests**

None declared.

## P40 Flowr: robust and efficient pipelines using a simple language-agnostic approach;ultraseq; fast modular pipeline for somatic variation calling using flowr

### S. Seth^1^, S. Amin^2^, X. Song^1^, X. Mao^2^, H. Sun^3^, R. G. Verhaak^2^, A. Futreal^2^, J. Zhang^1^

#### ^1^Institute of Applied Cancer Science, USA; ^2^Genomic Medicine, University of Texas; ^3^MD Anderson Cancer Center, Houston, USA

##### **Correspondence:** S. Seth – Institute of Applied Cancer Science, USA

**Objectives:** Bioinformatics analyses have increasingly become compute intensive processes, with lowering costs of data production and increasing numbers of samples. Each laboratory spends time creating and maintaining a set of pipelines, which may not be robust, scalable, or efficient. Further, the existence of different computing environments across institutions hinders both collaboration and the portability of analysis pipelines.

**Methods:** Flowr is a robust and scalable framework for designing and deploying computing pipelines in an easy-to-use fashion. It implements a scatter–gather approach using computing clusters, simplifying the concept to the use of five simple terms (in submission and dependency types). Most importantly, it is flexible, such that customizing existing pipelines is easy, and since it works across several computing environments (LSF, SGE, Torque, and SLURM), it is portable.

**Results:** Ultraseq; Using flowr’s framework we have created a flexible pipeline for somatic variant calling. This aligns raw reads, splitting the processing across multiple nodes of a computing cluster. Results are gathered, creating a merged bam; which is then split according to the contigs in the genome. Further steps including de-duplication, indel-realignment and base-quality recalibration and variant calling are performed at the contig level, using a scatter–gather approach. This enables complete processing of a typical whole-exome in about two hours, and whole-genome in a few hours (depending on the library size).

**Conclusion:** Flowr follows the “design once” principle, enabling the user to develop robust, portable pipelines that can be run on a host of computing platforms. Further, the same pipeline can be run on a local machine, computing cluster, or cloud-based environment. With automatic logging of each step and the preservation of the exact commands run to produce the output, the system allows users to generate an easy-to-use, efficient, and reproducible analysis pipeline. Ultraseq provides a fast and robust and modular pipeline for somatic variation calling. This enables users to easily add new tools and methods are they are released, and possibly share with other users in a format, which is robust, efficient and portable.

Availability: http://docs.flowr.space

**Competing interests**

None declared.

**Fig. 8 (abstract P40).**
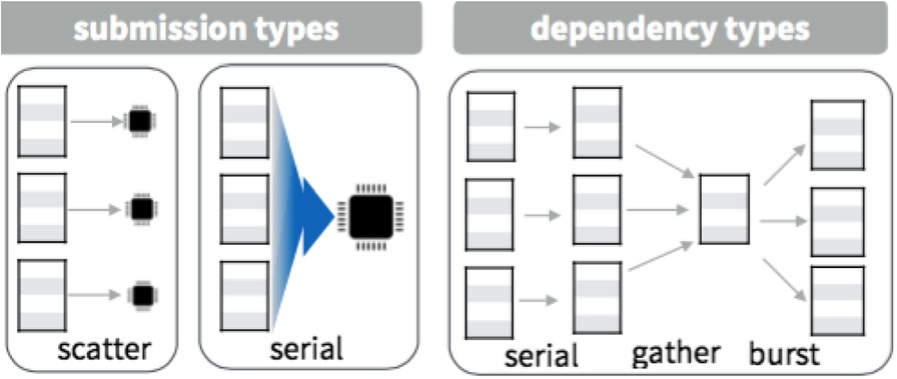
ᅟ

## P41 Applying “Big data” technologies to the rapid analysis of heterogenous large cohort data

### S. J. Whiite^1^, T. Chiang^1^, A. English^1^, J. Farek^1^, Z. Kahn^1^, W. Salerno^1^, N. Veeraraghavan^1^, E. Boerwinkle^2^, R. Gibbs^1^

#### ^1^Human Genome Sequencing Center, Baylor College of Medicine, USA; ^2^Human Genetics Center, University of Texas Health Science Center at Houston, Houston, USA

##### **Correspondence:** S. J. Whiite – Human Genome Sequencing Center, Baylor College of Medicine, USA

**Objectives:** Analysis of large cohort genomic data is a complex task, typical bioinformatics approaches use collections of scripts and R code, this becomes difficult to manage as sample numbers grow to 10’s of thousands and file sizes grow to terabytes. We applied “Big data” technologies to the analysis of 578 HI-seq X whole genomes (WGS) and 10,913 exomes (WES), in both cases we QC’d and integrated billions of variant calls from Atlas and GATK to provide concordant and consensus genotypes.

**Methods:** Raw variant data was pre-processed to produce key value pairs; keys representing variants and values representing sample specific data stored as JSON. Using a modest Hadoop test cluster (88 cores 512 Gb memory 7tb disk), we imported the data into Hbase tables. WGS data was stored as 578 columns, one column per sample.WES data was stored as a single column due to the large number of samples.

QC and analysis were performed by first building normalised summary tables in Hive, this process required a full Hbase table scan and took several hours to complete. However, once the data was processed, we were able to perform rapid analysis (minutes) on both the WGS and WES data sets using identical SQL queries regardless of how the underlying data was structured.

In this way we were able to perform QC filtering such as Ti/Tv, mappability, monomorphic sites, missingness rates and Hardy-Weinberg on both the variant and sample level, easily excluding/including sub cohorts for different tests.

**Results:** Our queries were verified by comparison to 2 independent sets of QC statistics for the 578 WGS samples, once we had confirmed that we could exactly reproduce these numbers we were then able to quickly apply the same QC steps to the much larger 10,913 WES data set. Because the tests could be performed rapidly it became practical to iteratively tune parameters, in this way we were able to modify the Atlas genotype boundaries so as to maximise the numbers of concordant genotypes with GATK.

**Conclusion:** By leveraging the power of map-reduce and the rich ecosystem of tools around Hadoop we were able to streamline the analysis of these large genomic data sets, rapidly processing billions of genotypes using simple SQL queries to provide accurate, reproducible QC statistics without the need to track and manage thousands of files.

**Competing interests**

None declared.

**Table 2 (abstract P41).** Example of summary statistics comparing Atlas genotype calls with differing allele fraction thresholds with GATK calls.

**Genotype**	**GATK**	**ATLAS (ORIGINALS)**	**ATLAS** **DP>=10** **0.25<0/1>0.75**	**ATLAS** **DP>=10** **0.1<0/1>0.9**	**ATLAS** **DP>=10** **0.1<0/1>0.75**
0/0	1,793,394,356	1,788,668,240	1,778,668,240	1,778,668,240	1,778,668,240
0/1	138,744,203	69,153,666	112,165,710	147,763,797	124,056,267
1/1	79,407,733	60,398,312	73,417,546	49,710,016	73,423,295
Het / hom	1.75	1.14	1.53	2.97	1.69
Total	2,011,546,292	1,908,220,218	1,964,251,496	1,976,142,053	1,976,147,802
**Concordant Genotypes**	**GATK + ATLAS (ORIGINALS)**	**GATK + ATLAS (0.25<0/1>0.75)**	**GATK + ATLAS (0.1<0/1>0.9)**	**GATK + ATLAS (0.1<0/1>0.75)**	
0/0	914,737,638	1,021,734,445	1,050,682,661	1,049,337,470	
0/1	39,133,931	72,422,398	74,278,418	75,570,661	
1/1	17,017,045	45,077,505	33,993,884	45,368,697	
Het/hom	2.30	1.61	2.19	1.67	
Total	970,888,614	1,139,234,348	1,158,954,963	1,170,276,828	

## P42 FANTOM5 web resource for the large-scale genome-wide transcription start site activity profiles of wide-range of mammalian cells

### T. Kasukawa^1^, M. Lizio^1^, J. Harshbarger^1^, S. Hisashi^1,2^, J. Severin^1^, A. Imad^1^, S. Sahin^1^, T. C. Freeman^3^, K. Baillie^3^, A. Sandelin^4^, P. Carninci^1^, A. R. R. Forrest^1^, H. Kawaji^1,2,5^, The FANTOM Consortium ^1^

#### ^1^Center for Life Science Technologies, RIKEN, Yokohama, Japan; ^2^Preventive Medicine and Diagnosis Innovation Program, RIKEN, Wako, Japan; ^3^The Roslin Institute and Royal (Dick) School of Veterinary Studies, University of Edinburgh, Edinburgh, United Kingdom; ^4^Department of Biology & Biotech Research and Innovation Centre, University of Copenhagen, Copenhagen, Denmark; ^5^Advanced Center for Computing and Communication, RIKEN, Yokohama, Japan

##### **Correspondence:** T. Kasukawa – Center for Life Science Technologies, RIKEN, Yokohama, Japan

**Objectives:** To identify genome-wide transcription start site activities in various mammalian cells, a worldwide collaborative project, FANTOM5 (Functional Annotation of Mammalian Cells 5) was organized. In this project, diverse range of mammalian samples (~2000 for human and ~1200 for mouse, including primary cells, cancer cell lines, tissues, and transiting cells in time courses) was profiled to obtain promoter-level transcriptional atlas using high-throughput sequencers. To provide this large-scale data collection to the scientific community, we developed an integrated web resource, the FANTOM5 web resource.

**Methods:** To support various sort of inspections and analyses, the web resource contains several tools. SSTAR (Semantic catalogue of Samples, Transcription initiation And Regulators) provides a wide range of analysis results as well as detailed information of individual samples; ZENBU, a data integration, data processing, and expression enhanced visualization system designed for big data genomics projects, provides interactive way to inspect the entire promoter activities measured in FANTOM5 interactively; and Table Extraction Tool (TET) provides an easy-to-use interface to download subsets of the overall FANTOM5 expression table in an efficient way. A BioMart instance and a track hub for UCSC genome browser enable us to access our TSS resources with widely used interfaces. Furthermore, PrESSto provides an interface to browse human enhancers identified in the FANTOM5 project, and Biolayout enable us to visualize biological states in a three dimensional expression space with interactive interface.

**Results:** The web resource is accessible from the FANTOM5 portal page: http://fantom.gsc.riken.jp/5/.

**Conclusion:** The resource is continuously updating, for example, the data remapped to the recent genomes was added. The FANTOM5 web resource is a gateway to access the large expression atlas in mammalian and we describes our challenges to provide large-scale genomic data to the scientific community from multiple aspects.

**Competing interests**

None declared.

## P43 Rapid and scalable typing of structural variants for disease cohorts

### W. Salerno^1^, A. English^1^, S. N. Shekar^2^, A. Mangubat^2^, J. Bruestle^2^, E. Boerwinkle^1,3^, R. A. Gibbs^1^

#### ^1^Human Genome Sequencing Center, Baylor College of Medicine, Houston, Texas, USA; ^2^Spiral Genetics, Seattle, Washington, USA; ^3^Human Genetics Center and Department of Epidemiology, UT School of Public Health, Houston, Texas, USA

##### **Correspondence:** W. Salerno – Human Genome Sequencing Center, Baylor College of Medicine, Houston, Texas, USA

**Objectives:** For studies such as the Alzheimer’s Disease Sequencing Project and the CHARGE Consortium, disease complexity manifests as data heterogeneity, requiring informatics that can additively scale to thousands of samples and analytics that go beyond identifying small variants in capture data. Specifically, whole-genome sequencing (WGS) can assess large, complex genomic variation in both coding and non-coding regions. At scale, the challenge of evaluating these structural variants (SVs) becomes the “N+1” problem of incrementally adding samples without having to perpetually reevaluate petabytes of population read data stored in BAM files.

**Methods:** The Biograph Analysis Format (BAF) is a novel method of indexing NGS data that allows rapid, at-scale queries by coordinate and genomic sequence. A BAF of HiSeq X 30x WGS data is 8.3 Gb, 95% smaller than the corresponding BAM. Generated from the BAM in 14 hours, the BAF can be queried up to one million times a second. Querying by both coordinate and sequence is particularly applicable to SV typing: the presence and genotype of an SV can be evaluated using coverage, mismapped reads, and characteristic sequences. This flexibility allows BAF queries to capture SV diversity. Together, fast querying of small files with reasonable compute requirements provides an N+1 solution.

**Results:** To evaluate the BAF, we indexed the HS1011 reference genome sequenced with 60x coverage on the HiSeq X. The HS1011 individual has previously been characterized for SV content, identifying ~10,000 high-confidence SVs from ~30,000 putative sites. These sites were BAF-queried in less than 60 seconds. SV-typing these sites across 10,000 HiSeq X WGS samples in BAF would require less than 100 TB, 166 CPU hours, and 2.5 total hours. Because the BAF can be batched across individuals, query time grows less than linearly with the number of individuals.

**Conclusion:** We will describe how the BAF API allows users to query SV data, how these queries inform SV typing, and how BQF typing performs on a pilot set of 100 30x HiSeq X samples from a disease cohort, providing counts and allele frequencies for multiple SV types.

**Competing interests**

None declared.

## P44 Polymorphism of glutathione S-transferases and sulphotransferases genes in an Arab population

### A. H. Salem^1^, M. Ali^2^, A. Ibrahim^3^, M. Ibrahim^4^

#### ^1^Anatomy, Arabian Gulf University, Manama, Bahrain; ^2^Biochemistry, Arabian Gulf University, Manama, Bahrain; ^3^Central Laboratory, Ministry of Science and Technology, Sudan; ^4^College of Animal Production Science and Technology, Sudan University of Science and Technology, Khartoum, Sudan

##### **Correspondence:** A. H. Salem – Anatomy, Arabian Gulf University, Manama, Bahrain

**Objectives:** Glutathione S-transferases (GSTs) play a major role in the detoxification of various compounds. Similarly, human sulfotransferases (SULTs) are involved in the metabolism of many drugs, xenobiotics, neurotransmitters and hormones. Polymorphic variants in GST and SULT1A1 genes were reported for different populations. The objectives of this study were to determine the frequencies of GSTM1 and GSTT1 null genotypes, and the genotype and allele frequencies of SULT1A1 (G638A) gene polymorphism in the Sudanese population.

**Methods:** GST genotyping was carried out using multiplex PCR while SULT1A1 genotyping was done using PCR-RFLP. Study population included 114 (GST genotyping) and 142 (SULT1A1 genotyping) unrelated healthy Sudanese subjects.

**Results:** The prevalence of GSTM1 and GSTT1 deletion homozygosity were 54.7% and 42.1%, respectively. The genotype frequencies of SULT1A1 gene polymorphism showed that 40.8% of Sudanese were homozygous for the GG genotype, 50.7% were heterozygous for the GA genotype and 8.5% were homozygous for the AA genotype. The frequencies of the SULT1A1*1 and SULT1A1*2 alleles in the SULT1A1 Gene were found to be 0.66 and 0.34, respectively. There are no significant differences in allelic distribution of GSTM1 gene between the Sudanese and other ethnic groups except for sub-Saharan Africans. As regards the allelic distribution of GSTT1 gene, the Sudanese population is similar to sub-Saharan Africans but significantly different from Europeans. Combined analysis of both genes revealed that 24.6% of Sudanese harbor the deleted genotype of both genes and it is the highest reported so far for an Arab and African population. The frequency of the SULT1A1*2 allele did not differ significantly from Caucasians and African-Americans, but it was much higher than in Asians.

**Conclusion:** This is the first study that addresses polymorphism of GST and SULT1A1 genes in Sudanese. We provide a reference database of allelic frequencies of the genotypes of these genes among Sudanese.

**Competing interests**

None declared.

## P46 Genetic divergence of CYP3A5*3 pharmacogenomic marker for native and admixed Mexican populations

### J. C. Fernandez-Lopez^1^, V. Bonifaz-Peña^1^, C. Rangel-Escareño^1^, A. Hidalgo-Miranda^2^, A. V. Contreras^3^

#### ^1^Computational Genomics, Nacional de Medicina Genomica, Mexico City, Mexico; ^2^Cancer Genomics Laboratory, Nacional de Medicina Genomica, Mexico City, Mexico; ^3^Nutrigenetics and Nutrigenomics Laboratory, Instituto Nacional de Medicina Genomica, Mexico City, Mexico

##### **Correspondence:** J. C. Fernandez-Lopez – Computational Genomics, Nacional de Medicina Genomica, Mexico City, Mexico

**Objectives:** The frequency distribution of pharmacogenomics markers across the populations in the worldwide can differ in magnitude or be absent depending on the population being assessed. The CYP3A5*3 (rs776746) is the most frequent and well-studied variant allele of *CYP3A5*. This polymorphism have been related to the guidelines regarding the use of pharmacogenomic tests in dosing of immunosuppressive Tacrolimus drug published in Clinical Pharmacology and Therapeutics by the Clinical Pharmacogenetics Implementation Consortium (CPIC). The CYP3A5*3 genotype is polymorphic in several continental and large populations, for example the minor allele frequency (MAF) of rs776746 marker in Europeans and Africans populations is opposite, while *A* allele is the minor (0.036) for Europeans *G* allele is the minor (0.15) in Africans. However, this pharmacogenomic marker is not well-studied in the admixed and Native American populations. The objective in this study is evaluate the distribution of allele frequencies for the CYP3A5*3 variant across the admixed and Native American (Mexican) populations.

**Methods:** Genotypes for *rs776746* from the Mexican Genome Diversity Project database were analyzed. Allele frequencies were calculated for 4 Native American-Mexican populations located in north, central and southwest Mexico territory plus 370 Mexican mestizos and 60 European, 60 African, 90 East Asian from the HapMap project. The *Fst* statistic was analyzed as measure of the degree of genetic differentiation pairwise populations.

**Results:** Extreme genetic differentiation among HapMap populations for was observed for CYP3A5*3 (rs776746) as one would expect, particularly for the comparisons of African and European ancestry populations. However, the largest *Fst* statistic and allele frequencies values was found among Native Americans. For example, the allele frequency of CYP3A5*3 allele is more than 6-fold higher in Tepehuanes (0.43) than Zapotecas (0.07) with *Fst* value of 0.1746 (largest divergence).

**Conclusion:** We identified novel CASR variants that have a potential to be related to serum calcium levels in Korean population. Inter-ethnic differences were suggested in some associated SNPs. Given the significant role played by calcium in many diseases and cell signaling, further studies with more East Asian subjects or meta-analyses on them may enable validation of our results and identification of novel genetic loci associated with serum calcium levels.

**Competing interests**

None declared.

**Table 3 (abstract P46).** Genetic differentiation among Native and admixed Mexican populations

**POP1_name**	**POP2_name**	**POP1_freq** **rs776746**	**POP2_freq** **rs776746**	***Fst*** **(Pop1vsPop2)**
ZAPOTECAS	CEU(hapmap)	0.07143	0.036	0.0062
ZAPOTECAS	YRI(hapmap)	0.07143	0.845	0.6026
ZAPOTECAS	JPT+CHB	0.07143	0.2921	0.0819
ZAPOTECAS	MEXICAN MESTIZOS	0.07143	0.2263	0.0473
ZAPOTECAS	MIXTECOS	0.07143	0.23333	0.0507
ZAPOTECAS	MAYAS	0.07143	0.1481	0.0150
ZAPOTECAS	TEPEHUANES	0.07143	0.4348	0.1746
MAYAS	CEU(hanmap)	0.1481	0.036	0.0376
MAYAS	YRI(hapmap)	0.1481	0.845	0.4857
MAYAS	JPT+CHB	0.1481	0.2921	0.0302
MAYAS	MIXTECOS	0.1481	0.23333	0.01188
MAYAS	TEPEHUANES	0.1481	0.4348	0.0995
MAYAS	MEXICAN MESTIZOS	0.1481	0.2263	0.0100
TEPEHUANES	CEU(hanmap)	0.4348	0.036	0.2209
TEPEHUANES	YRI(hapmap)	0.4348	0.845	0.1826
TEPEHUANES	JPT+CHB	0.4348	0.2921	0.0220
TEPEHUANES	MIXTECOS	0.4348	0.23333	0.0456
TEPEHUANES	MEXICAN MESTIZOS	0.4348	0.2263	0.0456
MIXTECOS	CEU(hanmap)	0.23333	0.036	0.0835
MIXTECOS	YRI(hapmap)	0.23333	0.845	0.3764
MIXTECOS	JPT+CHB	0.23333	0.2921	0.0045
MIXTECOS	MEXICAN MESTIZOS	0.23333	0.2263	0.001
MEXICAN MESTIZOS	CEU(hanmap)	0.2263	0.036	0.0795
MEXICAN MESTIZOS	YRI(hapmap)	0.2263	0.845	0.3847
MEXICAN MESTIZOS	JPT+CHB	0.2263	0.2921	0.0056

## P47 Whole exome sequence meta-analysis of 13 white blood cell, red blood cell, and platelet traits

### L. Polfus, CHARGE and NHLBI Exome Sequence Project Working Groups

#### Human Genetics Center, University of Texas Health Science Center, Houston, USA

##### **Correspondence:** L. Polfus – Human Genetics Center, University of Texas Health Science Center, Houston, USA

**Objectives:** Blood cell counts and erythrocyte indices are clinically important indicators of a variety of disease states. The ability to detect and include novel rare variants in gene-based tests is best accomplished via sequencing.

**Methods:** In order to identify novel genes influencing hematologic traits, we analyzed whole exome sequencing data in 15,459 participants of 6 population-based cohort studies for 13 traits in European descent (EU) and African Americans (AA), analyzed together and separately, in fixed-effects meta-analyses. We analyzed variants individually and aggregated functional and loss-of-function (LOF) variants with MAF<5% within genes using SKAT and T5 burden tests. CRISPR/Cas9 genome editing was performed to functionally follow up a replicated novel finding.

**Results:** Discovery phase associations identified 4 novel gene-based results and 3 novel single variants meeting exome-wide Bonferroni corrected thresholds (P<2.6x10^−6^ and P<1.6x10^−7^). Rare functional variants in *MYOM2* were associated with mean corpuscular hemoglobin concentration (SKAT P_EU+AA_=2.2x10^−6^). In EUs, rare variants in *MRPL43* were associated with hemoglobin (T5 P_EU_=1.2x10^−6^). LOF Variants in *MMACHC* were associated with hemoglobin (SKAT P_EU_=1.3x10^−6^). *MMACHC* regulates vitamin B12 metabolism and known variants cause a Mendelian syndrome including anemia. In AAs, rare functional variants in *ACTN4* were associated with mean corpuscular volume (SKAT P_AA_=1.5x10^−6^). *ACTN4* variants were previously associated with focal segmental glomerulosclerosis of the kidney. Among the single variant tests, we found low-frequency variant rs9656446 (MAF=0.03, P_EU+AA_=1.5x10^−7^) in *AGBL3* to be associated with basophil count. A common variant in *CPS1* (rs1047891, MAF=0.33, P_EU+AA_=5.7x10^−8^) was associated with platelet count. We also identified an association of platelet count with a low-frequency (MAF=0.009) synonymous variant (rs150813342, P_EU_=4.7x10^−8^) in *GFI1B,* a gene known to cause gray platelet syndrome. *In silico* algorithms predict rs150813342 to affect exon 5 splicing. Replication in up to 52,024 individuals were significantly associated with lower platelet count for *CPS1* rs1047891 (MAF_EU+AA_=0.328, P=1.02x10^−4^) and *GFI1B* rs150813342 (P=5.71x10^−21^).

**Conclusion:** In this exome sequencing study, we identified a novel low frequency loci. We demonstrate an alternative splicing mechanism by which the *GFI1B* rs150813342 variant suppresses formation of a GFI1B isoform that preferentially promotes megakaryocyte differentiation and platelet production.

**Competing interests**

None declared.

## P48 Association of adipoq gene with type 2 diabetes and related phenotypes in african american men and women: The jackson heart study

### S. Davis^1^, R. Xu^1^, S. Gebeab^1^, P Riestra^1^, A Gaye^1^, R. Khan^1^, J. Wilson^2^, A. Bidulescu^3^

#### ^1^Cardiovascular Section/Metabolic, Cardiovascular and Inflammatory Disease Genomics Branch, National Human Genome Research Institute/National Institutes of Health, Bethesda; ^2^University of Mississippi Medical Center, Jackson; ^3^Department of Epidemiology and Biostatistics, Indiana University School of Public Health, Bloomington, United States

##### **Correspondence:** R. Xu – Cardiovascular Section/Metabolic, Cardiovascular and Inflammatory Disease Genomics Branch, National Human Genome Research Institute/National Institutes of Health, Bethesda

**Objectives:** African Americans experience disproportionately higher prevalence of type 2 diabetes and related risk factors. Little research has been done on the association of *ADIPOQ* gene on type 2 diabetes, plasma adiponectin, blood glucose, HOMA-IR and body mass index (BMI) in African Americans. The objective of our research was to assess such associations with selected SNPs. The study included a sample of 3,020 men and women from the Jackson Heart Study who had *ADIPOQ* genotyping information.

**Methods:** Unadjusted and adjusted regression models with covariates were used with type 2 diabetes and related phenotypes as the outcome stratified by sex. There was no association between selected *ADIPOQ* SNPs with type 2 diabetes, blood glucose, or BMI in men or women.

**Results:** There was a significant association between variant rs16861205 and lower adiponectin in women with minor allele A in the fully adjusted model (β(SE) p = -.13(0.05), 0.003). There was also a significant association with variant rs7627128 and lower HOMA-IR among men with minor allele A in the fully adjusted model (β(SE) p = -0.74(0.20), 0.0002).

**Conclusion:** These findings represent new insights regarding the association of ADIPOQ gene and type 2 diabetes and related phenotypes in African American men and women.

**Competing interests**

None declared.

## P49 Common variants in casr gene are associated with serum calcium levels in koreans

### S. H. Jung, N. Vinayagamoorthy, S. H. Yim, Y. J. Chung

#### Integrated Research Center for Genome Polymorphism, The Catholic University of Korea, Seoul, Korea, Republic Of

##### **Correspondence:** S. H. Jung – Integrated Research Center for Genome Polymorphism, The Catholic University of Korea, Seoul, Korea, Republic Of

**Objectives:** Calcium is a universal intracellular messenger that has an important role in controlling various cellular processes. In this study, we attempts to evaluate the genetic polymorphisms that affect serum calcium levels in Korean population through a two-stage genome-wide association study with the sample of 8642 unrelated Koreans (4558 for discovery and 4093 for replication).

**Methods:** Study subjects were selected from an ongoing population-based study known as the Korean Genome and Epidemiology Study (KoGES) and genotyped using the Affymetrix Genome-Wide Human SNP Array 5.0. We applied standard quality control parameters such as SNP call rate >95%, minor allele frequency >5% and HWE P>0.001. After this quality control process, genotypes of 4558 individuals for 1219546 autosomal SNPs were used for stage 1 association analysis.

**Results:** Using SNP arrays, we discovered 963 associated SNPs in stage 1, and replicated 105 SNPs among them in stage 2. We examined them in a combined set of stage 1 and 2 samples and observed that 65 SNPs were significantly associated with serum calcium levels. Among them, rs13068893 in the CASR gene showed the strongest significance (P=3.85×10−8). Considering the high allele frequency and significance level of the rs13068893 C>G in the CASR gene, this SNP may have a key role in regulating the serum calcium level. We also successfully replicated the four loci (CASR, CSTA, DGKD and GCKR) using our data set that have been previously reported to be significantly associated with calcium levels in Europeans and Indians.

**Conclusion:** In this exome sequencing study, we identified a novel low frequency loci. We demonstrate an alternative splicing mechanism by which the *GFI1B* rs150813342 variant suppresses formation of a GFI1B isoform that preferentially promotes megakaryocyte differentiation and platelet production.

**References**

1. Association of common variants in the calcuim-sensing receptor gene with serum calcium levels in East Asians. Vinayagamoorthy N, Yim SH, Jung SH, Park SW, Kim YJ, Kim BJ, Chung YJ. J Hum Genet. 2015; 60(8):407-412

**Competing interests**

None declared.

## P50 Inference of multiple-wave population admixture by modeling decay of linkage disequilibrium with multiple exponential functions

### Y. Zhou, S. Xu

#### Max-Planck Independent Research Group on Population Genomics, CAS-MPG PARTNER INSTITUTE FOR COMPUTATIONAL BIOLOGY, Shanghai, China

##### **Correspondence:** S. Xu – Max-Planck Independent Research Group on Population Genomics, CAS-MPG PARTNER INSTITUTE FOR COMPUTATIONAL BIOLOGY, Shanghai, China

**Objectives:** To infer the histories of complex admixture, two important challenges stand out with methods based on the admixture-introduced linkage disequilibrium (LD): getting rid of the effect of confounding LD (CLD) brought by source populations and fitting LD decay induced by admixture. In previous studies, the decay curve of weighted LD between pairs of sites whose genetic distance were bigger than a certain starting distance was fitted by single or multiple exponential functions, for the inference of single- or multiple-wave of admixture.

**Methods:** In this study, we developed a new LD based algorithm, named MALDmef, to date the multiple-wave admixtures. Different from previous software, MALDmef takes advantage of derived source populations to reduce the effect of CLD and fits the remaining weighted LD decay curves with hundreds of exponential functions.

**Results:** The performance of MALDmef was evaluated by simulation and it was shown to be more accurate than MALDER, a state-of-the-art LD based method that was recently developed for similar purposes, under various admixture models. We further applied MALDmef to analyzing genome-wide data from the Human Genome Diversity Project (HGDP) and the HapMap Project. Interestingly, we were able to identify more than one admixture events in several populations, which have yet to be reported. For example, two major admixture events were identified in Xinjiang Uyghur, occurring around 27–30 generations ago and 182–195 generations ago, respectively. In an African population (MKK), three recent major admixtures occurring 13–16, 50–67, and 107–139 generations ago were detected.

**Conclusion:** Our method is a considerable improvement over other current methods and further facilitates the inference of the histories of complex population admixtures.

References

1. Ying Zhou, Kai Yuan, Yaoliang Yu, Xumin Ni, Pengtao Xie, Eric P Xing, Shuhua Xu*. Inference of multiple-wave population admixture by modeling decay of linkage disequilibrium with multiple exponential functions. doi: http://dx.doi.org/10.1101/026757

**Competing interests**

None declared.

## P51 A Bayesian framework for generalized linear mixed models in genome-wide association studies

### X. Wang, V. Philip, G. Carter

#### System Genetics, The Jackson Laboratory, Bar Harbor, USA

##### **Correspondence:** X. Wang – System Genetics, The Jackson Laboratory, Bar Harbor, USA

**Objectives:** Recent technical and methodological advances have greatly expanded genome-wide association studies (GWAS). The advent of low-cost whole-genome sequencing facilitates high-resolution variant identification, and the development of linear mixed models (LMM) allows improved identification of putatively causal variants. While essential for correcting false positive associations due to population stratification, LMMs have been restricted to numerical variables. However, phenotypic traits in association studies are often categorical, coded as binary case–control or ordered variables describing disease stages. Furthermore, optimally integrating the results of prior studies remains a methodological challenge.

**Methods:** To address these issues, we have devised a method for genomic association studies that implements a generalized linear mixed model (GLMM) in a Bayesian framework, called Bayes-GLMM. Bayes-GLMM has four major features: support of categorical variables; cohesive integration of previous GWAS results for related traits by Bayesian modeling; correction for sample relatedness by mixed modeling; and model estimation by both MCMC sampling and maximal likelihood estimation.

**Results:** To demonstrate our method, we applied Bayes-GLMM to the whole-genome sequencing cohort in the Alzheimer’s Disease Sequencing Project (ADSP). This study contains 576 individuals distributed across 111 families, each with Alzheimer’s disease diagnosed at four confidence levels. The profound population structure in these data required a mixed model approach, and the categorical trait necessitated a generalized model.

**Conclusion:** In summary, this work provides the first implementation of a flexible, generalized mixed model approach in a Bayesian framework.

**Competing interests**

None declared.

## P52 Targeted sequencing approach for the identification of the genetic causes of hereditary hearing impairment

### A. A. Abuzenadah^1^, M. Gari^1^, R. Turki^2^, A. Dallol^1^

#### ^1^Center of Innovation in Personalized Medicine, Faculty of Applied Medical Sciences, King Abdulaziz University, Saudi Arabia; ^2^Ob/Gyn, King Abdulaziz University Hospital, Jeddah, Saudi Arabia

##### **Correspondence:** A. A. Abuzenadah – Center of Innovation in Personalized Medicine, Faculty of Applied Medical Sciences, King Abdulaziz University, Saudi Arabia

**Objectives:** Hearing loss is one of the most common afflictions in the world affecting about one in every 1000 newborns (Petersen and Willems 2006). Genetic factors are estimated to be the underlining cause of more than half of the hearing loss cases. The majority of hereditary hearing loss cases is not associated with syndromes (nonsyndromic hearing loss; NSHL) which can be transmitted in an autosomal recessive, autosomal dominant or X-linked modes of inheritance (Petersen and Willems 2006). At least half of the hereditary NSHL cases are caused by mutations in the GJB2/CONNEXIN26 gene (Hereditary Hearing Loss Home Page, http://webh01.ua.ac.be/hhh/). However, the contribution of GJB2 mutation to NSHL in the Kingdom of Saudi Arabia is minimal (Al-Qahtani et al. 2009) where the rate of children affected with sensineuronal hearing loss was estimated to be approximately 26 children out of 1000 (Bafaqeeh et al. 1994). NSHL can be caused by mutations affecting any one of over 80 deafness loci identified so far making NSHL a very heterogenous trait and complicates diagnosis and genetic counseling (Hilgert et al. 2009).

**Methods:** The recent advancements in targeted sequencing technologies have made it feasible to sequence multiple genes at a reasonably low cost. Therefore we have designed a targeted sequencing panel using the Ampliseq technology to amplify and sequence 84 genes known to cause NSHL. Genes were selected and custom primers were designed and manufactured through the Ampliseq portal (http://ampliseq.com).

**Results:** The design resulted in a coverage of 97.42% generating 2697 amplicons with a size range of 125–275 bp in two pools and generating 500.44 kb of DNA sequence.

**Conclusion:** This panel, which we termed OtoScan will be a useful front line genetic screening tool that will speed up the identification of many genetic causes of hereditary deafness in the Kingdom of Saudi Arabia.

**Competing interests**

None declared.

## P53 Identification of enhancer sequences by ATAC-seq open chromatin profiling

### A. Uyar^1^, A. Kaygun^2^, S. Zaman^3^, E. Marquez^1^, J. George^1^, D. Ucar^1^

#### ^1^The Jackson Laboratory for Genomic Medicine, Farmington, USA; ^2^Department of Mathematical Engineering, Istanbul Technical University, Istanbul, Turkey; ^3^Department of Biomedical Engineering, University of Connecticut, Storrs, USA

##### **Correspondence:** A. Uyar – The Jackson Laboratory for Genomic Medicine, Farmington, USA

**Objectives:** Enhancers are cis-regulatory elements that regulate gene expression in the control of cell type-specific functions, developmental fate and evolution. Recent studies revealed the importance of enhancer misregulation for the pathogenesis of certain diseases including cancer through point mutations in either regulatory elements or factors modulating enhancer-promoter communication. Enhancers are often located far from their target gene promoters within the noncoding genome, which makes their identification challenging. Using statistical models, such as ChromHMM, we can infer enhancer locations by studying multiple histone modifications obtained by the ChIP-seq technology. However, ChIP-seq requires large cell numbers (~10M), which is not compatible with clinical samples. The Assay for Transposase Accessible Chromatin (ATAC-seq) is an alternative assay to generate chromatin accessibility maps from 500–5,000 cells, making this an ideal approach to study enhancers in clinical samples. Here we developed a machine-learning model to infer the entire repertoire of enhancers from ATAC-seq open chromatin profiles.

**Methods:** We conduct our analysis on ATAC-seq data generated from GM12878 human lymphoblastoid cell line and CD4+ T-cells. From these samples, we extracted i) ATAC-seq features, e.g., peak strength; ii) DNA sequence based features, e.g., conservation scores; and iii) TF binding features using footprinting algorithms and compiled a comprehensive dataset. We applied Random Forest algorithm on this data to predict reference enhancers obtained from ChromHMM models.

**Results:** Our final data include ~60K ATAC-seq peaks and 490 features and a class indicating chromatin state obtained via ChromHMM. Random Forest based model predicted enhancer sequences with 74.8% sensitivity and 73.8% specificity. We also showed that the most important features for accurately predicting enhancer sequences include ATAC-seq based features and certain TF binding profiles.

**Conclusion:** The present study demonstrated that prediction of regulatory elements using ATAC-seq data alone is comparable to ChromHMM predictions. It is noteworthy that ChromHMM states are also predictions and the actual performance of ATAC-seq based model needs to be tested on experimentally validated enhancers. Our future work includes comparing our predictions with known enhancers from public databases such as FANTOM5 and Vista enhancers.

**Competing interests**

None declared.

## P54 Direct enrichment for the rapid preparation of targeted NGS libraries

### C. L. Hendrickson^1^, A. Emerman^1^, D. Kraushaar^1^, S. Bowman^1^, N. Henig^1^, T. Davis^2^, S. Russello^2^, K. Patel^1^

#### ^1^Directed Genomics, Ipswich, USA; ^2^New England Biolabs, Ipswich, USA

##### **Correspondence:** C. L. Hendrickson – Directed Genomics, Ipswich, USA

**Objectives:** Target enrichment, coupled with next-generation sequencing, enables the interrogation of specific targets of interest at a level of sensitivity that is typically cost-prohibitive with whole genome sequencing. However, the drawbacks to target enrichment generally include longer and more complex library preparation workflows or a loss of the ability to detect PCR duplicates. Here we present DIRECT target enrichment, a unique and highly specific method for hybridization-based target enrichment that incorporates the use of unique molecular indexes in a simple, one-day protocol.

**Methods:** DNA was isolated from fresh frozen tissue, FFPE samples, and liquid biopsies followed by enrichment of 36 kb of cancer-related targets with the DIRECT enrichment method. Combined with unique molecule barcoding and Illumina sequencing, variants were detected and frequencies reported using common variant callers.

**Results:** Application of DIRECT target enrichment across different DNA sample types show similar results, with no loss in specificity or sensitivity across DNA isolated from different sources. Somatic variations can be detected with high sensitivity using as little as 50 ng of input DNA, and libraries can be made with as little as 10 ng of starting material for the detection of genetic variations.

**Conclusion:** DIRECT target enrichment enables the highly specific capture of regions of interest within 7 hours and with low amounts of starting material. As a result, this method can easily be applied to a variety of applications for which time and sample amount may be limiting.

**Competing interests**

None declared.

## P56 Performance of the Agilent D5000 and High Sensitivity D5000 ScreenTape assays for the Agilent 4200 Tapestation System

### R. Nitsche^1^, L. Prieto-Lafuente^2^

#### ^1^Agilent Technologies, Agilent Technologies, Waldbronn, Germany; ^2^Agilent Technologies UK Ltd, Agilent Technologies UK Ltd. Edinburgh, UK

##### **Correspondence:** R. Nitsche – Agilent Technologies, Agilent Technologies, Waldbronn, Germany

**Objectives:** This Poster focuses on the performance of both D5000 ScreenTape assays with respect to the accuracy and precision of quantification and sizing, as well as the sensitivity of these assays. Data analysis for quantification and molarity determination was compared against the corresponding assay for the Agilent 2100 Bioanalyzer system. Additionally, performance of both the D5000 and High Sensitivity D5000 assays on the 4200 TapeStation was compared to the 2200 TapeStation system.

**Methods:** The 4200 and 2200 TapeStation systems were used with the D5000 and High Sensitivity D5000 ScreenTape assays.

**Results:** Data is presented that prove the specifications of sensitivity, reproducibility, accuracy and linearity of the new D5000 assay for the Agilent 4200 TapeStation system.

**Conclusion:** This Poster shows that the Agilent D5000 ScreenTape and Agilent High Sensitivity D5000 ScreenTape assays for the Agilent 4200 TapeStation system provide highly accurate and reproducible sizing and quantification of DNA fragments ranging from 100 to 5,000 bp. Furthermore, it demonstrates that the assays can also be applied to determine the DNA average region size, molarity, and concentration of distributed DNA smears. The sizing, concentration, and molarity results highly correlate with the data obtained from equivalent assays on the Agilent 2100 Bioanalyzer system. In addition, D5000 and High Sensitivity D5000 ScreenTape assays show equivalent performance in the 4200 and Agilent 2200 TapeStation systems.

**Competing interests**

R. Nitsche Employee of: Agilent Technologies, L. Prieto-Lafuente Employee of: Agilent Technologies

## P57 ClinVar: a multi-source archive for variant interpretation

### M. Landrum, J. Lee, W. Rubinstein, D. Maglott

#### NIH/NLM/NCBI, Bethesda, USA

##### **Correspondence:** M. Landrum – NIH/NLM/NCBI, Bethesda, USA

**Objectives:** ClinVar archives submitted interpretations of the medical importance of human variants, standardizes content, and aggregates data from multiple sources to facilitate peer review and track revisions.

**Methods:** Interpretations are provided by clinical testing laboratories, research laboratories, OMIM®, GeneReviews™, locus-specific databases, expert panels and organizations that provide practice guidelines. ClinVar integrates those data with information from other NCBI databases such as dbSNP, dbVar, Gene, and MedGen.

**Results:** The ClinVar website can be searched with gene symbols, variant names including HGVS expressions, rs numbers, and diseases or phenotypes. Searches with many results may be narrowed with filters, such as restricting to a clinical significance, type of variant, and more. Searching by location is facilitated by Variation Viewer, a browser to view all NCBI variation data in a region of interest, such as a region of structural variation. Each row of the search results table in ClinVar links to a page for that variant(s), which has two major sections: aggregate data and submitter-specific data. The aggregate data section provides a summary of information about the interpreted variant(s). The summary includes the review status for the variant(s), represented with a description and a number of gold stars, and a variation identifier, which is a unique identifier that represents the variant(s) that were interpreted. The summary also displays an overall clinical significance, which is calculated based on interpretations provided by submitters; ClinVar staff do not curate or review clinical significance. A list of conditions for which the variant was interpreted is provided, as well as general information about the variant including links to other public databases. The submitter-specific section includes details of the assertions of clinical significance and evidence provided by each submitter. Evidence may include citations, counts of individuals with the variant, free text descriptions of the evidence, and more. Data is updated only by the submitter; thus maintenance of an interpretation and the supporting evidence is the responsibility of each submitter.

**Conclusion:** Those with new evidence and interpretations are encouraged to contribute their own data to improve content for all. An online Submission Portal is available to upload data to ClinVar, as a single submission or in batch. ClinVar provides monthly releases available via FTP, and API to access specific records.

**Competing interests**

None declared.

## P59 Association of functional variants and protein physical interactions of human MUTY homolog linked with familial adenomatous polyposis and colorectal cancer syndrome

### Z. Abduljaleel^1^, W. Khan^2^, F. A. Al-Allaf^1^, M. Athar^1^, M. M. Taher^1^, N. Shahzad^1^

#### ^1^Department of Medical Genetics, Faculty of Medicine, Umm Al-Qura University, Makkah, Saudi Arabia, ^2^Department of Basic Sciences, College of Science and Health Professions, King Saud Bin Abdul Aziz University for Health Sciences, Riyadh, Saudi Arabia

##### **Correspondence:** Z. Abduljaleel – Department of Medical Genetics, Faculty of Medicine, Umm Al-Qura University, Makkah, Saudi Arabia

**Objectives:** To the best of our knowledge, this is the first report confirming the structural and functional association of MUTYH mutations (Y165C; G382D; P54S; A22V; Q63R; G45D; S136P and N43S) through *In-silico* approach.

**Methods:** We retrieved clinical records of MUTYH for multiple adenomas, test unit Code 84304 from the Mayo Medical Laboratory, USA.

The nonsynonymous single nucleotide polymorphisms (nsSNPs) may alter the structure or function of expressed proteins, and therefore have an effect on disease outcome. In order to evaluate the phenotypic effects of nsSNPs in human DNA repair genes, we studied the polymorphism in terms of various functional properties.

The molecular origin of disease-related phenotypes was caused by six mutations in MUTYH, and that were associated with heritable predisposition of colon and stomach cancer syndrome of FAP.

**Results:** We confirmed that MAP was inherited in an autosomal recessive pattern due to the mutations in Mutyh (P54S, A22V, Q63R, G45D, S136P and N43S), hence both copies of the gene become inactivated during disease occurance. The parents of an individual with an autosomal recessive condition may serve as carriers, each harboring one copy of the mutated gene without showing signs and symptoms of MAP. The MUTYH protein interacts with six partners, but only four of these proteins showed direct physical interactions in our study. These proteins were hMSH6, hPCNA, hRPA1, and hAPEX1. We also for the first time examined specific interactions of these protein partners with MAP associated MUTYH mutants using molecular dynamics simulations. These approaches provided tools for exploration of the conformational energy landscape accessible to these protein partners. The study also determined the impact before and after energy minimization of protein-protein interaction (PPI) and binding affinities of MUTYH wild type and mutant forms, including interactions with other proteins. Taken together, this study has provided innovative insights into the role of MUTYH and its interacting proteins in MAP.

**Conclusion:** This study provides interesting information and will open up a fresh avenue for the FAP researchers. We also strongly believe that the identified important features of the HMUTYH mutations through our *In-silico* study will support further In vitro studies in future.

**Competing interests**

None declared.

## P60 Modification of the microbiom constitution in the gut using chicken IgY antibodies resulted in a reduction of acute graft-versus-host disease after experimental bone marrow transplantation

### A. Bouazzaoui^1,2,3^, E. Huber^4^, A. Dan^5^, F. A. Al-Allaf^6^, W. Herr^3^, G. Sprotte^7^, J. Köstler^8^, A. Hiergeist^8^, A. Gessner^8^, R. Andreesen^3^, E. Holler^3^

#### ^1^Science and Technology Unit, Umm Al Qura University, Mecca, Saudi Arabia; ^2^Department of Medical Genetics, Umm Al Qura University, Mecca, Saudi Arabia; ^3^Medical Clinic 3 – Hematology/Oncology, University Hospital Regensburg, Regensburg, Germany; ^4^Department of Pathology, University Hospital Regensburg, Regensburg, Germany; ^5^IgNova GmbH, Oberursel, Germany; ^6^Department of Medical Genetics Faculty of Medicine, Umm Al Qura University, Mecca, Saudi Arabia; ^7^Department of Ansethesiologie, University of Würzburg Medical School, Würzburg, Germany; ^8^Department of microbiology, University Hospital Regensburg, Regensburg, Germany

##### **Correspondence:** F. A. Al-Allaf - Science and Technology Unit, Umm Al Qura University, Mecca, Saudi Arabia

**Objectives:** GVHD remains the major cause of morbidity and mortality after allogeneic stem cell transplantation. This is particularly true for intestinal GVHD, therefore new methods are urgently needed. One approach is the reduction of pathogenic bacteria.

**Methods:** Using a haploidentical murine model, B6D2F1 mice conditioned with total body irradiation (TBI) received bone marrow cells and splenocytes from either syngeneic (B6D2F1) or allogeneic (C57BL/6) donors. After that, animals received from day -2 until day +28 chow with egg yolk (IgY) containing antibodies again E. coli, S. typhimurium and C. perfringens (in alternative protocol, mice received chow from day -2 until day 15). As control the mice received chow without egg yolk. After treatment the effect on the severity of aGVHD, cytokines, chemokines and pathogen-associated molecular patterns products (PAMP) were analyzed. Furthermore the bacterial load and microbial constitution in the stool were determined using real time PCR and new generation sequencing method.

**Results:** Animals received IgY chow showed reduced GVHD severity, improved survival and less organ damages compared to control animals. The improvement was associated with significantly diminished expression of TNF, IL-2 and IL-6 levels in the serum and correlated with decreased bacteria load in the stool, especially E. coli. Interestingly, the bacterium L. reuteri showed increased load in IgY treated animals. This result is in line with early works showed that Lactubacillus mediated protection again aGVHD (1, 2). The expression of TLR2, 4 and NOD2 was also reduced in the gut of IgY mice.

**Conclusion:** Feed pellets with egg yolk containing chicken antibodies (IgY) improved GVHD and reduced the cytokine, chemokine and PAMP expression. Furthermore IgY antibodies decreased the pathogenic and improved the probiotic bacteria in the colon.

**References**

1: Gerbitz A et a. Blood. 2004,

2: Jenq RR et al. J Exp Med. 2012

**Disclosure of interest**

None declared.

## P61 Compound heterozygous mutation in the *LDLR* gene in Saudi patients suffering severe hypercholesterolemia

### F. Al-Allaf^2,3^, A. Alashwal^4^, Z. Abduljaleel^1,2^, M. Taher^1,2^, A. Bouazzaoui^1,2^, H. Abalkhail^4^, A. Al-Allaf^5^, R. Bamardadh^2^, M. Athar^1,2^

#### ^1^Department of Medical Genetics, Faculty of Medicine, Umm Al-Qura University, Saudi Arabia; ^2^Science and Technology Unit, Umm Al-Qura University, Saudi Arabia; ^3^Molecular Diagnostics Unit, Department of Laboratory and Blood Bank, King Abdullah Medical City, Makkah, Saudi Arabia; ^4^King Faisal Specialist Hospital and Research Centre, Saudi Arabia; ^5^Faculty of Medicine, Alfaisal University, Riyadh, Saudi Arabia

##### **Correspondence:** F. Al-Allaf - Department of Medical Genetics, Faculty of Medicine, Umm Al-Qura University, Saudi Arabia

**Objectives:** Familial hypercholesterolemia (FH) is most commonly caused by variants in the LDL receptor (*LDLR*) gene. Herein, we describe a severely affected FH proband and their first-degree blood relatives, the proband was resistant to statin therapy and was managed on an apheresis program.

**Methods:** Genetic screening for the *LDLR* variants was performed by exon sequencing analysis. Using a bioinformatics approach we theoretically explored the putative structure of the wild type and mutant LDLR protein and the implication of structural changes on the possibility of LDLR dimer formation.

**Results:** We identified a compound heterozygous mutation with missense c.1731G>T, p.(W577C) and frameshift c.2027delG, p.(G676Afs*33) variants at exons 12 and 14 of the *LDLR* gene respectively in a proband of a Saudi family. DNA sequencing of *LDLR* from the parents demonstrated that the missense variant was inherited from the mother and frameshift variant was inherited from the father. The frameshift variant resulted in a stop signal 33 codons downstream of the deletion, which most likely lead to a truncated protein that lacks important functional domains, including the trans-membrane domain and the cytoplasmic tail domain. The missense variant is also predicted to be likely pathogenic and affect EGF-precursor homology domain of the LDLR protein. The segregation pattern of the variants is consistent with the lipid profile, suggesting a more severe FH phenotype when the variants are in the compound heterozygous state. We also describe a three dimensional homology model of LDLR structure and examine the consequence of the missense and frameshift variants, as this could affect the LDLR structure in a region involved in dimer formation, and protein stability.

**Conclusion:** The finding of a compound heterozygous mutation causing severe FH phenotype is important for the genotype-phenotype correlation. Furthermore, provided bioinformatics based structural analysis of the mutant protein and its molecular consequences on the physiology of FH that could facilitate developing mechanistic models of LDLR function. These results also enlarge the spectrum of FH-causative *LDLR* variants in the Arab population, including the Saudi population.

**Disclosure of interest**

None declared.

